# Sequencing of Candidate Chromosome Instability Genes in Endometrial Cancers Reveals Somatic Mutations in *ESCO1*, *CHTF18*, and *MRE11A*

**DOI:** 10.1371/journal.pone.0063313

**Published:** 2013-06-03

**Authors:** Jessica C. Price, Lana M. Pollock, Meghan L. Rudd, Sarah K. Fogoros, Hassan Mohamed, Christin L. Hanigan, Matthieu Le Gallo, NIH Intramural Sequencing Center (NISC) Comparative Sequencing Program, Suiyuan Zhang, Pedro Cruz, Praveen F. Cherukuri, Nancy F. Hansen, Kirk J. McManus, Andrew K. Godwin, Dennis C. Sgroi, James C. Mullikin, Maria J. Merino, Philip Hieter, Daphne W. Bell

**Affiliations:** 1 Cancer Genetics Branch, National Human Genome Research Institute, National Institutes of Health, Bethesda, Maryland, United States of America; 2 Intramural Sequencing Center, National Institutes of Health, Bethesda, Maryland, United States of America; 3 Genome Technology Branch, National Human Genome Research Institute, National Institutes of Health, Bethesda, Maryland, United States of America; 4 Department of Biochemistry and Medical Genetics, University of Manitoba, Manitoba Institute of Cell Biology, Winnipeg, Manitoba, Canada; 5 Department of Pathology and Laboratory Medicine, University of Kansas Medical Center, Kansas City, Kansas, United States of America; 6 The University of Kansas Cancer Center, University of Kansas Medical Center, Kansas City, Kansas, United States of America; 7 Molecular Pathology Unit and Center for Cancer Research, Massachusetts General Hospital, Charlestown, Massachusetts, United States of America; 8 Center for Cancer Research, National Cancer Institute, National Institutes of Health, Bethesda, Maryland, United States of America; 9 Michael Smith Laboratories, University of British Columbia, Vancouver, British Columbia, Canada; Dartmouth, United States of America

## Abstract

Most endometrial cancers can be classified histologically as endometrioid, serous, or clear cell. Non-endometrioid endometrial cancers (NEECs; serous and clear cell) are the most clinically aggressive of the three major histotypes and are characterized by aneuploidy, a feature of chromosome instability. The genetic alterations that underlie chromosome instability in endometrial cancer are poorly understood. In the present study, we used Sanger sequencing to search for nucleotide variants in the coding exons and splice junctions of 21 candidate chromosome instability genes, including 19 genes implicated in sister chromatid cohesion, from 24 primary, microsatellite-stable NEECs. Somatic mutations were verified by sequencing matched normal DNAs. We subsequently resequenced mutated genes from 41 additional NEECs as well as 42 endometrioid ECs (EECs). We uncovered nonsynonymous somatic mutations in *ESCO1*, *CHTF18,* and *MRE11A* in, respectively, 3.7% (4 of 107), 1.9% (2 of 107), and 1.9% (2 of 107) of endometrial tumors. Overall, 7.7% (5 of 65) of NEECs and 2.4% (1 of 42) of EECs had somatically mutated one or more of the three genes. A subset of mutations are predicted to impact protein function. The co-occurrence of somatic mutations in *ESCO1* and *CHTF18* was statistically significant (*P* = 0.0011, two-tailed Fisher's exact test). This is the first report of somatic mutations within *ESCO1* and *CHTF18* in endometrial tumors and of *MRE11A* mutations in microsatellite-stable endometrial tumors. Our findings warrant future studies to determine whether these mutations are driver events that contribute to the pathogenesis of endometrial cancer.

## Introduction

Uterine cancer is the most commonly diagnosed gynecologic malignancy in the United States and is the eighth leading cause of death from cancer among American women [1]. Endometrial cancers (ECs) account for the vast majority of uterine cancers. Endometrioid, serous, and clear cell carcinomas represent the three major histological subtypes of EC. Each subtype arises from distinct precursor lesions, has distinct clinical behaviors and distinct molecular etiologies [2], [3].

Endometrioid ECs (EECs) are estrogen-dependent tumors associated with an overall favorable prognosis evidenced by a 5-year relative survival rate of ∼90% [4]. In contrast, serous and clear cell ECs (non-endometrioid ECs (NEECs)) are clinically aggressive, estrogen-independent tumors with 5-year relative survival rates of only 44% and 65% respectively [4]. NEECs contribute disproportionately to mortality from EC. In a population-based study of endometrioid, serous, and clear cell ECs within the United States Surveillance Epidemiology and End Results (SEER) program (1988–2001), NEECs accounted for 47% of deaths even though they constituted only 13% of diagnoses [5].

EECs and NEECs exhibit distinct modes of genomic instability. EECs tend to be diploid or near-diploid but frequently exhibit microsatellite instability (MSI) [6], [7], [8], [9], [10], [11]. In contrast, NEECs are frequently aneuploid, or chromosomally unstable, but display MSI only rarely [11], [12], [13], [14], [15], [16], [17].

MSI reflects a mutator phenotype resulting from defective mismatch repair (reviewed in [18]). In sporadic endometrial cancers, most instances of MSI are explained by hypermethylation of the MLH1 promoter, loss of MSH2 expression, or somatic mutations in *MSH6* (reviewed in [19]). Aneuploidy has recently been suggested to result from a step-wise process resulting from an acquired tolerance for a non-diploid genome, via inactivation of the p53 pathway, as well as aberrant chromosome segregation [20]. Although inactivating mutations in *TP53* and p53 protein stabilization are frequent in NEECs, occurring in up to 90% of serous tumors (reviewed in [19]), the genetic basis of chromosome missegregation in NEECs remains poorly understood.

In yeast, chromosome missegregation can arise from mutations in genes that regulate sister-chromatid cohesion [21], [22]. Mitotic sister chromatid cohesion refers to the physical linkage of replicated sister chromatids by the cohesin protein complex until anaphase, to ensure the faithful segregation of sister chromatids into daughter cells. In *S. cerevisiae*, the cohesin complex consists of the Smc1, Smc3, Scc1, and Scc3 subunits and is loaded onto chromatin at the end of G1 by a process that requires the Scc2-Scc4 complex [23], [24], [25]. Subsequent cohesion establishment depends upon the acetylation of Smc3 by the Eco1 acetyltransferase [26], [27], [28], as well as the activities of Chl1 and the alternative replication factor C (Rfc) complex Ctf18-Ctf8-Dcc-Rfc [21], [29]. Cohesion establishment is antagonized by the activities of the Wpl1-Pds5 complex and the Elg1-Rfc complex [30], [31].

The proteins that regulate sister chromatid cohesion are highly conserved throughout evolution. In mammalian cells, the mitotic cohesin complex is formed by SMC1A (hSmc1), SMC3 (hSmc3), RAD21 (hScc1), and SA1/SA2 (hScc3). Cohesin loading is dependent upon NIPBL (hScc2) and MAU2 (hScc4) (reviewed in [32]). Cohesion establishment requires acetylation on SMC3 by the ESCO1 and ESCO2 acetyltransferases [33] and is also regulated by the CHTF18-RFC complex [34] and by DDX11 (hChl1) [35], [36].

There is a growing body of evidence implicating the mutational disruption of sister chromatid cohesion genes in human cancer. Somatic deletions and mutations of several genes that regulate sister chromatid cohesion have recently been uncovered in colorectal cancer, Ewing's sarcoma, glioblastoma, melanoma, acute myeloid leukemia, and myeloid diseases [37], [38], [39], [40], [41], [42], [43]. We previously described somatic loss-of-function mutations of *ATAD5* in endometrial cancers [44]. ATAD5 is the human orthologue of *S. cerevisiae* Elg1, which forms an Rfc-like complex that participates in sister chromatid cohesion [45], [46].

In the present study, we sought to determine whether additional sister chromatid cohesion genes are somatically mutated in endometrial tumors. We resequenced the human orthologues of 19 genes implicated in the regulation of sister chromatid cohesion, as well as two additional candidate chromosome instability (CIN) genes, from 24 primary NEECs. Mutated genes were subsequently sequenced from 83 additional endometrial tumors. Our study uncovered nonsynonymous somatic mutations in *ESCO1*, *CHTF18*, and *MRE11A* in a subset of human endometrial tumors.

## Materials and Methods

### Ethics statement

The NIH Office of Human Subjects Research determined that this research was not “human subjects research” per the Common Rule (45 CFR 46), and therefore that no IRB review was required for sequencing of the anonymized samples in this study.

### Clinical specimens

Anonymized, primary endometrial tumor tissues (45 serous, 20 clear cell, and 42 endometrioid) and matched histologically normal tissues were obtained from the Cooperative Human Tissue Network, or from the Biosample Repository at Fox Chase Cancer Center, Philadelphia PA. Six cases of matched tumor and normal DNAs were procured from Oncomatrix. All tumor tissues were collected before treatment. An hematoxylin and eosin (H&E) stained section of each tumor specimen was reviewed by a pathologist to verify histology and to delineate regions of tissue with high (≥70%) tumor cell content.

### Nucleic acid isolation and identity testing

Genomic DNA was isolated from macrodissected tissue using the Puregene kit (Qiagen). Paired, tumor-normal DNAs were genotyped using the Coriell Identity Mapping kit (Coriell) according to the manufacturer's instructions. Genotyping fragments were size separated on an ABI-3730*xl* DNA analyzer (Applied Biosystems) and alleles were scored using GeneMapper (Applied Biosystems).

### Identification of orthologous genes

A consolidated list of known and candidate human orthologues of yeast chromosome stability genes (with demonstrated roles in sister chromatid cohesion) was identified through standard cross-species approaches. Briefly, InParanoid 7 and HomoloGene databases were queried to identify known orthologues, while BLASTp was employed to identify the top-hit candidates (based on E-value) from the non-redundant protein sequences within the *Homo sapiens* database.

### Reverse transcriptase PCR (RT-PCR)

Total RNA was extracted from 5 endometrioid and 2 serous endometrial cancer cell lines using Trizol Reagent (Ambion). A commercially available human total RNA control mix (Applied Biosystems) was used as a positive control. cDNA synthesis was performed on 1μg of total RNA with the high-capacity cDNA archive kit using random hexamers (Applied Biosystems). cDNAs (0.2μl) were amplified by PCR using the primer pairs provided in **Table S1**. Amplification consisted of 40 cycles using the following parameters: 94°C for 30****s, 58°C for 30****s and 72°C for 30****s, with a final extension step at 72°C for 10****min. PCR products were separated on a 1% agarose gel stained with ethidium bromide in 0.5× TAE buffer and visualized under ultraviolet illumination.

### Cell lines and Western blot analysis

Serous endometrial cancer cell lines (ARK1 and ARK2) were kindly provided by Dr. Alessandro Santin (Yale School of Medicine). Endometrioid endometrial cancer cell lines (RL-95-2, HEC1A, HEC1B, ANC3A) and a cell line derived from a poorly differentiated endometrial adenocarcinoma (KLE) were obtained from the American Type Culture Collection, or the NCI Developmental Therapeutics Program cell line repository. Cells were washed in phosphate-buffered saline followed by lysis in ice-cold RIPA buffer (Thermo Scientific) containing 1****mM Na-orthovanadate, 10****mM NaF, and 1X protease inhibitor cocktail (Roche). Lysates were centrifuged and equal amounts of the cleared lysate were denatured at 95°C in 2× SDS sample buffer (Sigma) prior to SDS-PAGE and transfer to PVDF membranes (Bio-Rad). Primary and HRP-conjugated secondary antibodies were: αMRE-11 (Cell Signaling), αCHTF18 (Novus Biological), αESCO1 (Novus Biological), α-α/β-Tubulin (Cell Signaling), goat anti-mouse HRP (Cell Signaling), and goat anti-rabbit HRP (Cell Signaling). Immunoreactive proteins were visualized with enhanced chemiluminescence (Pierce).

### Primer design and PCR amplification

Primer pairs were designed, using published methods [47], to target 97.4% (458 of 470) of all exons of the 21 genes in the mutation discovery screen (**Table S2**), and all exons of the three genes in the mutation prevalence screen (**Table S3)**. PCR conditions are available on request.

### Nucleotide sequencing

PCR products were subjected to bidirectional Sanger sequencing using M13 primers and the BigDye Terminator Version 3.1 Cycle Sequencing Kit (Applied Biosystems). Sequencing reactions were run on ABI 3730*xl* DNA Analyzers (Applied Biosystems). Sequence trace quality was assessed with the base-calling program, Phred [48], [49]. All traces were included in the subsequent analysis, since deletion-insertion polymorphisms can mimic poor quality data from a Phred-quality measure, but may contain valid sequence data. All sequences for a given primer pair were assembled using Consed [50]; overlapping amplimers were assembled separately to allow independent cross-validation of calls in overlapping regions. Sequence variants, including single-nucleotide differences and short (<100 base pair) insertions and deletions, were identified using PolyPhred v6.11 [51] and an in-house algorithm (DIPDetector) optimized for improved sensitivity in finding insertions and deletions from aligned trace data. DIPDetector analyzes Sanger sequencing traces and predicts insertions and deletions by first examining read alignments for homozygous variants. It then searches for signatures of heterozygous insertions and deletions within the output of the basecaller phred run with the – poly option [49]. After forming two vectors containing the bases with highest peak areas at each position of the read (or assigning the highest area peak to both vectors when the second largest peak has an area less than 10% the size of the largest peak), DIPDetector attempts to phase these vectors by inserting potential shifts of all possible sizes into all possible positions of the read, and scores these shifts according to how well the resulting shifted vectors match the observed bases within the trace. Human genome assembly hg18 (NCBI Build 36.1) was used as the reference sequence. Variant positions were cross-referenced to dbSNP (Build 129) entries to identify known polymorphisms. To determine whether novel variants were somatic mutations or germline polymorphisms, the appropriate tumor DNA and matched normal DNA were re-amplified in an independent PCR followed by sequence analysis of the variant position. The predicted impact of somatic mutations on protein function was evaluated *in silico* using Mutation Assessor release 2 (http://mutationassessor.org/), SIFT (http://sift.jcvi.org/), and Polyphen-2 (http://genetics.bwh.harvard.edu/pph2/index.shtml).

### Calculation of discovery screen power

The estimated power to detect one gene mutation in a set of 24 tumors is 1– (1-X)?24, where X is the actual fraction of tumors with a mutation in that gene.

## Results and Discussion

In a mutation discovery screen, we analyzed 24 primary NEECs for the presence of nucleotide variants within the coding exons and splice junctions of 21 candidate chromosome instability genes, which are expressed, at variable levels, in endometrial cancer cell lines (**Figure S1**). Nineteen of these genes are implicated in the regulation of sister-chromatid cohesion, based on their sequence homology to cohesion genes in *S. cerevisiae* (**Table 1**). The 24 NEECs consisted of 17 serous ECs and 7 clear cell ECs; five of the serous tumors (T33, T45, T65, T69, T70) were recently subjected to whole exome sequencing [52]. We included only MSI-stable tumors in the discovery screen; the MSI data have been reported elsewhere [52].

**Table 1:** Genes resequenced in the mutation discovery screen.

Human Gene Symbol	Human Gene Name	Human mRNA Accession Number	Human Protein Accession Number	*S. cerevisiae* Ortholog	E- value	*S. cerevisiae* Ortholog Regulates Cohesion
*APRIN/PDS5B*	PDS5, regulator of cohesion maintenance, homolog B	NM_015032.1	NP_055847.1	*PDS5*	2E-32	Yes
*CHTF8/DERPC*	CTF8, chromosome transmission fidelity factor 8 homolog	NM_001039690	NP_001035236.1	*CTF8*	NA	Yes
*CHTF18*	CTF18, chromosome transmission fidelity factor 18 homolog	NM_022092.1	NP_071375.1	*CTF18*	8E-42	Yes
*CSPG6/SMC3*	Structural maintenance of chromosomes 3	NM_005445.3	NP_005436.1	*SMC3*	0.0	Yes
*DDX11/CHLR1*	DEAD/H (Asp-Glu-Ala- Asp/His) box polypeptide 11	NM_030653.2	NP_085911.2	*CHL1*	2E-139	Yes
*DSCC1/DCC1*	Defective in sister chromatid cohesion 1 homolog	NM_024094.1	NP_076999.2	*DCC1*	8E-11	Yes
*ESCO1*	Establishment of cohesion 1 homolog 1	NM_052911.1	NP_443143.2	*ECO1/CTF7*	8E-15	Yes
*KIAA1212/CCDC88A*	Coiled-coil domain containing 88A	NM_018084.3	NP_060554.3	*RAD61*	4E-04	Yes
*LEO1*	Leo1, Paf1/RNA polymerase II complex component, homolog	NM_138792.2	NP_620147.1	*MRC1*	1E-04	-
*MRE11A*	MRE11 meiotic recombination 11 homolog A	NM_005591	NP_005582.1	*MRE11*	2E-130	Yes
*NIPBL*	Nipped-B homolog	NM_015384.3	NP_597677.2	*SCC2*	4E-14	Yes
*REC8L1*	REC8 homolog	NM_001048205.1	NP_005123.1	*SPO69*	9E-05	Yes
*SCC-112/PDS5A*	PDS5, regulator of cohesion maintenance, homolog A	NM_015200	NP_056015	*PDS5*	6E-32	Yes
*SGOL1*	Shugoshin-like 1	NM_001012409.1	NP 001012410.1	*SGO1*	NA	Yes
*SMC1L1/SMC1A*	Structural maintenance of chromosomes 1A	NM_006306.2	NP_006297.2	*SMC1*	1E-153	Yes
*STAG2*	Stromal antigen 2	NM_006603	NP_006594.3	*SCC3*	5E-22	Yes
*STAG3* 2	Stromal antigen 3	NM_012447.2	NP_036579.2	*SCC3*	8E-23	Yes
*TIMELESS*	Timeless homolog (Drosophila)	NM_003920.1	NP_003911.2	*TOF1*	1E-11	Yes
*TIPIN*	TIMELESS interacting protein	NM_017858.1	NP_060328.2	*CSM3*	7E-10	Yes
*WDHD1/hCTF4*	WD repeat and HMG-box DNA binding protein 1	NM_007086.1	NP_009017.1	*CTF4*	7E-21	Yes
*ZC3H13/DING* 1	Zinc finger CCCH-type containing 13	NM_015070	NP_055885.2	*PDS1*	-	-

1 Gene analyzed because it is somatically mutated in colorectal cancer.

2 Implicated in meiotic specific cohesion.

We obtained high quality sequence data for 87.6% (5.64****Mb) of bases (6.44****Mb) targeted. After excluding variants that were annotated as single nucleotide polymorphisms (SNPs) within dbSNP (Build 129), there were 109 unique nucleotide variants that represented potential somatic mutations. To determine whether these variants were somatic mutations or germline variants, we reamplified and sequenced the variant positions from the appropriate tumor DNA and matched normal DNA. Three variants were *bone fide* somatic mutations, present in the tumor DNA but absent from the matched normal DNA. The somatically mutated genes were *ESCO1* (establishment of cohesion 1 homolog 1 (*S. cerevisiae*)), *CHTF18 (*chromosome transmission fidelity factor 18 homolog (*S. cerevisiae*)), and *MRE11A* (meiotic recombination 11 homolog A (*S. cerevisiae*)); each gene was mutated in 4% (1 of 24) of NEECs in the discovery screen. Although we found no evidence for somatic mutations in the remaining 18 candidate CIN genes, it is important to acknowledge that our discovery screen has insufficient power to detect all somatic mutations present in NEECs. We estimate that in a screen of 24 NEECs, the power to detect genes that are somatically mutated in 5%, 10% or 15% of all NEECs is 71%, 92%, and 98% respectively.

We next sought to more precisely determine the frequency and spectrum of somatic mutations in *ESCO1, CHTF18,* and *MRE11A* in endometrial cancer. To do this, we performed a prevalence screen in which we resequenced the coding exons and splice sites of the three genes from an additional 28 serous tumors, 13 clear cell tumors, and 42 endometrioid tumors, unselected for MSI status.

In the combined discovery and prevalence screens, we uncovered nonsynonymous somatic mutations within *ESCO1*, *CHTF18*, and *MRE11A* in, respectively, 3.7% (4 of 107), 1.9% (2 of 107), and 1.9% (2 of 107) of endometrial tumors (**Table 2** **and Figure S2**). Overall, 7.7% (5 of 65) of NEECs and 2.4% (1 of 42) of EECs had somatic mutations in one or more of the three genes. Compared to known consensus cancer genes with established roles in endometrial cancer, and to significantly mutated cancer genes, *ESCO1*, *CHTF18*, and *MRE11A* were infrequently mutated (**Figure S3, Figure S4, Figure S5**) [44], [52], [53], [54], suggesting that these three genes are either rare pathogenic driver genes for endometrial cancer or that they are non-pathogenic genes that have acquired passenger mutations. Immunoblotting confirmed the expression of MRE11A and CHTF18 in panel of endometrial cancer cell lines (**Figure S6**); ESCO1 was variably expressed among these same cell lines.

**Table 2:** Nonsynonymous, somatic mutations in *ESCO1*, *CHTF18* and *MRE11A*, in ECs.

Gene	Case No.	Histological Subtype	MSI Status	Nucleotide Change §	Amino Acid Change ¶	Mutation Type	Mutation Assessor (Release 2) Prediction	SIFT Prediction	Polyphen-2 Prediction
*ESCO1*	T79	Serous	Stable	c.G1012T	p.E338X	Nonsense	n/a	n/a	n/a
	T113	Clear cell	Stable	c.G1075A	p.D359N	Missense	Low	Tolerated	Benign
	T77	Clear cell	Unstable	c.C1595T	p.S532L	Missense	Low	Tolerated	Probably damaging
	T88	Endometrioid	Unstable	c.C2356T	p.R786C	Missense	Medium	Affects function	Probably damaging
*CHTF18*	T79	Serous	Stable	c.G2035A	p.D679N	Missense	Low	Tolerated	Probably damaging
	T88	Endometrioid	Unstable	c.C2560T	p.R854W	Missense	Medium	Tolerated	Possibly damaging
*MRE11A*	T68	Serous	Stable	c.G2074T	p.D692Y	Missense	Low	Affects function	Probably damaging
	T3†	Serous	Stable	c.G1448A	p.R483Q	Missense	Low	Tolerated	Benign
	T3†	Serous	Stable	c.G391A	p.D131N	Missense	Medium	Affects function	Possibly damaging

† Case no T3 is also known as OM-1323.

n/a Not applicable.

§ Transcript accession numbers: *ESCO1* (NM_052911.1), *CHTF18* (NM_022092.1), *MRE11A* (NM_005591).

¶ Protein accession numbers: ESCO1 (NP_443143.2), CHTF18 (NP_071375.1), MRE11A (NP_005582.1).

*ESCO1,* which encodes a lysine acetyltransferase that is essential for the establishment of sister chromatid cohesion in mammalian cells, was somatically mutated in 2.2% (1 of 45) of serous ECs, 10% (2 of 20) of clear cell ECs, and 2.4% (1 of 42) of endometrioid ECs. Two of the *ESCO1* mutations are predicted to impact protein function. The ESCO1^R786C^ missense mutant, within the acetyltransferase domain, is predicted to impact protein function by both the SIFT and Polyphen algorithms (**Table 2**). We speculate that the ESCO1^E338X^ nonsense mutant, which we uncovered in a serous-EC, may be a loss-of function mutant since a protein produced by this allele would be prematurely truncated and fail to include the acetyltransferase domain. Alternatively, nonsense-mediated decay of the *ESCO1^E338X^* transcript might lead to haploinsufficiency.

**Figure 1:**
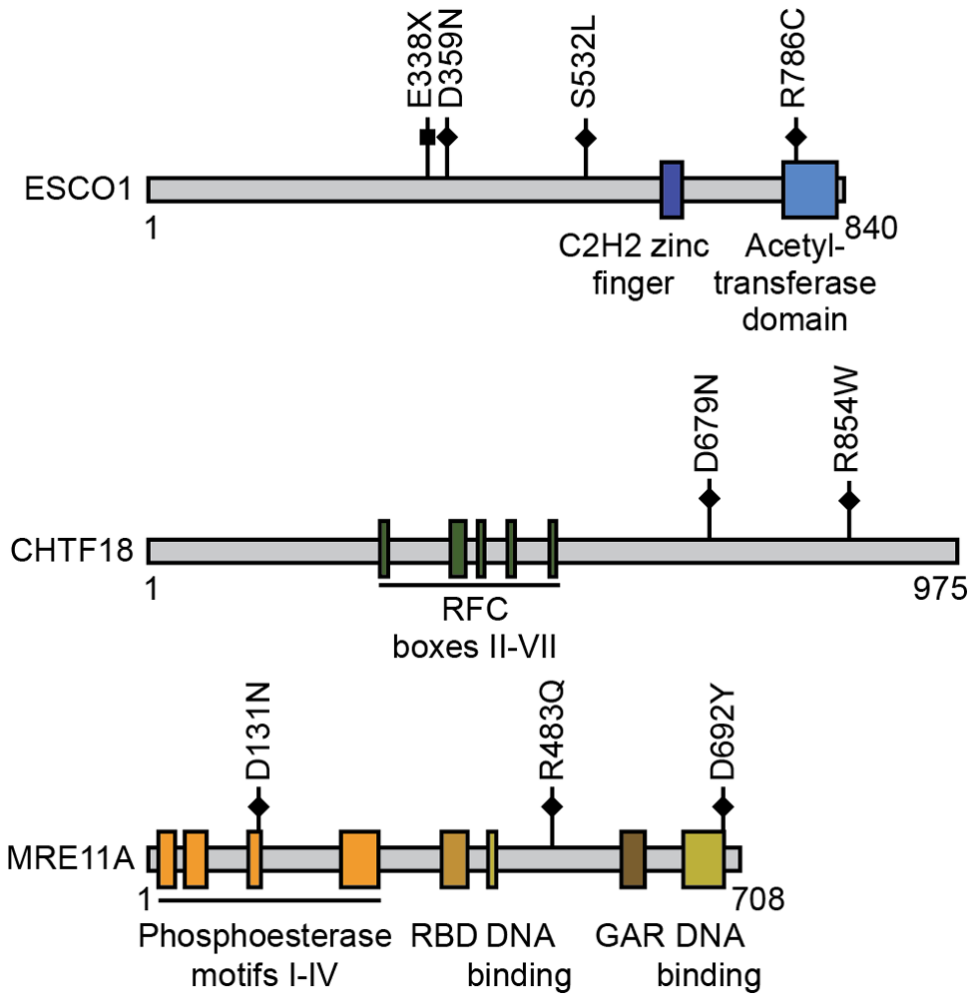
Localization of somatic mutations in *ESCO1*, *CHTF18*, and *MRE11A* in primary endometrial tumors, relative to important functional domains of the encoded proteins. Individual somatic mutations are indicated by squares (nonsense mutations) or diamonds (missense mutations). Domain positions are derived from [65], [66], [61], [59], [67]. GAR: Glycine-Arginine-Rich motif; RBD:RAD50 Binding Domain; RFC box: Replication Factor C box.

*CHTF18* was somatically mutated in 2.2% (1 of 45) of serous ECs and 2.4% (1 of 42) of endometrioid ECs. In human cells, the CHTF18-RFC complex regulates the acetylation of the SMC3 cohesion-subunit by ESCO1 and ESCO2 acetyltransferases [34], thereby contributing to the establishment of sister chromatid cohesion. The CHTF18-RFC complex has also been implicated in the stimulation of DNA polymerase η activity, and in the recruitment of DNA polymerase ε to sites of gap-filling repair synthesis [55], [56]. Both of the CHTF18 mutants we uncovered in endometrial cancer localize to the carboxy-terminus of the protein (**Figure 1**), within a region (residues 576-876) that mediates binding to RFC2-5 [57]. The CHTF18^R854W^ mutant is predicted to possibly affect protein function by the Mutation Assessor and SIFT algorithms (**Table 2**). Interestingly, the majority of *CHTF18* mutations observed in other cancers also localize to the C-terminus of the encoded protein [58]. These observations raise the possibility that somatic missense mutations in the C-terminus of CHTF18, found here and in other cancers, might disrupt the CHTF18-RFC interaction.

*MRE11A* was somatically mutated in 4.4% (2 of 45) of serous ECs. No *MRE11A* mutations were observed among clear cell or endometrioid tumors. MRE11A possesses both endonuclease activity and 3′–5′ exonuclease activity and, as a component the MRE11A-RAD50-NBS1 (MRN) complex, it plays an essential role in the cellular response to double strand breaks (reviewed in [59]). In mammalian cells, the MRN complex is also required for ATR-mediated phosphorylation of the SMC1 subunit of cohesin [60], and siRNA depletion of *MRE11A* in human cells results in cohesion defects [37]. The MRE11A^D131N^ somatic mutant, which we uncovered in a serous EC, occurs at a highly evolutionarily conserved residue in the third phosphoesterase motif within the nuclease domain [61] and is predicted to impact protein function (**Figure 1** **, and** **Table 2**). The MRE11A^D692Y^ mutant, in the DNA binding domain, is also predicted to be functionally significant (**Table 2**). Although intronic somatic mutations in *MRE11A* have been reported in microsatellite unstable endometrial cancers [62], [63], [64], to our knowledge, the present study is the first report of somatic mutations of *MRE11A* in microsatellite stable endometrial tumors (**Table 2**). Of note, the MRE11A^D131N^ variant, which was somatic in our study, has also been observed as a rare population variant (TMP_ESP_11_94212851) in the NHLBI Exome Sequencing Project (URL: http://evs.gs.washington.edu/EVS/), with a minor allele frequency of 0.0233% in the EuropeanAmerican population.

**Figure 2:**
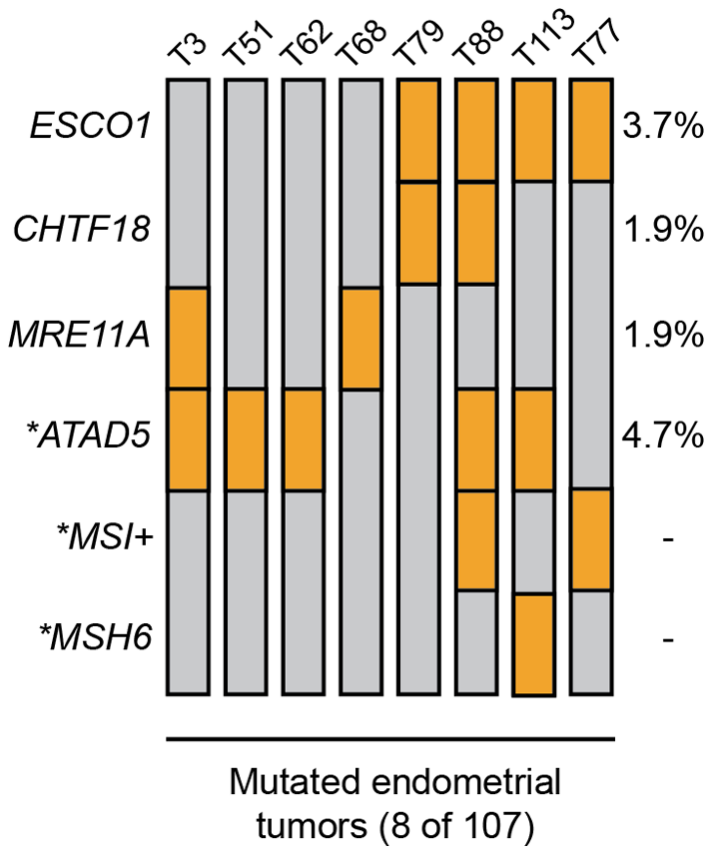
Oncoprint displaying nonsynonymous somatic mutations in *ESCO1*, *CHTF18*, *MRE11A*, and *ATAD5* in eight primary endometrial cancers. Individual tumors (T) are indicated by vertical gray bars. Tumors consist of NEECs (T3, T51, T62, T68, T77, T79, T113) and an EEC (T88). Genes (left) and nonsynonymous somatic mutations (orange boxes) are indicated. *ESCO1*, *CHTF18*, and *MRE11A* were analyzed in this study; *ATAD5 mutations, *MSH6* mutations, and microsatellite instability (MSI) have previously been described elsewhere [44], [52].

The mutual exclusivity or co-occurrence of somatic mutations in two or more genes can indicate functional redundancy or functional synergy, respectively. To determine the pattern of somatic mutations within cohesion genes in endometrial cancer, we combined the results of the present study with our previous analysis of the *ATAD5 (hELG1)* gene in this same cohort of ECs [44]. Although the number of mutated cases is small, we observed that somatic mutations in *ESCO1* and *ATAD5* tended to co-occur in endometrial cancer (*P* = 0.0102, two-tailed Fisher's exact test), as did somatic mutations in *ESCO1* and *CHTF18* (*P* = 0.0011) (**Figure 2** **, and** **Table 3**). These observations raise the possibility that there might be functional synergy between *ESCO1* and *ATAD5* mutants, and between *ESCO1* and *CHTF18* mutants, in endometrial cancer. In this regard, it is noteworthy that somatic mutations in *ESCO1* and *ATAD5* tend to also co-occur in colorectal tumors (*P* = 0.000001) (**Figure S7**), based on an analysis of the publically available mutation data generated by The Cancer Genome Atlas [http://cbio.mskcc.org/cancergenomics/]. An alternative, but not mutually exclusive, possibility is that the co-occurring mutations of cohesion genes in endometrial cancer may reflect an underlying hypermutable phenotype. We previously evaluated the cohort of 107 tumors in this study for microsatellite instability and *MSH6* mutations [44], [52], both of which can give rise to hypermutability due to defective mismatch repair (MMR). Although three of the tumors with cohesion gene mutations in this study were either MSI-unstable or *MSH6*-mutated (**Figure 2**), we observed no statistically significant association between mutations in sister chromatid cohesion genes and defects in mismatch repair (**Table S4 and Table S5**).

**Table 3:** Co-occurrence of *ESCO1* mutations with *CHTF18* or *ATAD5* mutations in EC.

Mutation Status	No. of *ESCO1-*mutated Cases (%)	*P-*value§
*CHTF18-*mutated (n = 2)	2 (100%)	*P* = 0.0011
*CHTF18-*nonmutated (n = 105)	2 (1.90%)	
*ATAD5-*mutated (n = 5)	2 (40%)	*P* = 0.0102
*ATAD5-*nonmutated (n = 102)	2 (1.96%)	

§ Two-tailed Fisher's exact test.

In summary, we have identified rare, nonsynonymous, somatic mutations within *ESCO1*, *CHTF18*, and *MRE11A* in a subset of primary endometrial tumors. Future studies will be required to determine whether these mutations are driver events that contribute to the pathogenesis of endometrial cancer.

## Supporting Information

Figure S1 **RT-PCR analysis of 21 candidate human chromosomal instability genes in 7 human endometrial cancer cell lines.** Gel electrophoresis of RT-PCR products confirms the expression of the 21 candidate chromosome instability genes in serous and endometrioid endometrial cancer cell lines. Positive and negative (water) PCR controls are shown. *ACTB* and *GAPDH* served as positive control genes.
(TIF)

Figure S2 **Sequence chromatograms showing somatic mutations in** ***ESCO1*** **,** ***CHTF18*** **, and** ***MRE11A*** **in endometrial tumor DNAs, compared to the matched normal DNAs.**
(TIF)

Figure S3 Oncoprints displaying the distribution of somatic mutations in serous endometrial tumors as reported in this study (*) and elsewhere [**44**] **,** [**52**] **,** [**53**] **,** [**54**] **.** Each blue bar represents an individual tumor (T). Nonsynonymous somatic mutations and MSI+ are indicated by the red bars. For *MSH6*, germline variants of unknown functional significance are displayed by orange bars. The observed frequency (%) of mutated cases, for each gene, is shown on the right.
(TIF)

Figure S4 Oncoprints displaying the distribution of somatic mutations in clear cell endometrial tumors as reported in this study (*) and elsewhere [**44**] **,** [**52**] **,** [**53**] **,** [**54**] **.** Each blue bar represents an individual tumor (T). Nonsynonymous somatic mutations and MSI+ are indicated by the red bars. For *MSH6*, a germline variant of unknown functional significance is displayed by the orange bar. The observed frequency (%) of mutated cases, for each gene, is shown on the right.
(TIF)

Figure S5 Oncoprints displaying the distribution of somatic mutations in endometrioid endometrial tumors as reported in this study (*) and elsewhere [**44**] **,** [**52**] **,** [**53**] **,** [**54**] **.** Each blue bar represents an individual tumor (T). Nonsynonymous somatic mutations and MSI+ are indicated by the red bars. For *MSH6*, germline variants of unknown functional significance are displayed by orange bars. The observed frequency (%) of mutated cases, for each gene, is shown on the right.
(TIF)

Figure S6 **Immunoblots showing expression levels of the MRE11A, CHTF18 and ESCO1 proteins among a panel of 7 human endometrial cancer cell lines.** Tubulin was used as a control for protein loading.
(TIF)

Figure S7 **Oncoprint displaying patterns of somatic mutations in** ***ESCO1*** **,** ***CHTF18*** **,** ***MRE11A*** **, and** ***ATAD5*** **in colorectal cancer, as reported by The Cancer Genome Atlas (TCGA).** (Upper panel) Individual colorectal tumors are indicated by vertical gray bars. Genes (left) and nonsynonymous somatic mutations (orange bars) are indicated. (Lower panel) In colorectal cancers, mutations in *ATAD5* and *ESCO1* showed a strong tendency towards co-occurrence; mutations in *MRE11A* and *ESCO1*, and in *ATAD5* and *MRE11A* showed a tendency towards co-occurrence. The data were derived from 224 sequenced samples; the TCGA data were accessed, and the mutual exclusivity calculated via the cBio Cancer Genomics Portal (http://www.cbioportal.org/public-portal/).
(TIF)

Table S1 **RT-PCR primers used to assess the expression of 21 candidate human chromosomal instability genes.**
(XLSX)

Table S2 **PCR primers used to amplify 21 candidate human chromosomal instability genes within the discovery screen.**
(DOC)

Table S3 **PCR primers used to amplify and sequence** ***CHTF18, ESCO1,*** **and** ***MRE11A*** **within the validation screen.**
(DOC)

Table S4 **Status of microsatellite instability,** ***MSH6*** **,** ***ESCO1*** **,** ***CHTF18*** **,** ***MRE11A*** **, and** ***ATAD5*** **for the 107 endometrial tumors in this study.**
(XLSX)

Table S5 **Frequency of somatic mutations in the** ***ESCO1*** **,** ***CHTF18*** **,** ***MRE11A*** **, and** ***ATAD5*** **cohesion genes in 105 endometrial tumors, according to microsatellite instability and** ***MSH6*** **status.**
(XLSX)

## References

[1] American Cancer Society (2013) Cancer Facts and Figures. American Cancer Society 1: 1–60.

[2] Sherman ME (2000) Theories of endometrial carcinogenesis: a multidisciplinary approach. Mod Pathol 13: 295–308. 10757340 10.1038/modpathol.3880051

[3] Hecht JL, Mutter GL (2006) Molecular and pathologic aspects of endometrial carcinogenesis. J Clin Oncol 24: 4783–4791. 17028294 10.1200/JCO.2006.06.7173

[4] Ries LAG, Young JL, Keel GE, Eisner MP, Lin YD, et al.. (2007) SEER Survival Monograph: Cancer Survival Among Adults: U.S. SEER Program, 1988–2001. Patient and Tumor Characteristics. National Cancer Institute, SEER Program, NIH Pub No 07–6215, Bethesda, MD, 2007.

[5] Hamilton CA, Cheung MK, Osann K, Chen L, Teng NN, et al (2006) Uterine papillary serous and clear cell carcinomas predict for poorer survival compared to grade 3 endometrioid corpus cancers. Br J Cancer 94: 642–646. 16495918 10.1038/sj.bjc.6603012 PMC2361201

[6] Burks RT, Kessis TD, Cho KR, Hedrick L (1994) Microsatellite instability in endometrial carcinoma. Oncogene 9: 1163–1166. 8134118

[7] Duggan BD, Felix JC, Muderspach LI, Tourgeman D, Zheng J, et al (1994) Microsatellite instability in sporadic endometrial carcinoma. J Natl Cancer Inst 86: 1216–1221. 8040889 10.1093/jnci/86.16.1216

[8] Kobayashi K, Sagae S, Kudo R, Saito H, Koi S, et al (1995) Microsatellite instability in endometrial carcinomas: frequent replication errors in tumors of early onset and/or of poorly differentiated type. Genes Chromosomes Cancer 14: 128–132. 8527394 10.1002/gcc.2870140207

[9] Esteller M, Catasus L, Matias-Guiu X, Mutter GL, Prat J, et al (1999) hMLH1 promoter hypermethylation is an early event in human endometrial tumorigenesis. Am J Pathol 155: 1767–1772. 10550333 10.1016/S0002-9440(10)65492-2 PMC1866976

[10] Goodfellow PJ, Buttin BM, Herzog TJ, Rader JS, Gibb RK, et al (2003) Prevalence of defective DNA mismatch repair and MSH6 mutation in an unselected series of endometrial cancers. Proc Natl Acad Sci U S A 100: 5908–5913. 12732731 10.1073/pnas.1030231100 PMC156300

[11] Micci F, Teixeira MR, Haugom L, Kristensen G, Abeler VM, et al (2004) Genomic aberrations in carcinomas of the uterine corpus. Genes Chromosomes Cancer 40: 229–246. 15139002 10.1002/gcc.20038

[12] Konski AA, Domenico D, Irving D, Tyrkus M, Neisler J, et al (1996) Clinicopathologic correlation of DNA flow cytometric content analysis (DFCA), surgical staging, and estrogen/progesterone receptor status in endometrial adenocarcinoma. Am J Clin Oncol 19: 164–168. 8610642 10.1097/00000421-199604000-00015

[13] Newbury R, Schuerch C, Goodspeed N, Fanning J, Glidewell O, et al (1990) DNA content as a prognostic factor in endometrial carcinoma. Obstet Gynecol 76: 251–257. 2371030

[14] Pradhan M, Abeler VM, Danielsen HE, Trope CG, Risberg BA (2006) Image cytometry DNA ploidy correlates with histological subtypes in endometrial carcinomas. Mod Pathol 19: 1227–1235. 16729014 10.1038/modpathol.3800641

[15] Prat J, Oliva E, Lerma E, Vaquero M, Matias-Guiu X (1994) Uterine papillary serous adenocarcinoma. A 10-case study of p53 and c-erbB-2 expression and DNA content. Cancer 74: 1778–1783. 7915964 10.1002/1097-0142(19940915)74:6<1778::aid-cncr2820740621>3.0.co;2-5

[16] Rosenberg P, Wingren S, Simonsen E, Stal O, Risberg B, et al (1989) Flow cytometric measurements of DNA index and S-phase on paraffin-embedded early stage endometrial cancer: an important prognostic indicator. Gynecol Oncol 35: 50–54. 2792902 10.1016/0090-8258(89)90010-3

[17] Pere H, Tapper J, Wahlstrom T, Knuutila S, Butzow R (1998) Distinct chromosomal imbalances in uterine serous and endometrioid carcinomas. Cancer Res 58: 892–895. 9500445

[18] Kunkel TA, Erie DA (2005) DNA mismatch repair. Annu Rev Biochem 74: 681–710. 15952900 10.1146/annurev.biochem.74.082803.133243

[19] O'Hara AJ, Bell DW (2012) The genomics and genetics of endometrial cancer. Adv Genomics Genet 2012: 33–47. 22888282 10.2147/AGG.S28953 PMC3415201

[20] Thompson SL, Compton DA (2010) Proliferation of aneuploid human cells is limited by a p53-dependent mechanism. J Cell Biol 188: 369–381. 20123995 10.1083/jcb.200905057 PMC2819684

[21] Mayer ML, Gygi SP, Aebersold R, Hieter P (2001) Identification of RFC(Ctf18p, Ctf8p, Dcc1p): an alternative RFC complex required for sister chromatid cohesion in S. cerevisiae. Mol Cell 7: 959–970. 11389843 10.1016/s1097-2765(01)00254-4

[22] Tanaka K, Yonekawa T, Kawasaki Y, Kai M, Furuya K, et al (2000) Fission yeast Eso1p is required for establishing sister chromatid cohesion during S phase. Mol Cell Biol 20: 3459–3469. 10779336 10.1128/mcb.20.10.3459-3469.2000 PMC85639

[23] Guacci V, Koshland D, Strunnikov A (1997) A direct link between sister chromatid cohesion and chromosome condensation revealed through the analysis of MCD1 in S. cerevisiae. Cell 91: 47–57. 9335334 10.1016/s0092-8674(01)80008-8 PMC2670185

[24] Michaelis C, Ciosk R, Nasmyth K (1997) Cohesins: chromosomal proteins that prevent premature separation of sister chromatids. Cell 91: 35–45. 9335333 10.1016/s0092-8674(01)80007-6

[25] Ciosk R, Shirayama M, Shevchenko A, Tanaka T, Toth A, et al (2000) Cohesin's binding to chromosomes depends on a separate complex consisting of Scc2 and Scc4 proteins. Mol Cell 5: 243–254. 10882066 10.1016/s1097-2765(00)80420-7

[26] Zhang J, Shi X, Li Y, Kim BJ, Jia J, et al (2008) Acetylation of Smc3 by Eco1 is required for S phase sister chromatid cohesion in both human and yeast. Mol Cell 31: 143–151. 18614053 10.1016/j.molcel.2008.06.006

[27] Rolef Ben-Shahar T, Heeger S, Lehane C, East P, Flynn H, et al (2008) Eco1-dependent cohesin acetylation during establishment of sister chromatid cohesion. Science 321: 563–566. 18653893 10.1126/science.1157774

[28] Toth A, Ciosk R, Uhlmann F, Galova M, Schleiffer A, et al (1999) Yeast cohesin complex requires a conserved protein, Eco1p(Ctf7), to establish cohesion between sister chromatids during DNA replication. Genes Dev 13: 320–333. 9990856 10.1101/gad.13.3.320 PMC316435

[29] Skibbens RV (2004) Chl1p, a DNA helicase-like protein in budding yeast, functions in sister-chromatid cohesion. Genetics 166: 33–42. 15020404 10.1534/genetics.166.1.33 PMC1470669

[30] Sutani T, Kawaguchi T, Kanno R, Itoh T, Shirahige K (2009) Budding yeast Wpl1(Rad61)-Pds5 complex counteracts sister chromatid cohesion-establishing reaction. Curr Biol 19: 492–497. 19268589 10.1016/j.cub.2009.01.062

[31] Maradeo ME, Skibbens RV (2010) Replication factor C complexes play unique pro- and anti-establishment roles in sister chromatid cohesion. PLoS One 5: e15381. 21060875 10.1371/journal.pone.0015381 PMC2965161

[32] Nasmyth K (2011) Cohesin: a catenase with separate entry and exit gates? Nat Cell Biol 13: 1170–1177. 21968990 10.1038/ncb2349

[33] Hou F, Zou H (2005) Two human orthologues of Eco1/Ctf7 acetyltransferases are both required for proper sister-chromatid cohesion. Mol Biol Cell 16: 3908–3918. 15958495 10.1091/mbc.E04-12-1063 PMC1182326

[34] Terret ME, Sherwood R, Rahman S, Qin J, Jallepalli PV (2009) Cohesin acetylation speeds the replication fork. Nature 462: 231–234. 19907496 10.1038/nature08550 PMC2777716

[35] Parish JL, Rosa J, Wang X, Lahti JM, Doxsey SJ, et al (2006) The DNA helicase ChlR1 is required for sister chromatid cohesion in mammalian cells. J Cell Sci 119: 4857–4865. 17105772 10.1242/jcs.03262

[36] Farina A, Shin JH, Kim DH, Bermudez VP, Kelman Z, et al (2008) Studies with the human cohesin establishment factor, ChlR1. Association of ChlR1 with Ctf18-RFC and Fen1. J Biol Chem 283: 20925–20936. 18499658 10.1074/jbc.M802696200 PMC2475708

[37] Barber TD, McManus K, Yuen KW, Reis M, Parmigiani G, et al (2008) Chromatid cohesion defects may underlie chromosome instability in human colorectal cancers. Proc Natl Acad Sci U S A 105: 3443–3448. 18299561 10.1073/pnas.0712384105 PMC2265152

[38] Cahill DP, Lengauer C, Yu J, Riggins GJ, Willson JK, et al (1998) Mutations of mitotic checkpoint genes in human cancers. Nature 392: 300–303. 9521327 10.1038/32688

[39] Rajagopalan H, Jallepalli PV, Rago C, Velculescu VE, Kinzler KW, et al (2004) Inactivation of hCDC4 can cause chromosomal instability. Nature 428: 77–81. 14999283 10.1038/nature02313

[40] Wang Z, Cummins JM, Shen D, Cahill DP, Jallepalli PV, et al (2004) Three classes of genes mutated in colorectal cancers with chromosomal instability. Cancer Res 64: 2998–3001. 15126332 10.1158/0008-5472.can-04-0587

[41] Solomon DA, Kim T, Diaz-Martinez LA, Fair J, Elkahloun AG, et al (2011) Mutational inactivation of STAG2 causes aneuploidy in human cancer. Science 333: 1039–1043. 21852505 10.1126/science.1203619 PMC3374335

[42] Welch JS, Ley TJ, Link DC, Miller CA, Larson DE, et al (2012) The origin and evolution of mutations in acute myeloid leukemia. Cell 150: 264–278. 22817890 10.1016/j.cell.2012.06.023 PMC3407563

[43] Rocquain J, Gelsi-Boyer V, Adelaide J, Murati A, Carbuccia N, et al (2010) Alteration of cohesin genes in myeloid diseases. Am J Hematol 85: 717–719. 20687102 10.1002/ajh.21798

[44] Bell DW, Sikdar N, Lee KY, Price JC, Chatterjee R, et al (2011) Predisposition to cancer caused by genetic and functional defects of mammalian Atad5. PLoS Genetics 7: e1002245. 21901109 10.1371/journal.pgen.1002245 PMC3161924

[45] Parnas O, Zipin-Roitman A, Mazor Y, Liefshitz B, Ben-Aroya S, et al (2009) The ELG1 clamp loader plays a role in sister chromatid cohesion. PLoS One 4: e5497. 19430531 10.1371/journal.pone.0005497 PMC2676507

[46] Maradeo ME, Skibbens RV (2009) The Elg1-RFC clamp-loading complex performs a role in sister chromatid cohesion. PLoS One 4: e4707. 19262753 10.1371/journal.pone.0004707 PMC2650802

[47] Chines P, Swift A, Bonnycastle LL, Erdos MR, Mullikin J, et al (2005) PrimerTile: designing overlapping PCR primers for resequencing. Am J Hum Genet 77: 1257.

[48] Ewing B, Green P (1998) Base-calling of automated sequencer traces using phred. II. Error probabilities. Genome Res 8: 186–194. 9521922

[49] Ewing B, Hillier L, Wendl MC, Green P (1998) Base-calling of automated sequencer traces using phred. I. Accuracy assessment. Genome Res 8: 175–185. 9521921 10.1101/gr.8.3.175

[50] Gordon D, Abajian C, Green P (1998) Consed: a graphical tool for sequence finishing. Genome Res 8: 195–202. 9521923 10.1101/gr.8.3.195

[51] Bhangale TR, Stephens M, Nickerson DA (2006) Automating resequencing-based detection of insertion-deletion polymorphisms. Nat Genet 38: 1457–1462. 17115056 10.1038/ng1925

[52] Le Gallo M, O'Hara AJ, Rudd ML, Urick ME, Hansen NF, et al (2012) Exome sequencing of serous endometrial tumors identifies recurrent somatic mutations in chromatin-remodeling and ubiquitin ligase complex genes. Nat Genet 44: 1310–1315. 23104009 10.1038/ng.2455 PMC3515204

[53] Urick ME, Rudd ML, Godwin AK, Sgroi D, Merino M, et al (2011) PIK3R1 (p85alpha) is somatically mutated at high frequency in primary endometrial cancer. Cancer Res 71: 4061–4067. 21478295 10.1158/0008-5472.CAN-11-0549 PMC3117071

[54] Rudd ML, Price JC, Fogoros S, Godwin AK, Sgroi DC, et al (2011) A unique spectrum of somatic PIK3CA (p110alpha) mutations within primary endometrial carcinomas. Clin Cancer Res 17: 1331–1340. 21266528 10.1158/1078-0432.CCR-10-0540 PMC3060282

[55] Shiomi Y, Masutani C, Hanaoka F, Kimura H, Tsurimoto T (2007) A second proliferating cell nuclear antigen loader complex, Ctf18-replication factor C, stimulates DNA polymerase eta activity. J Biol Chem 282: 20906–20914. 17545166 10.1074/jbc.M610102200

[56] Ogi T, Limsirichaikul S, Overmeer RM, Volker M, Takenaka K, et al (2010) Three DNA polymerases, recruited by different mechanisms, carry out NER repair synthesis in human cells. Mol Cell 37: 714–727. 20227374 10.1016/j.molcel.2010.02.009

[57] Murakami T, Takano R, Takeo S, Taniguchi R, Ogawa K, et al (2010) Stable interaction between the human proliferating cell nuclear antigen loader complex Ctf18-replication factor C (RFC) and DNA polymerase {epsilon} is mediated by the cohesion-specific subunits, Ctf18, Dcc1, and Ctf8. J Biol Chem 285: 34608–34615. 20826785 10.1074/jbc.M110.166710 PMC2966076

[58] Forbes SA, Bhamra G, Bamford S, Dawson E, Kok C, et al.. (2008) The Catalogue of Somatic Mutations in Cancer (COSMIC). Curr Protoc Hum Genet Chapter 10: Unit 10 11. 10.1002/0471142905.hg1011s57 PMC2705836 18428421

[59] Stracker TH, Petrini JH (2011) The MRE11 complex: starting from the ends. Nat Rev Mol Cell Biol 12: 90–103. 21252998 10.1038/nrm3047 PMC3905242

[60] Zhong H, Bryson A, Eckersdorff M, Ferguson DO (2005) Rad50 depletion impacts upon ATR-dependent DNA damage responses. Hum Mol Genet 14: 2685–2693. 16087684 10.1093/hmg/ddi302

[61] Moreau S, Ferguson JR, Symington LS (1999) The nuclease activity of Mre11 is required for meiosis but not for mating type switching, end joining, or telomere maintenance. Mol Cell Bio 19: 556–566. 9858579 10.1128/mcb.19.1.556 PMC83913

[62] Giannini G, Rinaldi C, Ristori E, Ambrosini MI, Cerignoli F, et al (2004) Mutations of an intronic repeat induce impaired MRE11 expression in primary human cancer with microsatellite instability. Oncogene 23: 2640–2647. 15048091 10.1038/sj.onc.1207409

[63] Bilbao C, Ramirez R, Rodriguez G, Falcon O, Leon L, et al (2010) Double strand break repair components are frequent targets of microsatellite instability in endometrial cancer. Eur J Cancer 46: 2821–2827. 20638839 10.1016/j.ejca.2010.06.116

[64] Giannini G, Ristori E, Cerignoli F, Rinaldi C, Zani M, et al (2002) Human MRE11 is inactivated in mismatch repair-deficient cancers. EMBO Rep 3: 248–254. 11850399 10.1093/embo-reports/kvf044 PMC1084012

[65] Berkowitz KM, Kaestner KH, Jongens TA (2008) Germline expression of mammalian CTF18, an evolutionarily conserved protein required for germ cell proliferation in the fly and sister chromatid cohesion in yeast. Mol Hum Reprod 14: 143–150. 18250106 10.1093/molehr/gan005

[66] Park YB, Chae J, Kim YC, Cho Y (2011) Crystal structure of human Mre11: understanding tumorigenic mutations. Structure 19: 1591–1602. 22078559 10.1016/j.str.2011.09.010

[67] Williams GJ, Williams RS, Williams JS, Moncalian G, Arvai AS, et al (2011) ABC ATPase signature helices in Rad50 link nucleotide state to Mre11 interface for DNA repair. Nat Struct Mol Biol 18: 423–431. 21441914 10.1038/nsmb.2038 PMC3118400

