# Supplementary figures and images for: Sequencing of Candidate Chromosome Instability Genes in Endometrial Cancers Reveals Somatic Mutations in ESCO1, CHTF18, and MRE11A
Source: PLoS One. 2013 Jun 3;8(6):e63313. doi: 10.1371/journal.pone.0063313 (PMC3670891; doi:10.1371/journal.pone.0063313)

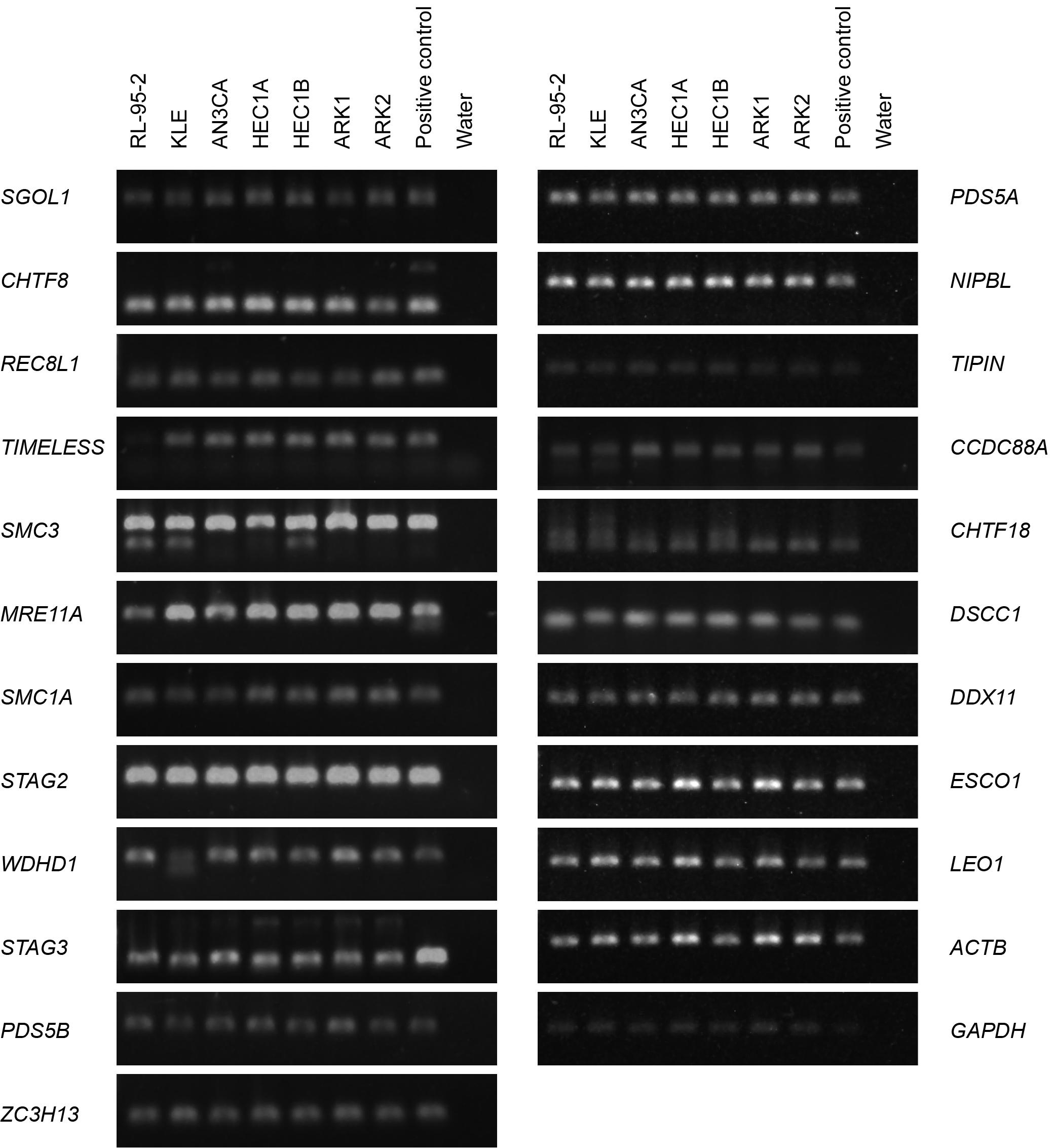

Supplement: Figure S1 — RT-PCR analysis of 21 candidate human chromosomal instability genes in 7 human endometrial cancer cell lines. Gel electrophoresis of RT-PCR products confirms the expression of the 21 candidate chromosome instability genes in serous and endometrioid endometrial cancer cell lines. Positive and negative (water) PCR controls are shown. ACTB and GAPDH served as positive control genes. (TIF) [file pone.0063313.s001.tif]

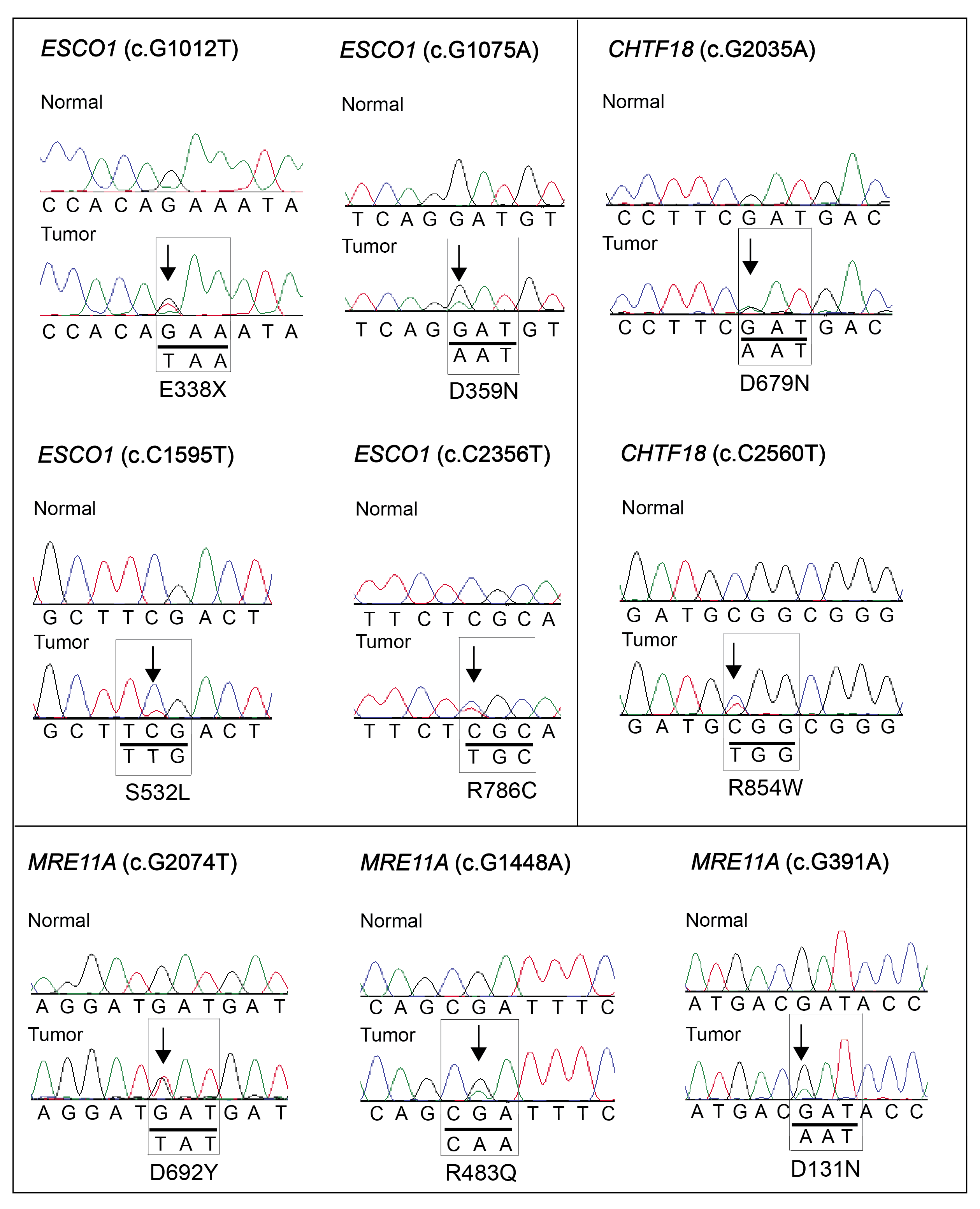

Supplement: Figure S2 — Sequence chromatograms showing somatic mutations in ESCO1 , CHTF18 , and MRE11A in endometrial tumor DNAs, compared to the matched normal DNAs. (TIF) [file pone.0063313.s002.tif]

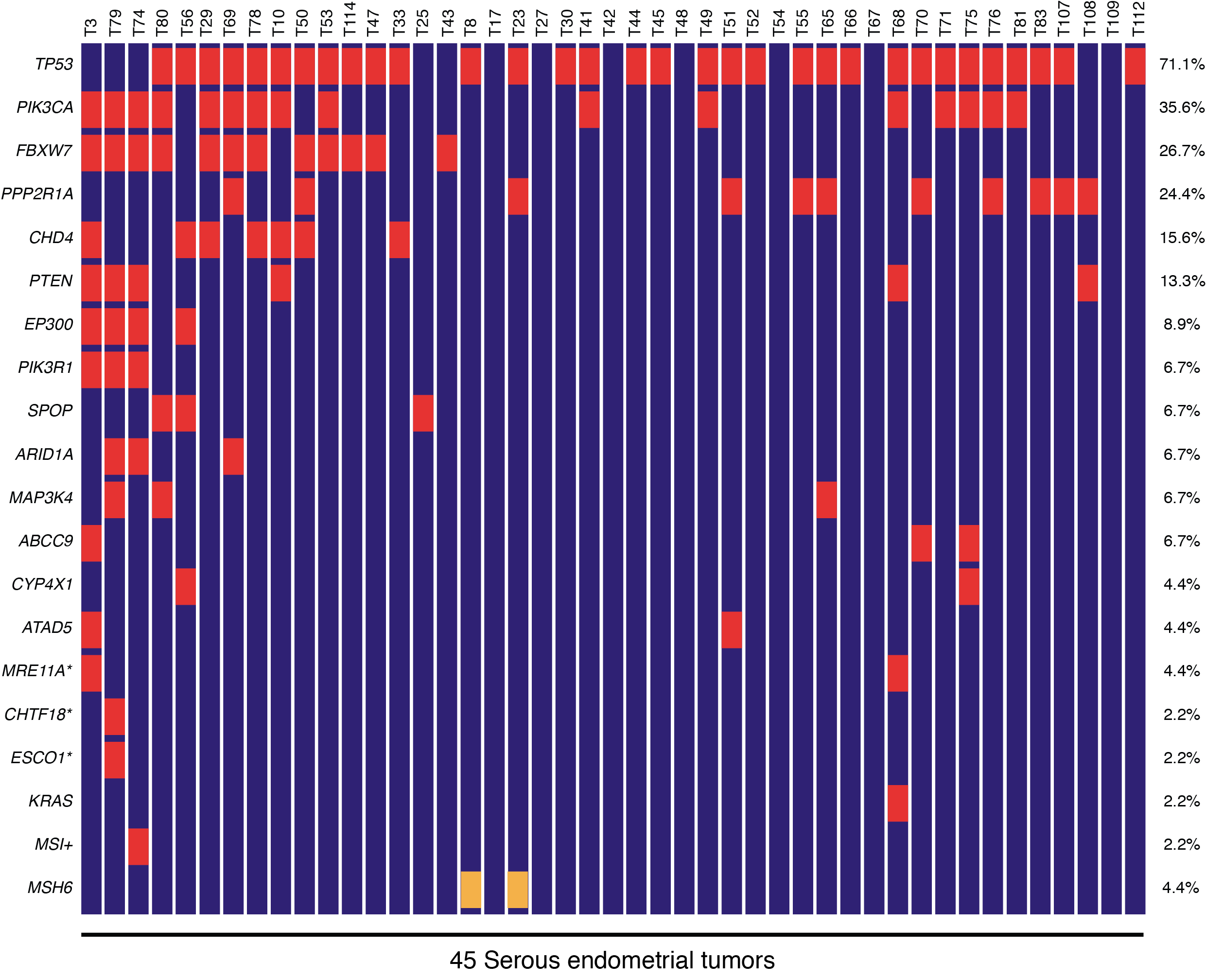

Supplement: Figure S3 — Oncoprints displaying the distribution of somatic mutations in serous endometrial tumors as reported in this study (*) and elsewhere [44] , [52] , [53] , [54] . Each blue bar represents an individual tumor (T). Nonsynonymous somatic mutations and MSI+ are indicated by the red bars. For MSH6, germline variants of unknown functional significance are displayed by orange bars. The observed frequency (%) of mutated cases, for each gene, is shown on the right. (TIF) [file pone.0063313.s003.tif]

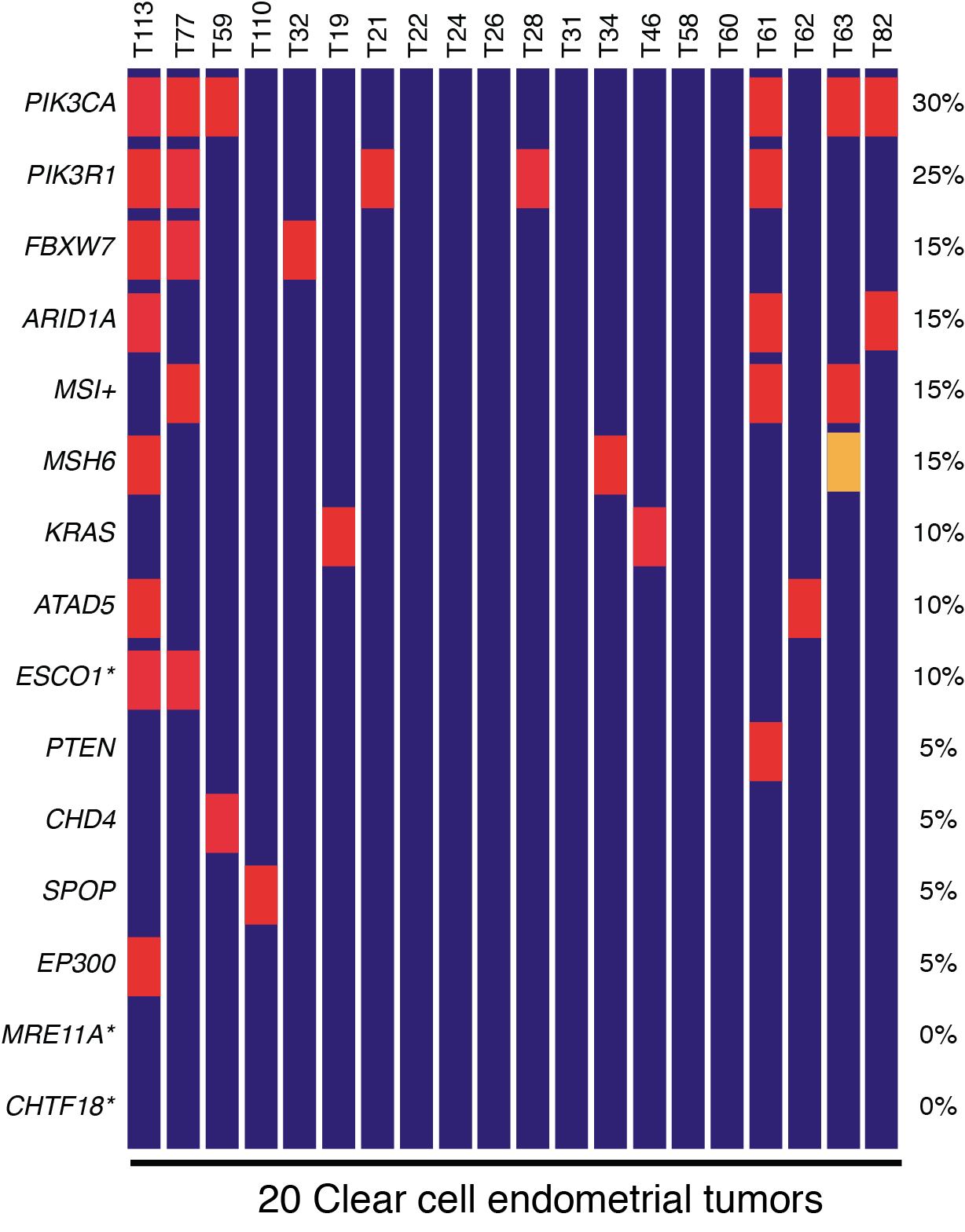

Supplement: Figure S4 — Oncoprints displaying the distribution of somatic mutations in clear cell endometrial tumors as reported in this study (*) and elsewhere [44] , [52] , [53] , [54] . Each blue bar represents an individual tumor (T). Nonsynonymous somatic mutations and MSI+ are indicated by the red bars. For MSH6, a germline variant of unknown functional significance is displayed by the orange bar. The observed frequency (%) of mutated cases, for each gene, is shown on the right. (TIF) [file pone.0063313.s004.tif]

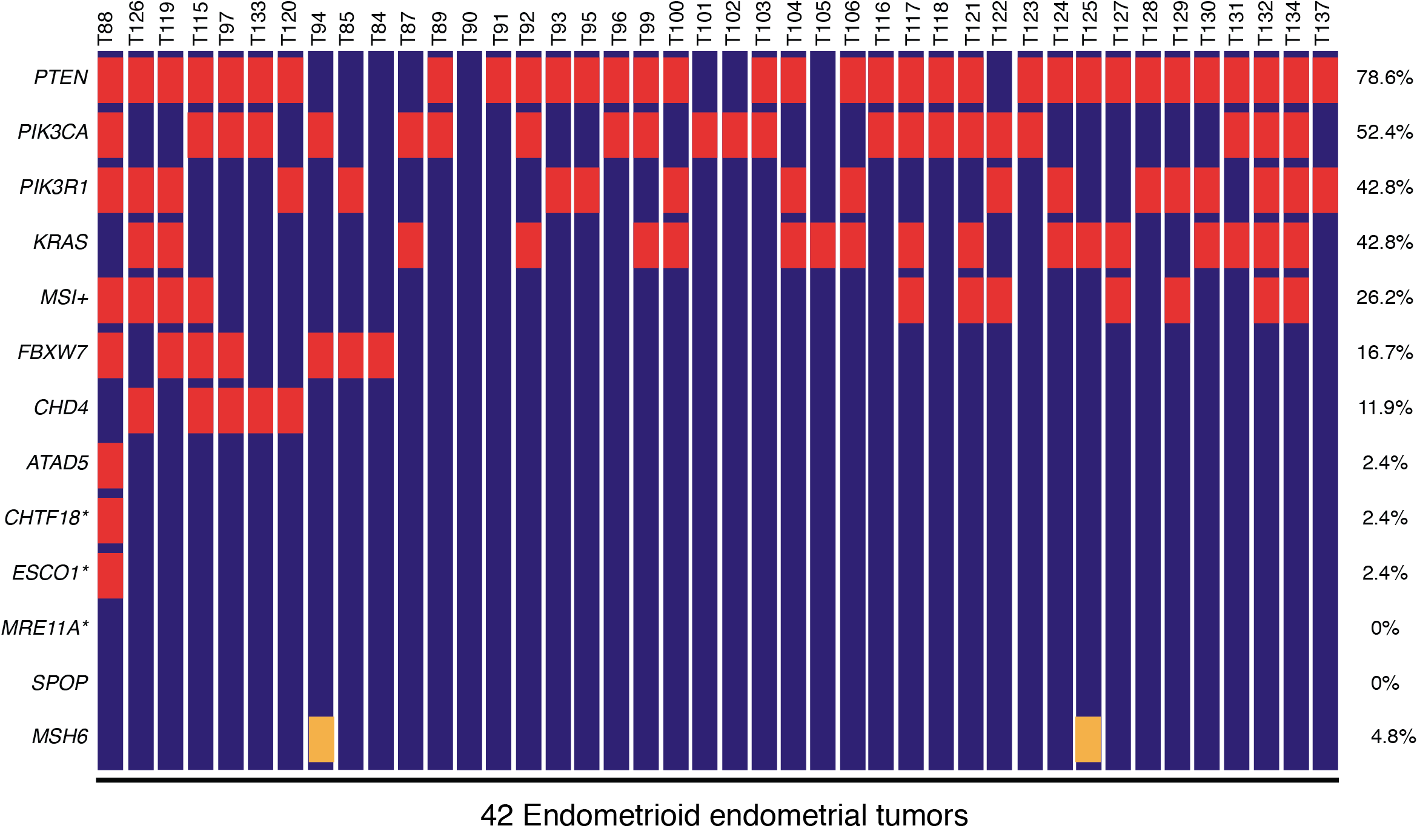

Supplement: Figure S5 — Oncoprints displaying the distribution of somatic mutations in endometrioid endometrial tumors as reported in this study (*) and elsewhere [44] , [52] , [53] , [54] . Each blue bar represents an individual tumor (T). Nonsynonymous somatic mutations and MSI+ are indicated by the red bars. For MSH6, germline variants of unknown functional significance are displayed by orange bars. The observed frequency (%) of mutated cases, for each gene, is shown on the right. (TIF) [file pone.0063313.s005.tif]

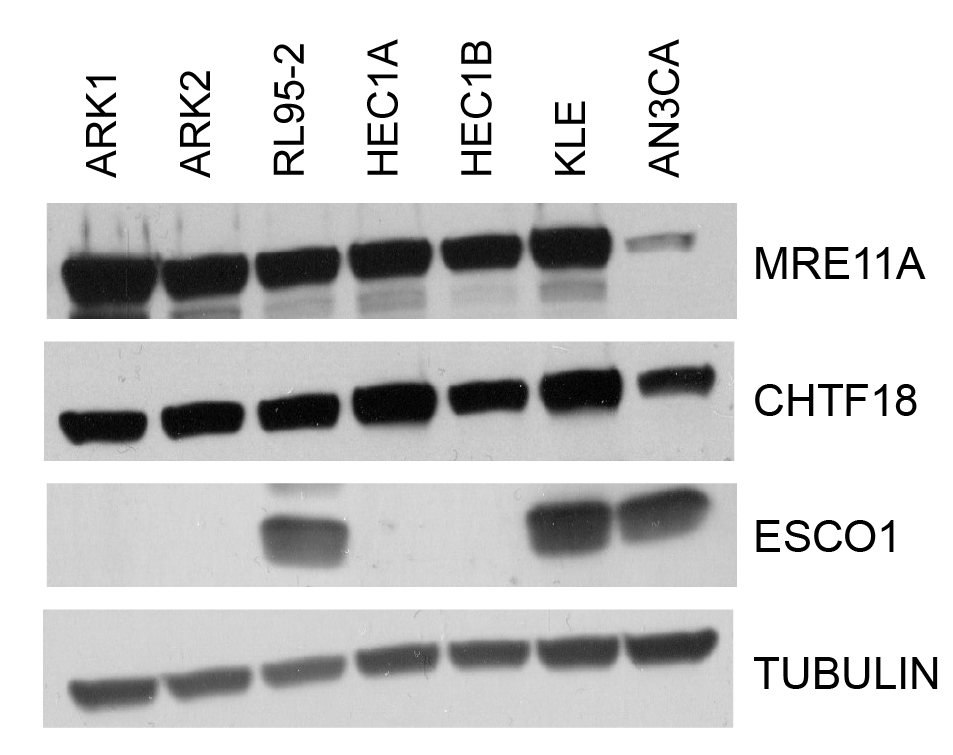

Supplement: Figure S6 — Immunoblots showing expression levels of the MRE11A, CHTF18 and ESCO1 proteins among a panel of 7 human endometrial cancer cell lines. Tubulin was used as a control for protein loading. (TIF) [file pone.0063313.s006.tif]

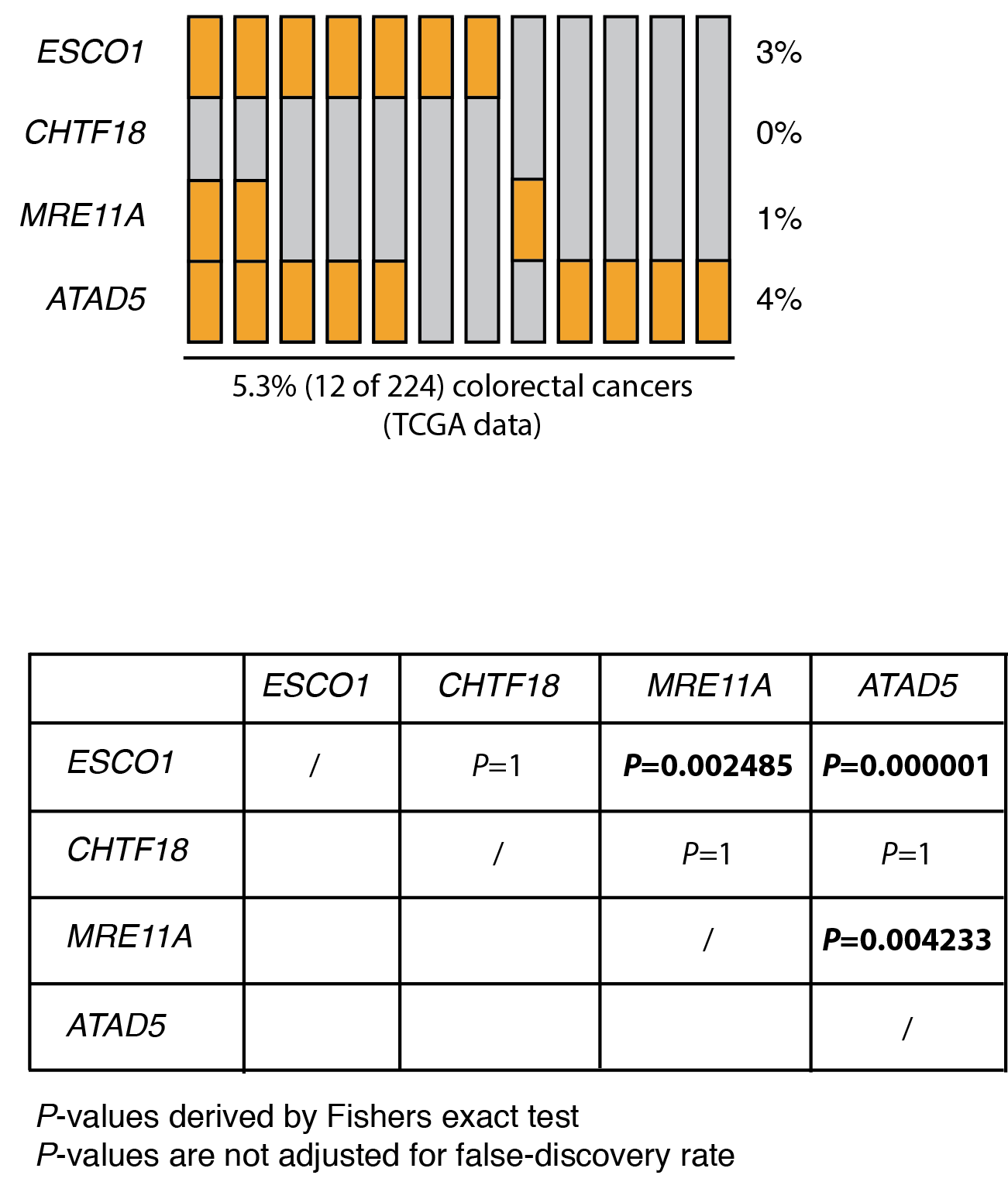

Supplement: Figure S7 — Oncoprint displaying patterns of somatic mutations in ESCO1 , CHTF18 , MRE11A , and ATAD5 in colorectal cancer, as reported by The Cancer Genome Atlas (TCGA). (Upper panel) Individual colorectal tumors are indicated by vertical gray bars. Genes (left) and nonsynonymous somatic mutations (orange bars) are indicated. (Lower panel) In colorectal cancers, mutations in ATAD5 and ESCO1 showed a strong tendency towards co-occurrence; mutations in MRE11A and ESCO1, and in ATAD5 and MRE11A showed a tendency towards co-occurrence. The data were derived from 224 sequenced samples; the TCGA data were accessed, and the mutual exclusivity calculated via the cBio Cancer Genomics Portal (http://www.cbioportal.org/public-portal/). (TIF) [file pone.0063313.s007.tif]
