# Supplementary material for: Sequencing of Candidate Chromosome Instability Genes in Endometrial Cancers Reveals Somatic Mutations in ESCO1, CHTF18, and MRE11A
Source: PLoS One. 2013 Jun 3;8(6):e63313. doi: 10.1371/journal.pone.0063313 (PMC3670891; doi:10.1371/journal.pone.0063313)
Supplement: Table S2 — PCR primers used to amplify 21 candidate human chromosomal instability genes within the discovery screen. (DOC) [file pone.0063313.s009.doc]

| **Table S2. PCR primers used to amplify 21 candidate human chromosomal instability genes within the discovery screen** | | | |
| --- | --- | --- | --- |
| **Amplimer name** | **Region of Interest (Gene_Exon)** | **M13-Tailed forward primer (5'-3')** | **M13-Tailed reverse primer (5'-3')** |
| 1015416 | APRIN_exon_06 | TGTAAAACGACGGCCAGTTTTGTATGATGTGGGAGAGAGG | CAGGAAACAGCTATGACCTCTAAAAGATTAAAGCAGGATAAACCAT |
| 1011049 | APRIN_exon_02 | TGTAAAACGACGGCCAGTGAAATGTTCAATAGAGTGATTTTCATTA | CAGGAAACAGCTATGACCGAAGCCCTTTGACCTACTTTGA |
| 1011050 | APRIN_exon_03 | TGTAAAACGACGGCCAGTTCTTAAGGACATACTCCTTTTAGTCCA | CAGGAAACAGCTATGACCATTAAGTTTAGCAAAAGATTACTGTAGC |
| 1015415 | APRIN_exon_04 &5 | TGTAAAACGACGGCCAGTCTAGAGGATACAAAGAGCCCACA | CAGGAAACAGCTATGACCAGACAAATAATGCAATCCAAAAA |
| 1011051 | APRIN_exon_04;APRIN_exon_5 | TGTAAAACGACGGCCAGTTGATCAGCTCTCAGTCATTGC | CAGGAAACAGCTATGACCGGTTCTGTATAGCTGGGTGAAAA |
| 1011052 | APRIN_exon_04;APRIN_exon_5 | TGTAAAACGACGGCCAGTGAGGTAAGCAATATCTTGTATCTTGA | CAGGAAACAGCTATGACCTTTCCCTAATTTTTAACCAGTCTCA |
| 1011054 | APRIN_exon_07 | TGTAAAACGACGGCCAGTATCCTTACGTGGATGGAATCTT | CAGGAAACAGCTATGACCTCAATTTAAGCTCCAAAAACCTG |
| 1011055 | APRIN_exon_08 | TGTAAAACGACGGCCAGTTTCCATGTTTTATCTTTCATGTAGC | CAGGAAACAGCTATGACCAACTATTTTTATGGCCTCAGTGGT |
| 1015417 | APRIN_exon_09 | TGTAAAACGACGGCCAGTCAAAGAATGGGCAGCCTTAT | CAGGAAACAGCTATGACCTTTTCAGTTGGAATTAATCACTGC |
| 1015418 | APRIN_exon_10 | TGTAAAACGACGGCCAGTAATGTATATGGTTTATTCAGGTTCCTT | CAGGAAACAGCTATGACCTTTTTCCCCCTATGAAACTTAT |
| 1015419 | APRIN_exon_11 | TGTAAAACGACGGCCAGTTTTTGCTAGGGCTTATTTTCAAG | CAGGAAACAGCTATGACCGGATAAACATTGACTACCATTAAGAGC |
| / | APRIN_exon_12 | Not targeted | Not targeted |
| 1011060 | APRIN_exon_13 | TGTAAAACGACGGCCAGTATCCTGTTTTGGTCCCCTTCTA | CAGGAAACAGCTATGACCCCTACTGCTCTCCACCGTAATG |
| 1011048 | APRIN_exon_14 | TGTAAAACGACGGCCAGTTTAAGAAAAAGTATCCCCATTCTCTC | CAGGAAACAGCTATGACCTGATAAAAGATAAAATATGATACATCCA |
| 1011061 | APRIN_exon_15 | TGTAAAACGACGGCCAGTAGGGTGAGGGAAGACATGATTA | CAGGAAACAGCTATGACCTCCACTGAACAAAGGAGATGTT |
| 1015420 | APRIN_exon_16 | TGTAAAACGACGGCCAGTTTGTACGTAAGGATTTTAAGGCAAT | CAGGAAACAGCTATGACCTCATTTTAGAAGGACATACACCACA |
| 1015421 | APRIN_exon_17 | TGTAAAACGACGGCCAGTTTTTTCTGGGCTTGTTCAATTC | CAGGAAACAGCTATGACCACAGAGGAGTACACAATACACACAGA |
| 1011064 | APRIN_exon_18 | TGTAAAACGACGGCCAGTTTGCAGTAGTGTTAAAAACCAGCTA | CAGGAAACAGCTATGACCTGGCTCATTCTACAGCAGAGTT |
| 1015422 | APRIN_exon_19 | TGTAAAACGACGGCCAGTAGTGCTCTCAGATTTTGGTCAAC | CAGGAAACAGCTATGACCCCCACACTCATGCATCTTCTAA |
| 1011066 | APRIN_exon_20 | TGTAAAACGACGGCCAGTTGGTGACTTTTTCTGACCTTGT | CAGGAAACAGCTATGACCCTGATTTACAAAACCTTACACTTCAAT |
| 1011067 | APRIN_exon_21 | TGTAAAACGACGGCCAGTCTGATGCCTTCTGGACATAGAT | CAGGAAACAGCTATGACCCCCCTGTAAATCTGAAATAGATAGC |
| / | APRIN_exon_22 | Failed primer design | Failed primer design |
| 1015423 | APRIN_exon_23 | TGTAAAACGACGGCCAGTATTCTTTTGCCATTTTCTTGGA | CAGGAAACAGCTATGACCGAAACAGCACCATTGAACAG |
| 1011069 | APRIN_exon_24 | TGTAAAACGACGGCCAGTCTCCTAGGTACTTGCATTTCAACA | CAGGAAACAGCTATGACCCAGGGGCTTGAAGGAATCTAT |
| 1011070 | APRIN_exon_25 | TGTAAAACGACGGCCAGTCTTATTAGGGCCCAGATGAATA | CAGGAAACAGCTATGACCTTCTGTTAAAAGTACCTCCAATGTC |
| 1015424 | APRIN_exon_26 | TGTAAAACGACGGCCAGTTCATGTACCTCAACACAGGGTAT | CAGGAAACAGCTATGACCGAGAGCAAATAAAGAACCTTAAACAC |
| 1015425 | APRIN_exon_27 | TGTAAAACGACGGCCAGTGGAAATATTAATGGCTAAAAATGAA | CAGGAAACAGCTATGACCACAGATACTTAAGCTGGCAATCTA |
| 1011073 | APRIN_exon_28 | TGTAAAACGACGGCCAGTTGAGCAAAACCAGTAGTGTAAGC | CAGGAAACAGCTATGACCCCAGGGTCTTAGGCATGTTCT |
| 1011074 | APRIN_exon_29 | TGTAAAACGACGGCCAGTATATTAGAGTTGGCATGAGGTTG | CAGGAAACAGCTATGACCTATTAACCTAAGAAGTCTAAGAATCAGG |
| 1011075 | APRIN_exon_30 | TGTAAAACGACGGCCAGTTGCTTAAGGATAGACACATTTTTCA | CAGGAAACAGCTATGACCAACTTAAAGATTCCATAATGCTGCT |
| 1011076 | APRIN_exon_31 | TGTAAAACGACGGCCAGTCTTCATTGCTTGTTGAGTTGGTA | CAGGAAACAGCTATGACCTTTAAGGGTCCTCATTCCATTC |
| 1015426 | APRIN_exon_32 | TGTAAAACGACGGCCAGTTTTTAGGTACATATCTAGGACAACATT | CAGGAAACAGCTATGACCGTCGGCCTCTTTTACCCTTTT |
| 1015427 | APRIN_exon_32;APRIN_exon_33 | TGTAAAACGACGGCCAGTAGTCAGCGAAGTCGGAAAAGAG | CAGGAAACAGCTATGACCTTTTGATGGTCTTCCTCGTCCT |
| 1015428 | APRIN_exon_33 | TGTAAAACGACGGCCAGTCATGTGTAACTCTAAACTGCATCTG | CAGGAAACAGCTATGACCTGGTCAATGTGTGGTGATTATTT |
| 1011079 | APRIN_exon_34 | TGTAAAACGACGGCCAGTCTGAGGGTAAAGATGATATGACGAT | CAGGAAACAGCTATGACCTGAAACCCAAATAGAAATACCAAAC |
| 1015430 | APRIN_exon_35 | TGTAAAACGACGGCCAGTTTTATCTGCGGCTTTAAACTGAC | CAGGAAACAGCTATGACCGATGATAAAATGGGATAAAGAATTCAG |
| 1015431 | APRIN_exon_35 | TGTAAAACGACGGCCAGTCAAAGTTTTGATGTCTTGAGTCTCC | CAGGAAACAGCTATGACCAAGAAGATATAGAAAGCAATTTAACCT |
| / | APRIN_exon_35 | Not targeted | Not targeted |
| 1011082 | APRIN_exon_35 | TGTAAAACGACGGCCAGTCCAGTTCTCCGTAACAGTGTAAA | CAGGAAACAGCTATGACCTGCCCCATCTATTATAACCAATC |
| 1011083 | APRIN_exon_35 | TGTAAAACGACGGCCAGTTTTCTTCATTGTAATCTGCAAAATG | CAGGAAACAGCTATGACCAACATGCCTTTGTACTGCTGAA |
| / | APRIN_exon_35 | Not targeted | Not targeted |
| 1011086 | APRIN_exon_35 | TGTAAAACGACGGCCAGTAAAGGTTAAATTGCTTTCTATATCTTC | CAGGAAACAGCTATGACCAGCAGGGACTTACACTATCTTGA |
| 1015429 | APRIN_exon_35 | TGTAAAACGACGGCCAGTCTGAAGAGTGGACAGTTGGA | CAGGAAACAGCTATGACCAAGGGTGCCCAGGAGTAAAC |
| 1015432 | APRIN_exon_35 | TGTAAAACGACGGCCAGTTGAGGTTATCTTGCTGCACTCT | CAGGAAACAGCTATGACCACTGAGACGTGTTTCCACTTCA |
| / | CHTF18_exon_1 | Not targeted | Not targeted |
| 1011187 | CHTF18_exon_03;CHTF18_exon_2 | TGTAAAACGACGGCCAGTGCCGCTGACAATCTCCTCTC | CAGGAAACAGCTATGACCACCCTTGGATATGGGTGTCC |
| 1011188 | CHTF18_exon_04;CHTF18_exon_3 | TGTAAAACGACGGCCAGTAGGTGGTCAAGAGGCTGAACT | CAGGAAACAGCTATGACCACCCGAGGCCATACAAACAG |
| 1011189 | CHTF18_exon_06;CHTF18_exon_5;  CHTF18_exon_7 | TGTAAAACGACGGCCAGTCAGTCTCCCACCCCTCACT | CAGGAAACAGCTATGACCGTTCTTCCATCTTGGGAACAGA |
| 1015364 | CHTF18_exon_08 | TGTAAAACGACGGCCAGTCATGGTGGCAGGTGGACT | CAGGAAACAGCTATGACCCCTTCAGGGACAACGACTTC |
| 1011190 | CHTF18_exon_08;CHTF18_exon_7 | TGTAAAACGACGGCCAGTGAGTTGTTTGTGGCTCAGGAC | CAGGAAACAGCTATGACCGTTGGTGAACTGCGGGAAG |
| 1015365 | CHTF18_exon_09 | TGTAAAACGACGGCCAGTGCACGAACTTTGCTTTGTG | CAGGAAACAGCTATGACCATTTGCGTGGAGACCGTCA |
| 1015366 | CHTF18_exon_10 | TGTAAAACGACGGCCAGTACGGTCTCCACGCAAATG | CAGGAAACAGCTATGACCAGCCTCTCCTGACTGTGG |
| 1015367 | CHTF18_exon_12;  CHTF18_exon_11 | TGTAAAACGACGGCCAGTCCTTGATGCCTGGGTAGGT | CAGGAAACAGCTATGACCATCAGCCACACGGCTCCT |
| 1011194 | CHTF18_exon_13;CHTF18_exon_12;CHTF18_exon_14;CHTF18_exon_11 | TGTAAAACGACGGCCAGTTCATGAGGCCCATTATCTG | CAGGAAACAGCTATGACCGGAAGACCTCCTGCCACAC |
| 1011195 | CHTF18_exon_13;CHTF18_exon_14 | TGTAAAACGACGGCCAGTCCTCTGTGAGAAAACTGACAATGA | CAGGAAACAGCTATGACCGCAAAAATGAGGGTGCAACT |
| 1011196 | CHTF18_exon_15 | TGTAAAACGACGGCCAGTGGCTTAAGGGAATGTTTCAGG | CAGGAAACAGCTATGACCTGCATCTTAAGTCCAGAATCCA |
| 1015368 | CHTF18_exon_16 | TGTAAAACGACGGCCAGTAGTGACCCCTTGCTGGTGT | CAGGAAACAGCTATGACCGGAAGGTGATCCTGGGTGT |
| 1011198 | CHTF18_exon_18;CHTF18_exon_17 | TGTAAAACGACGGCCAGTGACCAGGCCTTGGCTCAC | CAGGAAACAGCTATGACCGCTCTGCAGCAAAACTCAGGT |
| 1011200 | CHTF18_exon_20 | TGTAAAACGACGGCCAGTACAATGCCTGCTCCCTACAG | CAGGAAACAGCTATGACCCAAGACAGGAGGACCGAGAG |
| 1011199 | CHTF18_exon_20;CHTF18_exon_19 | TGTAAAACGACGGCCAGTTCATCATCATCCCACGTACACT | CAGGAAACAGCTATGACCCATGACTGCCCTAGCCACAC |
| 1011201 | CHTF18_exon_22;CHTF18_exon_21 | TGTAAAACGACGGCCAGTGGCTGTATTGGCTCTTCTTGAT | CAGGAAACAGCTATGACCCCCCACTCACACCATCCTAT |
| 1011181 | CHTF8_exon_04 | TGTAAAACGACGGCCAGTGGGGCTTAACTTTTAGTGTAGAGG | CAGGAAACAGCTATGACCATGTAGTCCCCTGCTCACCATT |
| 1011182 | CHTF8_exon_04 | TGTAAAACGACGGCCAGTAGTGAAGAGGGAGTTGGATGAG | CAGGAAACAGCTATGACCGCAATGTACCCAAATGGAATGT |
| 1011183 | CHTF8_exon_04 | TGTAAAACGACGGCCAGTTCACATGCCACAAATAACATCTC | CAGGAAACAGCTATGACCGGGTAAATGCCAATCCCTTTC |
| 1011184 | CHTF8_exon_04 | TGTAAAACGACGGCCAGTAGTGGCCCTTGGGAAGTTAG | CAGGAAACAGCTATGACCCTGAGAGCCGGTGTTCTGTTA |
| 1011185 | CHTF8_exon_04 | TGTAAAACGACGGCCAGTAGACCTGCTGCTCTTAGAATGG | CAGGAAACAGCTATGACCGGTTCTCTTGGTGGAAATCTGA |
| 1011126 | CSPG6_exon_01 | TGTAAAACGACGGCCAGTCCTGAACTTGACTCCTCCTACG | CAGGAAACAGCTATGACCGAAGCCACCTTTCCACACAT |
| 1015439 | CSPG6_exon_02 | TGTAAAACGACGGCCAGTGAAAAACAAGAAAGAAATGCAAGA | CAGGAAACAGCTATGACCACAAGAACAGAAGAGCGGAAAT |
| 1011127 | CSPG6_exon_02 | TGTAAAACGACGGCCAGTGCAGATTTTTAATCACCACTTTCC | CAGGAAACAGCTATGACCTGTCTCTGAAATAAGGCAACAGTC |
| 1011128 | CSPG6_exon_03 | TGTAAAACGACGGCCAGTTTGTTTTATTGGTTTCTTTTGTTCAC | CAGGAAACAGCTATGACCTTTTTATACCTCTTCATCCTTTACAA |
| 1011129 | CSPG6_exon_04 | TGTAAAACGACGGCCAGTTCCTACCTGATTAGTCTGATGTGC | CAGGAAACAGCTATGACCTACATGGAGGACATACCCACCT |
| 1015440 | CSPG6_exon_05 | TGTAAAACGACGGCCAGTGCCAAAAGAGCAGAGACGTTAG | CAGGAAACAGCTATGACCCAACAGTTTGTACATTCCCATTTG |
| 1011130 | CSPG6_exon_06 | TGTAAAACGACGGCCAGTTCCCCAAAGTATGTATCCCTCT | CAGGAAACAGCTATGACCTTACGTGACCATCTTCTTGTCTAA |
| 1011131 | CSPG6_exon_06 | TGTAAAACGACGGCCAGTGGGCAAGGACTTTAAAATGAC | CAGGAAACAGCTATGACCAAGAATACTTTTTACTCTTTTGTAAGGA |
| 1011132 | CSPG6_exon_07 | TGTAAAACGACGGCCAGTAAAGTTCTGAGGATGATACAGATGG | CAGGAAACAGCTATGACCAAGTTTGTAGTTTGGCTGCTG |
| 1015442 | CSPG6_exon_08 | TGTAAAACGACGGCCAGTGATTTGGGTTGCTCATCTGG | CAGGAAACAGCTATGACCCATCATTGGTGTTATGGATTCT |
| 1011134 | CSPG6_exon_09 | TGTAAAACGACGGCCAGTGTTACCACATCACATCAAAGAAATAA | CAGGAAACAGCTATGACCTTCAAAATGGCTTTCCTCTCAT |
| 1011135 | CSPG6_exon_10 | TGTAAAACGACGGCCAGTAGTTCAAGGAAATTAGCCATGTG | CAGGAAACAGCTATGACCTATATTTAAAAACTAGACAGGTGTGGTG |
| 1011136 | CSPG6_exon_11;CSPG6_exon_12 | TGTAAAACGACGGCCAGTCCATAGTAAACCCTTTTGTACCAG | CAGGAAACAGCTATGACCTGCCAGTTCTTTCTGCTTTTCT |
| 1015443 | CSPG6_exon_12&13 | TGTAAAACGACGGCCAGTCTTGGTACTGTCCCTTTGTTTC | CAGGAAACAGCTATGACCAGCCAATCTACACAAAATAGAAGC |
| 1011138 | CSPG6_exon_13;CSPG6_exon_12 | TGTAAAACGACGGCCAGTCGAGGAATTGCTAGGTCTGAG | CAGGAAACAGCTATGACCAATCTACATAAAGGCTCAATGACA |
| 1011139 | CSPG6_exon_15;CSPG6_exon_14 | TGTAAAACGACGGCCAGTCCACACAGTTAAGACACCACACA | CAGGAAACAGCTATGACCTATCACTTGGAACCCATGACCT |
| 1011140 | CSPG6_exon_16 | TGTAAAACGACGGCCAGTTTGTTGGAGAGGATGTTTAGTTT | CAGGAAACAGCTATGACCAACAGAGTAAGACAAATCTTTATTGG |
| 1011141 | CSPG6_exon_17 | TGTAAAACGACGGCCAGTCGCCATTGGAATTTTTAAAGTA | CAGGAAACAGCTATGACCTGCTCTCAAAGTATCCTGTACTTACG |
| 1011142 | CSPG6_exon_18 | TGTAAAACGACGGCCAGTCTACTTCAGGGAAAACGCCAAT | CAGGAAACAGCTATGACCTCAGACATGCATACAGCTCAACT |
| 1011143 | CSPG6_exon_19 | TGTAAAACGACGGCCAGTCTTTCAATTCTCATTATCTACTTAAAGC | CAGGAAACAGCTATGACCTGTACAATTCACAAACTGATCCT |
| 1011144 | CSPG6_exon_20 | TGTAAAACGACGGCCAGTAATTATTCCACACATGGCAAGA | CAGGAAACAGCTATGACCAAAAAGTATGGGTCCAAACAA |
| 1011145 | CSPG6_exon_21 | TGTAAAACGACGGCCAGTAGTTTAATCATGGAATATGATCTGC | CAGGAAACAGCTATGACCTGGTAAGGCTAACAAGCTCAAA |
| 1011146 | CSPG6_exon_22 | TGTAAAACGACGGCCAGTGGGCCTTTCTTGCTAACTAGG | CAGGAAACAGCTATGACCAAACACATACACACCTGTTCTACTT |
| 1015444 | CSPG6_exon_23 | TGTAAAACGACGGCCAGTTTTATTCAGACAATAGCCATAGAAAA | CAGGAAACAGCTATGACCTGGTCTCAAAATTGTCATAAACA |
| 1011148 | CSPG6_exon_25;CSPG6_exon_24 | TGTAAAACGACGGCCAGTCTTCTAATTTTGTACTGACTTAACATGG | CAGGAAACAGCTATGACCAGCTTCATATTTCCGAAGTTCA |
| 1011149 | CSPG6_exon_26;CSPG6_exon_25 | TGTAAAACGACGGCCAGTCAGTGCAACACAGAATTAAAGAA | CAGGAAACAGCTATGACCCTTGAGACTGACTGCCCTCCA |
| 1011150 | CSPG6_exon_26;CSPG6_exon_27 | TGTAAAACGACGGCCAGTGGATCTAGTCTGTTATCCTTGTTCTT | CAGGAAACAGCTATGACCCAGAGCCTGGTCAATTTCAT |
| 1011151 | CSPG6_exon_27;CSPG6_exon_28 | TGTAAAACGACGGCCAGTTCTGCCCTTTAGGATATTAACTCA | CAGGAAACAGCTATGACCGCAACGCCATAAAATACTGCTA |
| 1011152 | CSPG6_exon_29 | TGTAAAACGACGGCCAGTTGTAGTTAAATGTAGTTGATAGGCTGT | CAGGAAACAGCTATGACCATGGTAGGGGCTGATTTTGT |
| 1011047 | DCC1_exon_01 | TGTAAAACGACGGCCAGTGAACAGGCAGCTTGCTATGAG | CAGGAAACAGCTATGACCGGAAACCAGGGACGGAGAT |
| 1011046 | DCC1_exon_02 | TGTAAAACGACGGCCAGTTTCTCCCTGATTATAGATTTTACACG | CAGGAAACAGCTATGACCCAGGATGCTTTGTCTTGTGTTC |
| 1011045 | DCC1_exon_03 | TGTAAAACGACGGCCAGTCTATGTGTGCTTTGGGGTGTG | CAGGAAACAGCTATGACCTTTCTGGGCATACTCTAGTTTTCA |
| 1011044 | DCC1_exon_04 | TGTAAAACGACGGCCAGTGACACCATCCTTAAACCCAAAA | CAGGAAACAGCTATGACCCTTCTCCATCTTCTCAAACTGAAAC |
| 1011040 | DCC1_exon_05 | TGTAAAACGACGGCCAGTGCGCCCAGCCTATTTCTTTA | CAGGAAACAGCTATGACCGTTCTTTGGTAAATATAACTTTTGAAAT |
| 1015349 | DCC1_exon_06 | TGTAAAACGACGGCCAGTACTGCACCCAGCCAGAGTT | CAGGAAACAGCTATGACCGGGCCTGGCACATAGTAGCTT |
| 1015348 | DCC1_exon_07 | TGTAAAACGACGGCCAGTGTTCCAAATTCAGCTCCAACTC | CAGGAAACAGCTATGACCGAACTATGGGGTTGTCGGAAGT |
| 1011042 | DCC1_exon_08 | TGTAAAACGACGGCCAGTTTTTAGAAGCAATAGTGAGGAACAGA | CAGGAAACAGCTATGACCGCTAACACGATGAATCCAAGAA |
| 1011041 | DCC1_exon_09 | TGTAAAACGACGGCCAGTAGACTCCCTCTCAAAATAAATAAATAG | CAGGAAACAGCTATGACCTCTCAAGTGAGTGAGTTCCCCTCT |
| 1015375 | DDX11_exon_02 | TGTAAAACGACGGCCAGTGCTAGATGGAGTGCGTGATTC | CAGGAAACAGCTATGACCAGGAAAAGCTAGTACGGCTGCT |
| 1010839 | DDX11_exon_03 | TGTAAAACGACGGCCAGTGCCCAGTTTCCATTTCTTTCT | CAGGAAACAGCTATGACCGGTCCTTTCCCAGGTGAGTAAT |
| 1015376 | DDX11_exon_04 | TGTAAAACGACGGCCAGTGCAAATGGATCTGATGGAGAG | CAGGAAACAGCTATGACCCTGGCTGGAGAGAGGTCGA |
| 1010841 | DDX11_exon_05 | TGTAAAACGACGGCCAGTTATGTGAATGGGGCGTGGT | CAGGAAACAGCTATGACCCCTTGATGGTTGGCACCT |
| 1015377 | DDX11_exon_05 | TGTAAAACGACGGCCAGTGCTTTGTCTCTGGCATGTGG | CAGGAAACAGCTATGACCGTGTATAAGGCGCAGACACA |
| 1010842 | DDX11_exon_06 | TGTAAAACGACGGCCAGTCACCTGCTACCTTGGTGGAAC | CAGGAAACAGCTATGACCCATAGCCCTCCATGCAAATG |
| 1010843 | DDX11_exon_07;DDX11_exon_8 | TGTAAAACGACGGCCAGTTGTTTTGGTTCTCTCTTTGAAGC | CAGGAAACAGCTATGACCTAAAATCATCTCCAAGCCAAGG |
| 1010844 | DDX11_exon_09 | TGTAAAACGACGGCCAGTGTGTTCCGATGAGACCACAGTA | CAGGAAACAGCTATGACCTCACTTGGAAAGCCATTTGTAA |
| 1010845 | DDX11_exon_10 | TGTAAAACGACGGCCAGTTTGCCACAAGCTGTTTTTCG | CAGGAAACAGCTATGACCATGATAGGGCAGCAGGAGGA |
| 1010846 | DDX11_exon_11;DDX11_exon_12 | TGTAAAACGACGGCCAGTCCTAAGGGCTGTGGAAACC | CAGGAAACAGCTATGACCAAAGCAGCCCAGCGAGTT |
| 1010847 | DDX11_exon_14;DDX11_exon_13 | TGTAAAACGACGGCCAGTGCAAGCACGTGAGTCAGACAT | CAGGAAACAGCTATGACCGAGAGAGCAAGGACCTGGAGT |
| 1010848 | DDX11_exon_15 | TGTAAAACGACGGCCAGTTCCACCCTGAGGAGGACACT | CAGGAAACAGCTATGACCAGCAGTGGAGAGGAACGAGGT |
| 1015378 | DDX11_exon_16;DDX11_exon_17 | TGTAAAACGACGGCCAGTGTCCCCTTCGTCTCCACTCT | CAGGAAACAGCTATGACCGATGACCCTGCCGTCCTG |
| 1015379 | DDX11_exon_17 | TGTAAAACGACGGCCAGTTGTGTGGACCTGACCAGAGG | CAGGAAACAGCTATGACCAAGCCCACCTACCCCACAGT |
| 1010849 | DDX11_exon_18 | TGTAAAACGACGGCCAGTTGTCCTAGAGATTAAATGGTGTTT | CAGGAAACAGCTATGACCCACATGGAGGGAGCCGTACT |
| 1015380 | DDX11_exon_19 | TGTAAAACGACGGCCAGTTTGTGTGTACTTGCTGTCTCTTAG | CAGGAAACAGCTATGACCAGATGCAGGTGTGCTGCAAG |
| 1015381 | DDX11_exon_20 | TGTAAAACGACGGCCAGTCCCTCTCTGCAGTGTCTTGC | CAGGAAACAGCTATGACCGGCAGGGGCAGATATAACCA |
| 1015382 | DDX11_exon_21;DDX11_exon_22 | TGTAAAACGACGGCCAGTGAGGTGGGTCGCATTCTCT | CAGGAAACAGCTATGACCCTGGTGTGCGCTCTTAGGTT |
| 1015383 | DDX11_exon_22;DDX11_exon_23 | TGTAAAACGACGGCCAGTCTGGCTGTGAGGTTCTCCAGT | CAGGAAACAGCTATGACCTCTAATGGCAGCAGAGAGCA |
| 1015384 | DDX11_exon_23 | TGTAAAACGACGGCCAGTCTTGGGTCTGAGATCGTGTG | CAGGAAACAGCTATGACCTGTGATCAAAACTCAGCCTCTC |
| 1015385 | DDX11_exon_24 | TGTAAAACGACGGCCAGTGCTGAGGGAAGGGGTAGAAC | CAGGAAACAGCTATGACCCTGGGAGTGAAGGGGAAGAC |
| / | DDX11_exon_25 | Not targeted | Not targeted |
| / | DDX11_exon_26 | Failed primer design | Failed primer design |
| / | DDX11_exon_27 | Failed primer design | Failed primer design |
| 1015438 | DING_exon_02 | TGTAAAACGACGGCCAGTGCTACCAAAATTCCAGCTAACC | CAGGAAACAGCTATGACCTATAGTTTTATCTACTTGTAAGCTGTGG |
| 1015437 | DING_exon_03 | TGTAAAACGACGGCCAGTCCACCAATATAGAAAATCTGACCA | CAGGAAACAGCTATGACCAAAGAAGTGAAAAGGGTGATAAGG |
| 1011105 | DING_exon_04 | TGTAAAACGACGGCCAGTCTACCATAAAGAACTGAACCAATCA | CAGGAAACAGCTATGACCTTACTACTACATTTTGCTCCCACAT |
| 1011104 | DING_exon_05 | TGTAAAACGACGGCCAGTCTGAAACGAGAGGGATCACA | CAGGAAACAGCTATGACCTGGAAACTTACTGTGTAGCTGAATG |
| 1011103 | DING_exon_06 | TGTAAAACGACGGCCAGTGGAGTAGTACATATACAGTGGGTTGA | CAGGAAACAGCTATGACCCGTATCTTTAGGAAAATCAAGGAGA |
| 1011102 | DING_exon_07 | TGTAAAACGACGGCCAGTAGTTAAAGTATAAACACATGCACAAG | CAGGAAACAGCTATGACCTTTTTATGTTATCCAGGCTGATCT |
| 1015435 | DING_exon_08 | TGTAAAACGACGGCCAGTCTAATGCCCAACGGCTTCTG | CAGGAAACAGCTATGACCCCTCTAATGATAGCTTTTTGTTACCTT |
| 1015436 | DING_exon_08 | TGTAAAACGACGGCCAGTCGTTCAAAATCTCGTCCTCTGT | CAGGAAACAGCTATGACCAGTGTCTTCCTATTTCACCTCTATC |
| 1011101 | DING_exon_08 | TGTAAAACGACGGCCAGTGGCTTACTGAATTATCCATGACT | CAGGAAACAGCTATGACCCAGTCATTGTATTAACATCTTTTCTGA |
| 1011100 | DING_exon_09 | TGTAAAACGACGGCCAGTCATTCCCATGTTGTTAAGCATCT | CAGGAAACAGCTATGACCCACTGCATATCTCTTCTTGAACTG |
| / | DING_exon_10 | Not targeted | Not targeted |
| 1011098 | DING_exon_10 | TGTAAAACGACGGCCAGTTCCAATTAGGAATTTATAAGGAAGA | CAGGAAACAGCTATGACCGCATTACAGATACTAGGGGCAAA |
| 1011097 | DING_exon_11 | TGTAAAACGACGGCCAGTCCTCCTAAACCATGTGAACAAA | CAGGAAACAGCTATGACCAGGTAGGGAAAACTGGGACTTC |
| 1011095 | DING_exon_12 | TGTAAAACGACGGCCAGTTTGAAAAACAGGAAAATATACAGAGAAA | CAGGAAACAGCTATGACCGTAGATCAAAGACCAAGCTCACC |
| 1011096 | DING_exon_12 | TGTAAAACGACGGCCAGTGATCGTGGTCTCGGTCTCTATC | CAGGAAACAGCTATGACCCTATCCTTTGCCACCAGTGTTC |
| 1011094 | DING_exon_13 | TGTAAAACGACGGCCAGTATTTAGAGGCAGCAAAGCAAGA | CAGGAAACAGCTATGACCGCTGAATGTTTATATCAGAGGGTACA |
| 1011091 | DING_exon_14 | TGTAAAACGACGGCCAGTCAGCATGTAAAGTAACCAATTTAGC | CAGGAAACAGCTATGACCCTGATGAGGATGTCCCTGACC |
| 1011092 | DING_exon_14 | TGTAAAACGACGGCCAGTTGTTCTGTGGCATTTTCTGTGT | CAGGAAACAGCTATGACCAGGACATTATAGGACACCACCAGT |
| 1011093 | DING_exon_14 | TGTAAAACGACGGCCAGTTGAAAATACTTGACCATCTTCAG | CAGGAAACAGCTATGACCTCTTGCTTTGCTGCCTCTAAAT |
| 1015434 | DING_exon_15 | TGTAAAACGACGGCCAGTTGTCCCTTTCAACAGAATCAGA | CAGGAAACAGCTATGACCAGAGACAATATGGAACATAGAAAGTT |
| 1011089 | DING_exon_15 | TGTAAAACGACGGCCAGTGAACCAAATCTGCCTTAATGGT | CAGGAAACAGCTATGACCGATCGACAGGTCCATTCAAGAT |
| 1015433 | DING_exon_15 | TGTAAAACGACGGCCAGTAAGAAAATAGTTGTTTCCCACGA | CAGGAAACAGCTATGACCCAAGAACGAGATCGATATGAACA |
| 1011088 | DING_exon_16 | TGTAAAACGACGGCCAGTGGCCCTTTCTAATTCATAGACAAT | CAGGAAACAGCTATGACCAGCTGTTCTCATAATTGCTGTCC |
| 1011087 | DING_exon_17 | TGTAAAACGACGGCCAGTTGATTATGTTTTCTTTCATGTTGGTA | CAGGAAACAGCTATGACCAATTCATGCCCTTCCTTTACTC |
| / | DING_exon_18 | Not targeted | Not targeted |
| / | DING_exon_19 | Not targeted | Not targeted |
| 1015369 | ESCO1_exon_ 12 | TGTAAAACGACGGCCAGTGAGCACATACAGCAGTATCTAA | CAGGAAACAGCTATGACCCATTAGTTTTCAGTCCACTATG |
| 1010812 | ESCO1_exon_04 | TGTAAAACGACGGCCAGTCTCTCGCTAACAACTTAACAGG | CAGGAAACAGCTATGACCGAATCTTCACAAATGGAAAGTG |
| 1010813 | ESCO1_exon_04 | TGTAAAACGACGGCCAGTGTGAAAAACTGGTTCGTAATAAGC | CAGGAAACAGCTATGACCCGCAAGGTAGAACATCAGACAG |
| 1010814 | ESCO1_exon_04 | TGTAAAACGACGGCCAGTCAGCCAGAATTGGAAACACGC | CAGGAAACAGCTATGACCTTTCGGGACCACTGAAGTAGC |
| 1010815 | ESCO1_exon_04 | TGTAAAACGACGGCCAGTAACGACCTTCTTGAAACCTCTG | CAGGAAACAGCTATGACCCCTTGTCCAAAGTAAAGCAAGTG |
| 1010811 | ESCO1_exon_05 | TGTAAAACGACGGCCAGTTCTGAAAGCATGGGTGATAGTC | CAGGAAACAGCTATGACCAGACAGACGAAACTTGGAAAAA |
| 1010810 | ESCO1_exon_06 | TGTAAAACGACGGCCAGTTGTCCATAGTAAACAGTATGTATAGGC | CAGGAAACAGCTATGACCTGTTTTCATTTGTTTCTACTTAGAGATT |
| 1010805 | ESCO1_exon_07 | TGTAAAACGACGGCCAGTAGTCTGGCTTCCTTGGTTTC | CAGGAAACAGCTATGACCTTTTTCCCCTTCAAATGATGT |
| 1010809 | ESCO1_exon_08 | TGTAAAACGACGGCCAGTACATACTACACGTGCTTTGTACATATT | CAGGAAACAGCTATGACCGGGGCTTTAGGACTAAGTTTGTAA |
| 1010808 | ESCO1_exon_09 | TGTAAAACGACGGCCAGTAGTTTCACTGGAAATTTACCTTTT | CAGGAAACAGCTATGACCCTTCTTGTGGCTTTGGTAGTTG |
| 1010804 | ESCO1_exon_10 | TGTAAAACGACGGCCAGTTTCAGTAGAGAACAGGTTTTCACAAT | CAGGAAACAGCTATGACCAACTCTTTAGAGGATGCCCTGCT |
| 1010807 | ESCO1_exon_11 | TGTAAAACGACGGCCAGTACTTACAGATCCAATACACTGATAACC | CAGGAAACAGCTATGACCGTGCGGGAGGTAGAGAGAACTA |
| 1011004 | KIAA1212_exon_01 | TGTAAAACGACGGCCAGTCTCCAGAAGGGGAGTAAAAAT | CAGGAAACAGCTATGACCGGGTTAGGGACACACATTCA |
| 1011005 | KIAA1212_exon_01 | TGTAAAACGACGGCCAGTGTTCCGACCTCTACACGTTCC | CAGGAAACAGCTATGACCAGCAGACAGACCCCTTGCTCT |
| 1011003 | KIAA1212_exon_01;  KIAA1212_exon_2 | TGTAAAACGACGGCCAGTCTGAGCCCATAACATTAGCATTC | CAGGAAACAGCTATGACCCTCGCCCGGTACAGACATTT |
| 1011001 | KIAA1212_exon_04 | TGTAAAACGACGGCCAGTTGTCTGGTCTTACCTATAACTTACAAA | CAGGAAACAGCTATGACCGCACCTAAAACTTCAGATTTCCA |
| 1011000 | KIAA1212_exon_05 | TGTAAAACGACGGCCAGTCCTTAGGAGGTTATACAGTTATTGAAAA | CAGGAAACAGCTATGACCTGCACAGTGGAAAACCATTTA |
| 1010999 | KIAA1212_exon_06 | TGTAAAACGACGGCCAGTCAGTGTGCCAAGTGTAAGAAACTAT | CAGGAAACAGCTATGACCGAAAGGCTGTGTTGTTTGGTAA |
| 1010998 | KIAA1212_exon_07 | TGTAAAACGACGGCCAGTTGTTTACTAGCAATTTGGGGTTC | CAGGAAACAGCTATGACCGATTAAAAACCTTTTGCTCTTGAA |
| 1015414 | KIAA1212_exon_08 | TGTAAAACGACGGCCAGTCTTGTGAAAACACTGACACAAGC | CAGGAAACAGCTATGACCATTTGCTAAGTAGCCCATGC |
| 1010996 | KIAA1212_exon_09 | TGTAAAACGACGGCCAGTAAAAAGAAGAAGCTGCTGTAAAA | CAGGAAACAGCTATGACCAGATTAGGGTTATGGCTAATACTATGT |
| 1015413 | KIAA1212_exon_10 | TGTAAAACGACGGCCAGTACTGTTTGTTCACCATTTCAAGA | CAGGAAACAGCTATGACCAAGCTGAGGCAGGAGAAGTG |
| 1010994 | KIAA1212_exon_11 | TGTAAAACGACGGCCAGTCCTCAAGGACACTAGCCATCC | CAGGAAACAGCTATGACCGATTTATGTGAGTTCTGGTTTAAGTC |
| 1010993 | KIAA1212_exon_12 | TGTAAAACGACGGCCAGTATGTAAAGCTAGGGACCAGACC | CAGGAAACAGCTATGACCTCTTCAACCTTTTAATACCACAATAAAT |
| / | KIAA1212_exon_13 | Not targeted | Not targeted |
| 1010991 | KIAA1212_exon_14 | TGTAAAACGACGGCCAGTAGCTTTCTCTTCAACTTTCCTAAGAC | CAGGAAACAGCTATGACCAAATCCACTCAAGCCAAGTGA |
| 1010988 | KIAA1212_exon_15 | TGTAAAACGACGGCCAGTAACAAAGTAGGAAACTTGGAAGC | CAGGAAACAGCTATGACCTAGAAAATCAAAGACTGCAAAAA |
| 1010989 | KIAA1212_exon_15 | TGTAAAACGACGGCCAGTTTTCCAGTTGAGAAGTCTCTTGC | CAGGAAACAGCTATGACCAAAGAAACAAGTAGCAAGCTAAGCA |
| 1010990 | KIAA1212_exon_15 | TGTAAAACGACGGCCAGTAGCTCTGAATTTTCTTGTTCTAAGG | CAGGAAACAGCTATGACCTGTGTGTGTGTTACCTGTATTCCTA |
| 1010987 | KIAA1212_exon_16 | TGTAAAACGACGGCCAGTAAATTTTCAACTGTTTGCCAAG | CAGGAAACAGCTATGACCCATTGAGAGGGACCTTTTATTTTT |
| 1010986 | KIAA1212_exon_17 | TGTAAAACGACGGCCAGTACACATGAAATAGCACTGAATAAGC | CAGGAAACAGCTATGACCGACTTTGGTCTTCCGGGTTC |
| 1010985 | KIAA1212_exon_18 | TGTAAAACGACGGCCAGTGCTGAGGGAAGCCAGTCATA | CAGGAAACAGCTATGACCACTGTTAATCCAGGGGCATTT |
| 1010984 | KIAA1212_exon_19 | TGTAAAACGACGGCCAGTATGTGTGAAAATTTGGCATGG | CAGGAAACAGCTATGACCACACAGCCCCATTTATTCTGG |
| 1015411 | KIAA1212_exon_20 | TGTAAAACGACGGCCAGTTTTTAGAAATGAAGGAAATGAGTAATGA | CAGGAAACAGCTATGACCACCAGAATGCCCAACTCCTAAT |
| 1010983 | KIAA1212_exon_20 | TGTAAAACGACGGCCAGTTCAAGGTCTCTATGTTCCACCTC | CAGGAAACAGCTATGACCTTTTTCACTTCTATCAACTTCTATCACC |
| 1015412 | KIAA1212_exon_20 | TGTAAAACGACGGCCAGTTCAAGGTCTCTATGTTCCACCTC | CAGGAAACAGCTATGACCAAAATCCACGTCTTCCCAGTAT |
| 1015410 | KIAA1212_exon_21 | TGTAAAACGACGGCCAGTAGGGGCAGTATATAGTTTGGATG | CAGGAAACAGCTATGACCGAGACCTTGAAGACCGGTAATTT |
| 1010981 | KIAA1212_exon_22 | TGTAAAACGACGGCCAGTCAGTGATCCTAGACCAAATTCTTTT | CAGGAAACAGCTATGACCAGTGTGGAGATGGGTGGATGA |
| 1010980 | KIAA1212_exon_23 | TGTAAAACGACGGCCAGTGAATAGTGCCTGGAATATAAGCA | CAGGAAACAGCTATGACCATCCTTGCAACAGCCTTGT |
| 1010978 | KIAA1212_exon_25;  KIAA1212_exon_24 | TGTAAAACGACGGCCAGTCCTCAGAAAATTGTGTGGCTGT | CAGGAAACAGCTATGACCGCTGTACAAAATATGATGCCTGTC |
| 1010979 | KIAA1212_exon_25;  KIAA1212_exon_24 | TGTAAAACGACGGCCAGTGAGTAATCCAGTTGCCTCTCCT | CAGGAAACAGCTATGACCTGGTTCTCTACAAGTAAATCAAACCT |
| / | KIAA1212_exon_26 | Not targeted | Not targeted |
| 1010977 | KIAA1212_exon_27 | TGTAAAACGACGGCCAGTTGAAAGTTCCTCCAAATCCAATA | CAGGAAACAGCTATGACCTAGCACATCGCTGCACAATC |
| 1010976 | KIAA1212_exon_28 | TGTAAAACGACGGCCAGTTTAGGTTTGAAGAAATGGTAGAGGT | CAGGAAACAGCTATGACCATTGGATTTGGAGGAACTTTCA |
| 1010975 | KIAA1212_exon_29 | TGTAAAACGACGGCCAGTTGATTTGGATTTCATGCTTTACC | CAGGAAACAGCTATGACCCAATGTGCAGAGTTCCTTGTCT |
| 1010974 | KIAA1212_exon_30 | TGTAAAACGACGGCCAGTAGAAATTTAACTTGTGGTGTTCAT | CAGGAAACAGCTATGACCTTCACTTATTTATTCATTCATTTACTCA |
| 1011002 | KIAA1212_exon_3 | TGTAAAACGACGGCCAGTTGTAAGTAGTTATTAAAACCCCAGATTT | CAGGAAACAGCTATGACCTTTTTCTTCTGCAGTTTCTGCT |
| 1010973 | KIAA1212_exon_31 | TGTAAAACGACGGCCAGTTTCTAGCCTGGGCATATAGAGG | CAGGAAACAGCTATGACCAGTTAAGGAGTATTAAGAGGTACCACAA |
| 1010972 | KIAA1212_exon_32 | TGTAAAACGACGGCCAGTCAGCCAACTAATCTTGCTTCAC | CAGGAAACAGCTATGACCGCTTTAACCAATGTAAGGAGGAAC |
| 1010829 | LEO1_exon_01 | TGTAAAACGACGGCCAGTGTCTGCTCCACCGACACC | CAGGAAACAGCTATGACCTCAGTAAGAAACAAAGGGCTTCA |
| 1010826 | LEO1_exon_02 | TGTAAAACGACGGCCAGTTCCCCCAGACCTATCCATT | CAGGAAACAGCTATGACCGGAGGGAGCCATCATTCAG |
| 1010827 | LEO1_exon_02 | TGTAAAACGACGGCCAGTAAGCTACCGGCCGTTCAT | CAGGAAACAGCTATGACCTGTTTATATGACCCCATAGATTCTGA |
| 1010828 | LEO1_exon_02 | TGTAAAACGACGGCCAGTAGCTTCTGATCTATTGTCTGATCTTTC | CAGGAAACAGCTATGACCTCTTTGGTTGAGCCCCTCTAGT |
| 1010825 | LEO1_exon_03 | TGTAAAACGACGGCCAGTGAGGCAGGTTCTTCTTCAACTAA | CAGGAAACAGCTATGACCTCCCTTATGCTATCCCTTTTGA |
| 1010824 | LEO1_exon_04 | TGTAAAACGACGGCCAGTCCTCATGTTTGGCAGTTATTTC | CAGGAAACAGCTATGACCTTATGGAAAATGGGTGTCTGTG |
| 1010823 | LEO1_exon_05 | TGTAAAACGACGGCCAGTTAGCATTAAAAGCCTCCCTTAC | CAGGAAACAGCTATGACCCTTGTGATTTGAGAACCATTGA |
| 1010822 | LEO1_exon_06 | TGTAAAACGACGGCCAGTCATGGTGGCATGTTTTCTGT | CAGGAAACAGCTATGACCCTTCCTTGAAGTTAAGCATGAAGAT |
| 1010821 | LEO1_exon_07 | TGTAAAACGACGGCCAGTAATAATCCATGCAGGGTGGT | CAGGAAACAGCTATGACCTTCTGCCTCACATGGACTCAA |
| 1010820 | LEO1_exon_08 | TGTAAAACGACGGCCAGTACAGTCTCTTGTAAGTCTGGTTAGCA | CAGGAAACAGCTATGACCTTCCCGGGTTCAAGCAGTTA |
| 1010819 | LEO1_exon_09 | TGTAAAACGACGGCCAGTGCCTGGTGAAGAGAGAACCTG | CAGGAAACAGCTATGACCTTAATGTTGCTTGAGCGTATCC |
| 1010818 | LEO1_exon_10 | TGTAAAACGACGGCCAGTTTGGCAGCCGGATTACATCT | CAGGAAACAGCTATGACCTATGGGAGTTTGGTAGAGCTG |
| 1010817 | LEO1_exon_11 | TGTAAAACGACGGCCAGTGCCTAGCCCTCCCAGAATAC | CAGGAAACAGCTATGACCTGACTCACAATCTGTACTTTTGCTTT |
| 1010816 | LEO1_exon_12 | TGTAAAACGACGGCCAGTGGAAAAGTAGATTTCTTAAACATAGAGA | CAGGAAACAGCTATGACCCCTGATCATGGTAAAAATATCCTG |
| 1011125 | MRE11A_exon_02 | TGTAAAACGACGGCCAGTTCAAATTACTGCAAGACTCCAATC | CAGGAAACAGCTATGACCGAACCAGAACCGTATGTGACC |
| 1011124 | MRE11A_exon_03 | TGTAAAACGACGGCCAGTTGAATTTAAGACACAAAGCATACAAA | CAGGAAACAGCTATGACCCCTGGAAGAGTACGAAGTCAGAT |
| 1011123 | MRE11A_exon_04 | TGTAAAACGACGGCCAGTGAAAATAGCTTATATGGAAGGCAAAA | CAGGAAACAGCTATGACCCGTTCTGTTAAAGTGCTAAGTTTATGT |
| / | MRE11A_exon_05 | Failed primer design | Failed primer design |
| 1011122 | MRE11A_exon_06 | TGTAAAACGACGGCCAGTTCAGGATTCTACACCTGAGTCTAAA | CAGGAAACAGCTATGACCCAGGCTTTAAGACGGTTGCTTA |
| 1015353 | MRE11A_exon_07 | TGTAAAACGACGGCCAGTAAATCTATGTTTTGTCTGATCTTGC | CAGGAAACAGCTATGACCAAGTGAAATACTTTGAGAAGGACA |
| 1011120 | MRE11A_exon_08 | TGTAAAACGACGGCCAGTTGAGCAGCAAAATAACACCAAT | CAGGAAACAGCTATGACCGGGGAAATCCTTCCTATGTAAAA |
| 1011119 | MRE11A_exon_09 | TGTAAAACGACGGCCAGTCAGTGTCCTTACAGGCTTCA | CAGGAAACAGCTATGACCGATCAGACAGGGATAATAATGGA |
| 1011118 | MRE11A_exon_10 | TGTAAAACGACGGCCAGTCCGATGGTGATTGCTCTTCTTA | CAGGAAACAGCTATGACCGATCCCTAAATCTTCTGGTGAG |
| 1011117 | MRE11A_exon_11 | TGTAAAACGACGGCCAGTACAATCATATTAAAACATCTTCCATT | CAGGAAACAGCTATGACCTGTTCCTAGCATTCATCTTTCTCTT |
| 1011116 | MRE11A_exon_12 | TGTAAAACGACGGCCAGTAAACACTAATTTTCCCTGCTGTG | CAGGAAACAGCTATGACCGCAGTACCCATGATTGCTTTT |
| 1011115 | MRE11A_exon_13 | TGTAAAACGACGGCCAGTCTATCCATGGGGAACAAAACAC | CAGGAAACAGCTATGACCTCATAGCTGCCATTTTTGGACT |
| 1015352 | MRE11A_exon_14 | TGTAAAACGACGGCCAGTCAGGTTTTAGACAAGAACAAAATGG | CAGGAAACAGCTATGACCAATTGGAAAAATATGATTTACTTTTGTG |
| 1011113 | MRE11A_exon_15 | TGTAAAACGACGGCCAGTAGTGAAGCTTTTATAAGGTATGTGC | CAGGAAACAGCTATGACCAGCCACTGTGTCAGCCTCCT |
| 1011112 | MRE11A_exon_16 | TGTAAAACGACGGCCAGTCCTGTGATCCTAATTGCCCTTAT | CAGGAAACAGCTATGACCTCATGCCAGTTAATTTTTGTATTTTT |
| 1015351 | MRE11A_exon_17 | TGTAAAACGACGGCCAGTAAAATCAAATCCTAGAAGCCCTA | CAGGAAACAGCTATGACCACAGTATCTCAATAAGCTGGGAAA |
| 1011110 | MRE11A_exon_18 | TGTAAAACGACGGCCAGTACTGAAAATCCTTGTACTAATGCTG | CAGGAAACAGCTATGACCGTATTTCCCACATGGTCTGATA |
| 1015350 | MRE11A_exon_19 | TGTAAAACGACGGCCAGTAGCAACTAGCTGGCAGTCTC | CAGGAAACAGCTATGACCCAGTTAAGTTTTATTGCCGCTAA |
| 1011108 | MRE11A_exon_20 | TGTAAAACGACGGCCAGTACTTATGGAGTTATGCTCAGGAA | CAGGAAACAGCTATGACCGCTTGCTTTGCTAGGTTGTTTT |
| 1015445 | NIPBL_exon_02 | TGTAAAACGACGGCCAGTATAGGTTGAACAAACCAAAGCA | CAGGAAACAGCTATGACCAAATAGGCTGCTGATACCTCCA |
| 1011204 | NIPBL_exon_02 | TGTAAAACGACGGCCAGTTGATTAAGCATTTTCCTGATAGTAAT | CAGGAAACAGCTATGACCGCCATCCTCTCACTTTCTACCC |
| 1011205 | NIPBL_exon_03 | TGTAAAACGACGGCCAGTTTATGGTCCAAGTGATGTTTTG | CAGGAAACAGCTATGACCGCGCCCAGACAGGGATATAG |
| 1011206 | NIPBL_exon_04 | TGTAAAACGACGGCCAGTTATTGTTGGCCATACCAGTGT | CAGGAAACAGCTATGACCAACCAATTTTTCTGTTGCTAAGG |
| 1011207 | NIPBL_exon_05 | TGTAAAACGACGGCCAGTCAGTTTTACAGCGTCTATATTTTGC | CAGGAAACAGCTATGACCTTAAAGCCATGCTCCGTAA |
| 1011208 | NIPBL_exon_06 | TGTAAAACGACGGCCAGTGATTCTTCTGTTTGCATAGATTTTACA | CAGGAAACAGCTATGACCTAATTGTCCCTTGGCTTAGTGC |
| 1011209 | NIPBL_exon_07 | TGTAAAACGACGGCCAGTAACACATAGGCAGAATAAACTCAG | CAGGAAACAGCTATGACCTTATAAATGTAAAAACCATTCTCACTT |
| 1015446 | NIPBL_exon_08 | TGTAAAACGACGGCCAGTTTTATGTCTCTTATTGGTTCTCTTTT | CAGGAAACAGCTATGACCTTAAATCCCATGATCCAGACCT |
| 1011211 | NIPBL_exon_09 | TGTAAAACGACGGCCAGTGCTAAACAACTGTGTGGGACAA | CAGGAAACAGCTATGACCATTTGCTGGAAGGAATGCTGT |
| 1011212 | NIPBL_exon_09 | TGTAAAACGACGGCCAGTAATTAGTTCTCCATCCAAGGACTCT | CAGGAAACAGCTATGACCAATTACCAAATCTCATATAGTTGTTTCA |
| 1011213 | NIPBL_exon_10 | TGTAAAACGACGGCCAGTTCAAAGTATTGTGTCATTCATGCTTA | CAGGAAACAGCTATGACCTTAGGCTCAACTATGGTGCTCTC |
| 1011214 | NIPBL_exon_10 | TGTAAAACGACGGCCAGTTTCAGGAAGATATTGTTGGAAGTC | CAGGAAACAGCTATGACCGCCCATCAGGTCTCTGCTTTA |
| 1011215 | NIPBL_exon_10 | TGTAAAACGACGGCCAGTCAAAACAGAAGGGTGATGGAAG | CAGGAAACAGCTATGACCTCTGCCTTATTGTCAGGGTGT |
| 1011216 | NIPBL_exon_10 | TGTAAAACGACGGCCAGTCTTCTGGGGAACAAAAATCAAG | CAGGAAACAGCTATGACCTTGAGTAGTGGGTGGGAAGAAA |
| 1015447 | NIPBL_exon_11 | TGTAAAACGACGGCCAGTAAGAATTACCCCCTGAACTCCT | CAGGAAACAGCTATGACCGGTGCCAACTGATCTCATCTTT |
| 1011218 | NIPBL_exon_13;NIPBL_exon_12 | TGTAAAACGACGGCCAGTTGGGTAGATTGTGCTAAAATTAGTG | CAGGAAACAGCTATGACCGTTAGTTTTGGTTCATATGCTTTCC |
| 1011219 | NIPBL_exon_14;NIPBL_exon_13 | TGTAAAACGACGGCCAGTTTTCTATGTGCAGTGATTATCGTTT | CAGGAAACAGCTATGACCTTCTTGGTAACAGACTAGATTTGACTTT |
| 1015448 | NIPBL_exon_15 | TGTAAAACGACGGCCAGTAATCTGACTTCAATTTCTGTCCTTTT | CAGGAAACAGCTATGACCTCAATGCAGTAATATGATTCTCTTTA |
| 1011221 | NIPBL_exon_16 | TGTAAAACGACGGCCAGTTTTAAGAGGGTGACAATGCTATTT | CAGGAAACAGCTATGACCCATGCTCAAATTAACGAAATGT |
| 1011222 | NIPBL_exon_17 | TGTAAAACGACGGCCAGTACAAATCGTGCTCAAAGTAGTATCA | CAGGAAACAGCTATGACCTTGGGTAAAATTAGGGCGAAGT |
| 1011223 | NIPBL_exon_18 | TGTAAAACGACGGCCAGTAGGGATTTAATAGCTGCTGAAGA | CAGGAAACAGCTATGACCGCTATCAGTCCTCTAATCTCTACTTTTT |
| 1011224 | NIPBL_exon_19 | TGTAAAACGACGGCCAGTCCTGTTGTATATGCTAACGTGCTT | CAGGAAACAGCTATGACCTCTAAGGTAAGCTGCTCTATTTTCC |
| 1011225 | NIPBL_exon_20 | TGTAAAACGACGGCCAGTTCATTCTAAATGGCAGGTAATTTTT | CAGGAAACAGCTATGACCTCTTATAAAATCCTCTTTGAACTGAT |
| 1011226 | NIPBL_exon_21 | TGTAAAACGACGGCCAGTCACATAAGAACACAATAAGCACTAAGA | CAGGAAACAGCTATGACCTAAAGCCCAGCCATGAACCT |
| 1015450 | NIPBL_exon_22 | TGTAAAACGACGGCCAGTCATGACAATAGCAACAGGGCTA | CAGGAAACAGCTATGACCAATTCTCCAAAATTTACTGAAGACA |
| 1011228 | NIPBL_exon_23 | TGTAAAACGACGGCCAGTAAAGAATTTATGGTGATCTTTTAGG | CAGGAAACAGCTATGACCAAAAATTGTTAAAGCACTGTTATCTTA |
| 1015451 | NIPBL_exon_24 | TGTAAAACGACGGCCAGTGAAAATCAAAAGGCAAAAATGA | CAGGAAACAGCTATGACCAATTTCTCACTGGGGCTTTG |
| 1011229 | NIPBL_exon_25 | TGTAAAACGACGGCCAGTGTTACTGTGGTTGTATTTTCATTTTT | CAGGAAACAGCTATGACCCACTCGGCCCATACTCACTCT |
| 1011230 | NIPBL_exon_27;NIPBL_exon_26 | TGTAAAACGACGGCCAGTTTTTCTGGCTTTCTTAAAATCTGTT | CAGGAAACAGCTATGACCTATACTGCTCACAAGCATCCAG |
| 1011231 | NIPBL_exon_27;NIPBL_exon_26 | TGTAAAACGACGGCCAGTCACACCTTCTCAGTTTAGCACATT | CAGGAAACAGCTATGACCTGTAAAAGGGATGTAGAACAAGGAC |
| 1011232 | NIPBL_exon_29;NIPBL_exon_28 | TGTAAAACGACGGCCAGTTAAACGAAAGGCTCCAAAGTAT | CAGGAAACAGCTATGACCTTAGAAAGTGGCAATAGTTCATAAAT |
| 1011233 | NIPBL_exon_30 | TGTAAAACGACGGCCAGTGCAGAAAGCATGTAAAAAGCAA | CAGGAAACAGCTATGACCAGGCATGCTCTACTATGTTTGG |
| 1011234 | NIPBL_exon_31 | TGTAAAACGACGGCCAGTGGCAGTTTGTGTTTTGATTAGTT | CAGGAAACAGCTATGACCATTATTACCCTTGGGCGATTGT |
| / | NIPBL_exon_32 | Failed primer design | Failed primer design |
| 1015452 | NIPBL_exon_33 | TGTAAAACGACGGCCAGTTTTTGTTTTATTAATTTGCACTTTGAG | CAGGAAACAGCTATGACCAATCTAGGATGCAACATCAAACAG |
| 1011236 | NIPBL_exon_34 | TGTAAAACGACGGCCAGTTGAGGCCTATACTGGACCTATTT | CAGGAAACAGCTATGACCGACCGAGGTTGACTACTGTTCC |
| 1015453 | NIPBL_exon_34 | TGTAAAACGACGGCCAGTAAAATGAGATGGAGAAAGTCTAGTATCA | CAGGAAACAGCTATGACCTTACAGCAAAATGGAACCTGAA |
| 1015454 | NIPBL_exon_36;NIPBL_exon_35 | TGTAAAACGACGGCCAGTCTTTACGTGCAAAATGCCCTAT | CAGGAAACAGCTATGACCCAGCTCCAAGACAGCTCACAC |
| 1015456 | NIPBL_exon_37 | TGTAAAACGACGGCCAGTTTATTAGGTGCCATTTCAAAATTA | CAGGAAACAGCTATGACCAGAAGCTTGTTTCGGAATGTA |
| 1011238 | NIPBL_exon_38 | TGTAAAACGACGGCCAGTCAGACTGATACTTTGAATGCCACT | CAGGAAACAGCTATGACCTTTTAACCAGATGTGGCAGCTT |
| 1015457 | NIPBL_exon_39 | TGTAAAACGACGGCCAGTTAGGTAAGGCCACCAGCATATT | CAGGAAACAGCTATGACCGACCTCAGCATAAGGACTGCTA |
| 1011240 | NIPBL_exon_40 | TGTAAAACGACGGCCAGTGATTAAGAACCATTGAGCCAGAA | CAGGAAACAGCTATGACCGGACTAAGACTCCACCCTGTTG |
| / | NIPBL_exon_41 | Failed primer design | Failed primer design |
| 1011242 | NIPBL_exon_42 | TGTAAAACGACGGCCAGTTGTTCCTTCTAGCACTTCATTCTG | CAGGAAACAGCTATGACCCGGTTCCCAAAGCGATTAGT |
| 1011243 | NIPBL_exon_43 | TGTAAAACGACGGCCAGTAACATTAAGTGAGGTGAAAGTGC | CAGGAAACAGCTATGACCTCTATAACGGTGCTGTAGTTGAGG |
| 1011244 | NIPBL_exon_44 | TGTAAAACGACGGCCAGTTCCCCTAAGATTACATATCCAGTTG | CAGGAAACAGCTATGACCCTCCTGAGTAGCTGGGGTCAC |
| 1011245 | NIPBL_exon_45 | TGTAAAACGACGGCCAGTAGTAGAAAGAGGTAAACCAGTTAGGTG | CAGGAAACAGCTATGACCCAATTTCCCGCAGATACCAG |
| 1011246 | NIPBL_exon_46 | TGTAAAACGACGGCCAGTATGATGGCTAACGTCTGTTTCA | CAGGAAACAGCTATGACCAACTCTTAATTTGTACCCGTGTCTTT |
| 1015458 | NIPBL_exon_47 | TGTAAAACGACGGCCAGTGGAAGTGTGCCGGGAAAT | CAGGAAACAGCTATGACCGTACAAATTCATTAGCTGGAAGTC |
| 1015459 | NIPBL_exon_47 | TGTAAAACGACGGCCAGTTGGTGCCTTGGGTAGACACTAT | CAGGAAACAGCTATGACCAGCCATCCGCTGCTAGGTTA |
| 1011006 | REC8L1_exon_02;REC8L1_exon_3;  REC8L1_exon_4 | TGTAAAACGACGGCCAGTAATTGTGTATCTGCCCATTGTTC | CAGGAAACAGCTATGACCCTTTGAGGGTTCCCAGCCTTA |
| 1011007 | REC8L1_exon_04;REC8L1_exon_5;  REC8L1_exon_6 | TGTAAAACGACGGCCAGTGTAATCAGGGCGCATTGTTC | CAGGAAACAGCTATGACCCTGTGGGAGAGAGCTGTGGTA |
| 1011008 | REC8L1_exon_07 | TGTAAAACGACGGCCAGTGGAATTACAGGCACTGCTACC | CAGGAAACAGCTATGACCGGCTCTTAAGCTCAAAAATGGTCA |
| 1011010 | REC8L1_exon_09;REC8L1_exon_12;REC8L1_exon_11;REC8L1_exon_10 | TGTAAAACGACGGCCAGTCGAGAACTGGACCTGCTGAT | CAGGAAACAGCTATGACCGTCACCTCTGCAACAACACC |
| 1011009 | REC8L1_exon_09;REC8L1_exon_8;  REC8L1_exon_10 | TGTAAAACGACGGCCAGTAGGGTCAGGATGCCAGAGATTA | CAGGAAACAGCTATGACCGGGACTGTGGAGAGAGAATCAG |
| 1011011 | REC8L1_exon_12;REC8L1_exon_13;REC8L1_exon_11 | TGTAAAACGACGGCCAGTCTCAGGGTGGAAGGAATAGGAG | CAGGAAACAGCTATGACCCCTTGTCCCAGAACAGTAACC |
| 1011012 | REC8L1_exon_13 | TGTAAAACGACGGCCAGTCCCTTATGCAAGGTATGAGCTG | CAGGAAACAGCTATGACCGTAGTGGGGGAAGGTCAGAAG |
| 1015339 | REC8L1_exon_14;REC8L1_exon_15 | TGTAAAACGACGGCCAGTAGTCCTTCTTTTAGCAATGAGCA | CAGGAAACAGCTATGACCGCAGTAAACAGGTGGTGCAGT |
| 1015340 | REC8L1_exon_14;REC8L1_exon_16;REC8L1_exon_15 | TGTAAAACGACGGCCAGTCCCCAACTCTCTGTAAGAATGGT | CAGGAAACAGCTATGACCCACTGCGCCTCTTCTCTAGGT |
| 1011014 | REC8L1_exon_18;REC8L1_exon_16;REC8L1_exon_17 | TGTAAAACGACGGCCAGTTCCTGCTATGAGAGCCAACAG | CAGGAAACAGCTATGACCATCTCCATGGGCACCTCA |
| 1011015 | REC8L1_exon_19;REC8L1_exon_18;REC8L1_exon_17;REC8L1_exon_20 | TGTAAAACGACGGCCAGTCACCAGAAGAACGGTGGTAAG | CAGGAAACAGCTATGACCTTTACTAGAAGTGGCCGGTTTC |
| 1010971 | SCC112_exon_02 | TGTAAAACGACGGCCAGTCTTTCAGAGGGGGTAAGAGAT | CAGGAAACAGCTATGACCCAAACTTCGGACCCTGGTG |
| 1010970 | SCC112_exon_03 | TGTAAAACGACGGCCAGTAAAGGCAGAACAACCAAGATG | CAGGAAACAGCTATGACCTTTTTAAATCACATCCTCCACTG |
| / | SCC112_exon_04 | Failed primer design | Failed primer design |
| 1015402 | SCC112_exon_05 | TGTAAAACGACGGCCAGTTTTTTACTTTCCCCTTTATGTTAAGTTT | CAGGAAACAGCTATGACCTCTTTTTCAAGGTCAGAATATGTGAT |
| 1015401 | SCC112_exon_06 | TGTAAAACGACGGCCAGTAAGAAATCCAAACTCCTATGTTTCA | CAGGAAACAGCTATGACCGGTACAAATGCACATGCTAGA |
| 1010967 | SCC112_exon_07 | TGTAAAACGACGGCCAGTAGTTGCAGACGACTTTTCTTATAG | CAGGAAACAGCTATGACCGAGCATGTTGTGTTAGATCAGG |
| 1015400 | SCC112_exon_08 | TGTAAAACGACGGCCAGTATTCAAGCTGTGGCATGACG | CAGGAAACAGCTATGACCTTCCTAGAAATGTCCTTATTTGAA |
| 1010965 | SCC112_exon_09 | TGTAAAACGACGGCCAGTCAATTTATTCCATAAGAAGGATGGA | CAGGAAACAGCTATGACCATATCAAAACTTTAATTGATGTGTGTTT |
| 1010964 | SCC112_exon_10 | TGTAAAACGACGGCCAGTGAGACCATACCAGAGTTAAAACC | CAGGAAACAGCTATGACCTCTCCTATAAACTTATTCTTCAAACTGA |
| 1010963 | SCC112_exon_11 | TGTAAAACGACGGCCAGTTGCAGGAAACATACAAATTAGGAG | CAGGAAACAGCTATGACCGGGCCACTACACTTCTGGCTA |
| 1010962 | SCC112_exon_12 | TGTAAAACGACGGCCAGTCCAGTTTATAAAGGCACACATAAGA | CAGGAAACAGCTATGACCGCTACCTTATAATCGTGGACAGTCT |
| 1010961 | SCC112_exon_13 | TGTAAAACGACGGCCAGTAAAAATCTATTAAAGTGAAATGACCACA | CAGGAAACAGCTATGACCTTGCTTCTTAAAAATGGAATAATTG |
| 1015399 | SCC112_exon_14 | TGTAAAACGACGGCCAGTTGAGATACTTTTTCCCTGCTTCA | CAGGAAACAGCTATGACCTTTAGTACATCTTTCAATCCCTGT |
| 1010959 | SCC112_exon_15 | TGTAAAACGACGGCCAGTAAAACTATTCTACTAAGCTAAAAACAGG | CAGGAAACAGCTATGACCTTTAAGTAACGGGCCACAAA |
| 1015398 | SCC112_exon_16 | TGTAAAACGACGGCCAGTGCACATGTACACAAGCATTCAG | CAGGAAACAGCTATGACCTTTTTCCTTCTCTAATATTGATTCTCT |
| 1010958 | SCC112_exon_16 | TGTAAAACGACGGCCAGTAGCTGAATAAATGAATGCATAAGT | CAGGAAACAGCTATGACCATTTTATAGAGAATTTGCCTGACC |
| 1010957 | SCC112_exon_17 | TGTAAAACGACGGCCAGTAACTCAGAGGTTGGTCAACAG | CAGGAAACAGCTATGACCTGCTATATCAACCAATAATTTCATC |
| 1010956 | SCC112_exon_18 | TGTAAAACGACGGCCAGTCATCATCTAATTTCCAACACAAGTTT | CAGGAAACAGCTATGACCGAGTCAGTTGTCATTGCTCACTT |
| 1015397 | SCC112_exon_19 | TGTAAAACGACGGCCAGTCAAAGTTGTCTTTCTTCCCTCCT | CAGGAAACAGCTATGACCAGGCTGTCTGTTAGTTGTGTCAAG |
| 1010954 | SCC112_exon_20 | TGTAAAACGACGGCCAGTCCCTTCAATATGAGGTGTTTGA | CAGGAAACAGCTATGACCAGGGTTGCTTGTTATTTTGCAT |
| 1010953 | SCC112_exon_21 | TGTAAAACGACGGCCAGTAGCTACCATGAAATAAGGAAGTAGTTT | CAGGAAACAGCTATGACCGGTATAGCAAATCCATTGTTGTAGA |
| / | SCC112_exon_22 | Not targeted | Not targeted |
| 1010951 | SCC112_exon_23 | TGTAAAACGACGGCCAGTTTAATTGAACCAAATGTTGATGCT | CAGGAAACAGCTATGACCAATACATTTACAGATCTAAGAAAAGTGC |
| 1010950 | SCC112_exon_24;SCC112_exon_25 | TGTAAAACGACGGCCAGTATGCAGCTTCTGAGCAAATATC | CAGGAAACAGCTATGACCGGGTTGTGGTGAAGTATCTAAGC |
| / | SCC112_exon_25 | Not targeted | Not targeted |
| 1010948 | SCC112_exon_26 | TGTAAAACGACGGCCAGTAGCTTAACTAGCTGCAAACAAACA | CAGGAAACAGCTATGACCCTGCATTAACTCTGGCAAACTG |
| 1015395 | SCC112_exon_27 | TGTAAAACGACGGCCAGTTAACACTCATCTTACTTCATTTGTCTTG | CAGGAAACAGCTATGACCACCATATCCAGCTAGGGTGAA |
| 1010946 | SCC112_exon_28 | TGTAAAACGACGGCCAGTTTTCTTTACTCTGTGGTCAAGACAAT | CAGGAAACAGCTATGACCTTTTAGAATGTTTACAAAAGTAAATGTG |
| / | SCC112_exon_29 | Failed primer design | Failed primer design |
| 1010945 | SCC112_exon_30 | TGTAAAACGACGGCCAGTAAAAACCCAGAAAAGAAAATACTAAAA | CAGGAAACAGCTATGACCAGTAGATGGGACTACCGGCTTA |
| 1010944 | SCC112_exon_31 | TGTAAAACGACGGCCAGTCATGTGCATTGAAGTGTTGG | CAGGAAACAGCTATGACCAGCAAAAATGAAGCCCAAGATA |
| 1010943 | SCC112_exon_32 | TGTAAAACGACGGCCAGTTGCAGGTTTCTCTCTCAAATG | CAGGAAACAGCTATGACCTTAACTGGAAGCCCTCAAATC |
| 1010942 | SCC112_exon_33 | TGTAAAACGACGGCCAGTAGAGTACCCAGCCAGGAAGTTT | CAGGAAACAGCTATGACCCCTGTTTAGATTTTTGCAGGAGA |
| 1010803 | SGOL1_exon_03;SGOL1_exon_02 | TGTAAAACGACGGCCAGTTGCTGGAGCACTAAAGAAGGAT | CAGGAAACAGCTATGACCTGGTTAACTATCTACTTTGAGCTAATTC |
| 1010802 | SGOL1_exon_04 | TGTAAAACGACGGCCAGTTCAGTAGAGAAAGCAACAGACTACAAA | CAGGAAACAGCTATGACCATTATGTGGCCCCTGCATGT |
| 1010801 | SGOL1_exon_05 | TGTAAAACGACGGCCAGTAGCCAAAGATTCAGATTACAGATTT | CAGGAAACAGCTATGACCCAGAAGAGTTCAATAGTTAAGCACCAT |
| 1015371 | SGOL1_exon_06 | TGTAAAACGACGGCCAGTCAAGTGGGCAAATAGAGGTCAT | CAGGAAACAGCTATGACCTGAATTCGAAAGAGTTGGATTTT |
| 1015372 | SGOL1_exon_06 | TGTAAAACGACGGCCAGTAAACGTTCCTGGCTGAATC | CAGGAAACAGCTATGACCCCTACTATTCCTCAAGACACACTGG |
| 1015370 | SGOL1_exon_06 | TGTAAAACGACGGCCAGTTTAGGTCAGAGCAATACAACTGTG | CAGGAAACAGCTATGACCTCATCTTACTCCTTTCCGACAAA |
| 1015373 | SGOL1_exon_06 | TGTAAAACGACGGCCAGTGCCAAATGACTGGTTTCAAAAT | CAGGAAACAGCTATGACCTCACATGTCAGATATAAGCCATCA |
| 1010798 | SGOL1_exon_07;SGOL1_exon_08 | TGTAAAACGACGGCCAGTTGTTTATGAGCTAGGGTCCTGTC | CAGGAAACAGCTATGACCTCATATTCAGTAAAAATTGTTTTGTAGC |
| 1010797 | SGOL1_exon_09 | TGTAAAACGACGGCCAGTACACGTACTGATTCCTCGGTCA | CAGGAAACAGCTATGACCTGTGTATAACCTGTGTCTCTTAACTCC |
| 1010941 | SMC1L1_exon_01 | TGTAAAACGACGGCCAGTCTGGAAGGGGTTCGAGTTAGAG | CAGGAAACAGCTATGACCGAGGGGCATAATCCTGCTGT |
| 1010940 | SMC1L1_exon_02;SMC1L1_exon_3 | TGTAAAACGACGGCCAGTGGTGCTACATTAGTTCAAGGGAGA | CAGGAAACAGCTATGACCAGGTTTGGTTGTGCTCAGTCTT |
| 1010939 | SMC1L1_exon_04 | TGTAAAACGACGGCCAGTCACTGGCTCCATCCTGGTC | CAGGAAACAGCTATGACCGTTTCCCTCCAGCTTCCAA |
| 1010938 | SMC1L1_exon_04;SMC1L1_exon_5 | TGTAAAACGACGGCCAGTGTCAAGAAATGTGCCTGGTTCT | CAGGAAACAGCTATGACCTCGACCTTTACTAGGGTGCTGT |
| 1010937 | SMC1L1_exon_07;SMC1L1_exon_6 | TGTAAAACGACGGCCAGTAAAATGCTTAGGACCGGAAAC | CAGGAAACAGCTATGACCAAGCTCTCCTTTGGGTGAAAAG |
| 1010936 | SMC1L1_exon_09;SMC1L1_exon_8 | TGTAAAACGACGGCCAGTTGCAGTTCTTCCTCTTAGAAGC | CAGGAAACAGCTATGACCTGGATGAGTGGGCACATAAAG |
| 1010935 | SMC1L1_exon_10;SMC1L1_exon_11;SMC1L1_exon_12 | TGTAAAACGACGGCCAGTAATAGGGTTCCATCCAGTGC | CAGGAAACAGCTATGACCCTAGTCTGTCCCCCTTTCCATA |
| 1010934 | SMC1L1_exon_13;SMC1L1_exon_11;SMC1L1_exon_12 | TGTAAAACGACGGCCAGTAGGAGACGGGGAAGTGAAAC | CAGGAAACAGCTATGACCTGCAGTATGCTTGTGGCAAT |
| 1010933 | SMC1L1_exon_15;SMC1L1_exon_14 | TGTAAAACGACGGCCAGTCTAAGAGCTGACCCTTGCCTAT | CAGGAAACAGCTATGACCGGATTGAAGCTGGCTGAACT |
| 1010932 | SMC1L1_exon_16 | TGTAAAACGACGGCCAGTAGGGACTATTTGAAAGTCTTCTTGA | CAGGAAACAGCTATGACCCCATAGCTGGTAGAATCAGATGG |
| 1010931 | SMC1L1_exon_17 | TGTAAAACGACGGCCAGTAAAAAGGTCCAGGGGCTAGG | CAGGAAACAGCTATGACCAAAAACAACAACAAATGTTACCATAC |
| 1010930 | SMC1L1_exon_18;SMC1L1_exon_17 | TGTAAAACGACGGCCAGTCCATTATTACCTTTCGCCTCAC | CAGGAAACAGCTATGACCCACCTATTGTCCTGTTCCCACT |
| 1010929 | SMC1L1_exon_19 | TGTAAAACGACGGCCAGTCTCTCTGGACAACTAGGAAGATAGT | CAGGAAACAGCTATGACCACAATGTTAGTAAGTGTAGACCTCAAAT |
| 1010928 | SMC1L1_exon_20 | TGTAAAACGACGGCCAGTTGTTTAGGGCTCCTGAGAGTTT | CAGGAAACAGCTATGACCACGTGAATTGCCTTACTTATTGTG |
| 1010927 | SMC1L1_exon_21;SMC1L1_exon_22 | TGTAAAACGACGGCCAGTTTCCACCAAACCATCACCTT | CAGGAAACAGCTATGACCAGTGGGATGTTCCTGCTCTG |
| 1010926 | SMC1L1_exon_23;SMC1L1_exon_24 | TGTAAAACGACGGCCAGTAGGACCTGATTCCCTATGCT | CAGGAAACAGCTATGACCAAGAAGTGGAGTGGGAAGCTG |
| 1010925 | SMC1L1_exon_25 | TGTAAAACGACGGCCAGTGAGGCCAGAATACTCCCTGTC | CAGGAAACAGCTATGACCAGTCCTACTCCCTGCCTGATTT |
| 1015404 | STAG2_exon_07 | TGTAAAACGACGGCCAGTGTGTGACCATAGACTTCTTAGTGTTAGA | CAGGAAACAGCTATGACCACTACAGGCACACACCACGACT |
| 1015409 | STAG2_exon_26 | TGTAAAACGACGGCCAGTTGTTTTTCCCTTTTCAAATTCTC | CAGGAAACAGCTATGACCGGGGCCATCTCCAATATTCTAA |
| / | STAG2_exon_02 | Failed primer design | Failed primer design |
| 1010897 | STAG2_exon_03 | TGTAAAACGACGGCCAGTTCCTTTCCGAATATTTTTGGTG | CAGGAAACAGCTATGACCTTAGCTTTTCAGATGGGGGTAA |
| 1015403 | STAG2_exon_04 | TGTAAAACGACGGCCAGTGAAGTTTTGTAATGAGTTAACCAAGC | CAGGAAACAGCTATGACCAGAACATTGCATTCTGCTTCAC |
| 1010899 | STAG2_exon_05 | TGTAAAACGACGGCCAGTTTGTTATAAGTGGATGGAATTCTT | CAGGAAACAGCTATGACCAGCAGAAAATGAATCAAAACACA |
| 1010900 | STAG2_exon_06 | TGTAAAACGACGGCCAGTAGAAGATTGATAGGTCATTTTGTGTT | CAGGAAACAGCTATGACCTGGCACTTTTTAACTCCCTACA |
| 1010902 | STAG2_exon_08 | TGTAAAACGACGGCCAGTTCTCTTGCCTACTTTGAGGATTT | CAGGAAACAGCTATGACCTCCAATGCATACCTATAGTCCAA |
| 1010903 | STAG2_exon_09 | TGTAAAACGACGGCCAGTGCAGATTAGCTCATTTCTGCTT | CAGGAAACAGCTATGACCTGAATTTGGTGGTCAGTCATTA |
| 1010904 | STAG2_exon_10 | TGTAAAACGACGGCCAGTTTCAAAATTCCCCAAAATACTG | CAGGAAACAGCTATGACCTGGAAGCTGAATAAAAATACTACCA |
| 1015405 | STAG2_exon_11 | TGTAAAACGACGGCCAGTACTTGGCATCTCTTGAATAAACC | CAGGAAACAGCTATGACCAATGGTTCTTCTTCGTGCCCTA |
| 1015406 | STAG2_exon_12 &13 | TGTAAAACGACGGCCAGTAACAAAGAGCTTAATTCCAAACTG | CAGGAAACAGCTATGACCTCAACTTCTAGCTGTAAACCTCCA |
| / | STAG2_exon_14 | Not targeted | Not targeted |
| 1010907 | STAG2_exon_15 | TGTAAAACGACGGCCAGTGCTCATGCTATCCATAGTAAAAGGA | CAGGAAACAGCTATGACCGAGTGGGTGGCTCTCCATTCT |
| 1010908 | STAG2_exon_16 | TGTAAAACGACGGCCAGTGCAAACTAAGGCAGTTTCTTCTC | CAGGAAACAGCTATGACCGAAAGAGTTAGCTTATTTCTTCTCCCTA |
| 1010909 | STAG2_exon_17 | TGTAAAACGACGGCCAGTAGCACTAACAGATAGGCAAGAGA | CAGGAAACAGCTATGACCTTTCAGTTATGCACACACAAACA |
| 1010910 | STAG2_exon_19;STAG2_exon_18 | TGTAAAACGACGGCCAGTTGTTATTTCAGGTGGCTTTTTG | CAGGAAACAGCTATGACCGAAGCAAAGGGAAGCATCATTA |
| 1010911 | STAG2_exon_20 | TGTAAAACGACGGCCAGTTTTCCATGGTGGTATGGTCA | CAGGAAACAGCTATGACCCATAATGTCCTTCCCTAACCTACA |
| 1010912 | STAG2_exon_21 | TGTAAAACGACGGCCAGTGAGGGTTTTTCCCTGTTTATCTG | CAGGAAACAGCTATGACCTTATTAAAAAGCTAAGCCTAATGACA |
| 1010913 | STAG2_exon_23;STAG2_exon_22 | TGTAAAACGACGGCCAGTTTTCAAAGTGGGATTTATTTGC | CAGGAAACAGCTATGACCTGATCATGAATTTGGAACATCAC |
| 1010914 | STAG2_exon_24 | TGTAAAACGACGGCCAGTCAATTTGGTGAGATTTGTAAGACA | CAGGAAACAGCTATGACCATCAGGATGAGCGAGAATGC |
| 1015408 | STAG2_exon_25 | TGTAAAACGACGGCCAGTTTTTTAAATAGGCCTTCACTATTCT | CAGGAAACAGCTATGACCTGACTCAATTTTTAACAGTTTTATGAGG |
| 1010915 | STAG2_exon_25 | TGTAAAACGACGGCCAGTTTTTTAAATAGGCCTTCACTATTCT | CAGGAAACAGCTATGACCAAAAACCTACGGTATACAAGTACACAA |
| 1010917 | STAG2_exon_27 | TGTAAAACGACGGCCAGTGTAACATTCTTTCCTGCCTTTGA | CAGGAAACAGCTATGACCGTACAACGGTCATCGCAGA |
| 1010918 | STAG2_exon_28 | TGTAAAACGACGGCCAGTCACTTTTGGTCATTTGCATCA | CAGGAAACAGCTATGACCCATACCTGAGTTGCTGGGACTA |
| 1010919 | STAG2_exon_29 | TGTAAAACGACGGCCAGTTGGCAAAGGAAGTAGTGAGTG | CAGGAAACAGCTATGACCTAGTTGGGTTAAGGCACAAATG |
| 1010920 | STAG2_exon_30 | TGTAAAACGACGGCCAGTTGTCCTGTAAGGCTGAGTTTGA | CAGGAAACAGCTATGACCCTGAGTCCATTTCCCTATGC |
| 1010921 | STAG2_exon_31 | TGTAAAACGACGGCCAGTGACTCTAAGGCCAGGTCAGG | CAGGAAACAGCTATGACCGAAAGACCAATTTTTAGTTCCTTG |
| / | STAG2_exon_32 | Exon not targeted | Exon not targeted |
| 1010922 | STAG2_exon_33 | TGTAAAACGACGGCCAGTAGACAAGTAAACATTGGGTTTTGA | CAGGAAACAGCTATGACCCGGAATTTGTCTCAACAAGTAG |
| 1010923 | STAG2_exon_34 | TGTAAAACGACGGCCAGTGTCAGGTACACTTGGAATCACATA | CAGGAAACAGCTATGACCATTAAGGATGGTGGGAGTGGTA |
| 1010924 | STAG2_exon_35 | TGTAAAACGACGGCCAGTGACTTTCTTAACCCCTTTCCAA | CAGGAAACAGCTATGACCATCGATTCAAAAGCCAACTAA |
| 1010873 | STAG3_Exon_ 04 | TGTAAAACGACGGCCAGTAGAAAAGAAAACCTAAAAGAGGTTC | CAGGAAACAGCTATGACCTTCTATCACTCCAAGGATTCACC |
| 1015329 | STAG3_exon_02 | TGTAAAACGACGGCCAGTTCTAGAACCATTCCCTTGGA | CAGGAAACAGCTATGACCTGTCACTGTGCTGAACGAGTA |
| / | STAG3_exon_03 | Failed primer design | Failed primer design |
| 1015331 | STAG3_exon_05 | TGTAAAACGACGGCCAGTCTCCCAGGGTTGCTACTTACAC | CAGGAAACAGCTATGACCGCCCGGCCTAAATAAGTAAAAT |
| 1010876 | STAG3_exon_07 | TGTAAAACGACGGCCAGTGGGGAGAATACCAGGAAATGAG | CAGGAAACAGCTATGACCTAGAAGGAAAGAAGGGCAATGA |
| 1010875 | STAG3_exon_07;STAG3_exon_6 | TGTAAAACGACGGCCAGTGCCATGTCTACACCATAACCTG | CAGGAAACAGCTATGACCAGGAGGCTGTACTGGCACTGA |
| 1015332 | STAG3_exon_07;STAG3_exon_6 | TGTAAAACGACGGCCAGTGACCAGGGGGTCTTCTCATT | CAGGAAACAGCTATGACCCCCGAGTCCTACAAAAGGATGT |
| 1010877 | STAG3_exon_08 | TGTAAAACGACGGCCAGTTCTTTTATCCTAAACTTCAATCCAG | CAGGAAACAGCTATGACCAAAAACTGAGCAAAAGTGGAATTA |
| 1010878 | STAG3_exon_09 | TGTAAAACGACGGCCAGTAGTTGACTATACAGCTTCTTCCAG | CAGGAAACAGCTATGACCTATAAAACTCCCCTGCCCTTC |
| 1015333 | STAG3_exon_10 | TGTAAAACGACGGCCAGTCCATGAGAGGGAGTTATCTGG | CAGGAAACAGCTATGACCCCTTAGCACCTGCATTACCATC |
| 1010880 | STAG3_exon_12;STAG3_exon_11 | TGTAAAACGACGGCCAGTGTAGAGGGGACACCCAAGCTA | CAGGAAACAGCTATGACCAAATGAAGAGCGGAAGACAAAG |
| 1010881 | STAG3_exon_14;STAG3_exon_13 | TGTAAAACGACGGCCAGTCCTGAGTTGCAGTATGTTGAGG | CAGGAAACAGCTATGACCTCTGTGTATGTCACCTCGCTCT |
| 1010882 | STAG3_exon_14;STAG3_exon_13 | TGTAAAACGACGGCCAGTACTCTTTCTGCTTTTCTGTGG | CAGGAAACAGCTATGACCGTCCTATGCACAACAGCCAGAC |
| 1010883 | STAG3_exon_16;STAG3_exon_15 | TGTAAAACGACGGCCAGTGAGAAGGATGGGAGTGGACA | CAGGAAACAGCTATGACCGGGTAGAAGGAGCACCCTA |
| 1010884 | STAG3_exon_17;STAG3_exon_18;  STAG3_exon_19 | TGTAAAACGACGGCCAGTAAAAGAAATCTCGTGGGAGCTA | CAGGAAACAGCTATGACCCTCCAGGTGCTGAGAAGAAAAG |
| 1010885 | STAG3_exon_18;STAG3_exon_20;  STAG3_exon_19 | TGTAAAACGACGGCCAGTGCTTGGAGAAGGTAGGGAGATA | CAGGAAACAGCTATGACCAAAGAACAGAGGGAACCAACTC |
| 1010886 | STAG3_exon_21;STAG3_exon_20 | TGTAAAACGACGGCCAGTGACAATGGGACACCCCAAAC | CAGGAAACAGCTATGACCTAGAAATGTCACCGCTCCCTAA |
| 1010887 | STAG3_exon_23;STAG3_exon_24;  STAG3_exon_22 | TGTAAAACGACGGCCAGTAAATTAGGGAGCGGTGACATTT | CAGGAAACAGCTATGACCTCTGAGGGCTAAAGATGAGAAGT |
| 1010888 | STAG3_exon_23;STAG3_exon_24;  STAG3_exon_25 | TGTAAAACGACGGCCAGTGTGGCCTTCTGTGAACTCTG | CAGGAAACAGCTATGACCCCCCACCTGTAGTGCTATTATG |
| 1015334 | STAG3_Exon_26 | TGTAAAACGACGGCCAGTAGTTTGGGAGGGAGACATGATT | CAGGAAACAGCTATGACCCACTTCCTTGTTGGCGTTCC |
| 1015335 | STAG3_exon_27;STAG3_exon_28 | TGTAAAACGACGGCCAGTTGAGTAGAGAAGAGAATGCTGGAC | CAGGAAACAGCTATGACCGAGGAAGCTCAGACAAGGAGAA |
| 1015338 | STAG3_Exon_29 | TGTAAAACGACGGCCAGTCCCTTCAGGCTTTTGGTTCT | CAGGAAACAGCTATGACCAGATAAACCTTGAAAGAAATTAATACCC |
| 1010891 | STAG3_exon_29;STAG3_exon_28 | TGTAAAACGACGGCCAGTGGGTATGTGTGTCAAGGCATAG | CAGGAAACAGCTATGACCTTTGATTTTTAATACAGGTTTTCAGTTG |
| 1015337 | STAG3_exon_29;STAG3_exon_28 | TGTAAAACGACGGCCAGTGAGCTCCTTTCAGAGTTTTCC | CAGGAAACAGCTATGACCAGCCAACGACCACCTGCT |
| 1015336 | STAG3_exon_29;STAG3_exon_28 | TGTAAAACGACGGCCAGTGAGGTCTTGGAGGGAGGTCT | CAGGAAACAGCTATGACCCTGAGGGAGTGGCAGTAGGT |
| 1010892 | STAG3_exon_30;STAG3_exon_31 | TGTAAAACGACGGCCAGTTTTTCCATTCTCTTCCTGATTGA | CAGGAAACAGCTATGACCACCTGAAGGGTTGTGTGGAGT |
| 1010893 | STAG3_exon_31;STAG3_exon_32 | TGTAAAACGACGGCCAGTCTGAGTTCCCAGTTTGGTGTCT | CAGGAAACAGCTATGACCATGTCTGTATCCTGCCGTTCTT |
| 1010894 | STAG3_exon_33 | TGTAAAACGACGGCCAGTGTCAGCACAATCGGGAAACTAC | CAGGAAACAGCTATGACCGGGGAAACAGGCTAGGTTAGAA |
| 1010895 | STAG3_exon_33;STAG3_exon_34 | TGTAAAACGACGGCCAGTAGTCATTTAAGGGGCTCAGTCA | CAGGAAACAGCTATGACCCTACCCCAGAGGGAAAGGTTAG |
| 1010871 | TIMELESS_exon_02;  TIMELESS_exon_3 | TGTAAAACGACGGCCAGTGAAGGTCGCTCTGTAGGATCTG | CAGGAAACAGCTATGACCGGTGCTTAATTCATAGGCTGGA |
| 1010870 | TIMELESS_exon_05;  TIMELESS_exon_4;  TIMELESS_exon_2;  TIMELESS_exon_3 | TGTAAAACGACGGCCAGTGCCTGTGAAATAGGGAACCTG | CAGGAAACAGCTATGACCCTTGGGTACTTGGAGGGAGAC |
| 1010869 | TIMELESS_exon_06;  TIMELESS_exon_5;  TIMELESS_exon_4 | TGTAAAACGACGGCCAGTGCAGCTCAACCTAACCATGC | CAGGAAACAGCTATGACCCTCCCAGACTGATGGTGAACT |
| 1015392 | TIMELESS_exon_07 | TGTAAAACGACGGCCAGTCCATGGTTGACTGTGAGTAACTG | CAGGAAACAGCTATGACCAGCTGCCCTTTTTGGGCTAT |
| 1015391 | TIMELESS_exon_08 | TGTAAAACGACGGCCAGTTTCTATGCCCTCAGCATTCC | CAGGAAACAGCTATGACCTGTCTGCAGTTTCAGGCATC |
| 1010837 | TIMELESS_Exon_09 | TGTAAAACGACGGCCAGTGTGCCACCATGCTCAGTTAG | CAGGAAACAGCTATGACCGTGCCCGACCAACATTTTGTA |
| 1010866 | TIMELESS_exon_10 | TGTAAAACGACGGCCAGTCACCTACGAGTCCACATTCCAG | CAGGAAACAGCTATGACCAAATTAAAGGTTGGGTACAATGG |
| 1015390 | TIMELESS_Exon_11 | TGTAAAACGACGGCCAGTGGACAGCTTAGGGCAATGG | CAGGAAACAGCTATGACCATTCCATCATCATAGGACAGG |
| 1015389 | TIMELESS_exon_12;  TIMELESS_exon_13 | TGTAAAACGACGGCCAGTAGTACCTGCACCACCAGGTT | CAGGAAACAGCTATGACCCCATTGCCCTAAGCTGTCCT |
| 1015388 | TIMELESS_exon_12;  TIMELESS_exon_13 | TGTAAAACGACGGCCAGTCAAACCTCTTGCTGTCGTAGTTC | CAGGAAACAGCTATGACCAGATGAGGCTGTGAGGGAGAG |
| 1010864 | TIMELESS_exon_14 | TGTAAAACGACGGCCAGTGGAGTGGAAAACCCAGATTGTA | CAGGAAACAGCTATGACCTCCATGTGTCTTATTTGATCCTC |
| 1010863 | TIMELESS_exon_14;  TIMELESS_exon_15 | TGTAAAACGACGGCCAGTAGCCTTGATGAGACAACTCTCTTT | CAGGAAACAGCTATGACCCCTCTTTAGCTCTCTTGACATCCT |
| 1010862 | TIMELESS_exon_16;  TIMELESS_exon_17 | TGTAAAACGACGGCCAGTATCGCCACTCTAGGACCAACT | CAGGAAACAGCTATGACCGCTGATTTATAGTAATTTTTCACTTCTC |
| 1015387 | TIMELESS_exon_16;  TIMELESS_exon_17 | TGTAAAACGACGGCCAGTCTCCGACACCTGGACCAC | CAGGAAACAGCTATGACCCAGTTTGCATTCTGTTTCTGG |
| 1010861 | TIMELESS_exon_16;  TIMELESS_exon_17;  TIMELESS_exon_18 | TGTAAAACGACGGCCAGTCCCTCAACCTGCCTGTCTACT | CAGGAAACAGCTATGACCAAACAAATCCTCTCTGCTCCAC |
| 1010860 | TIMELESS_exon_19;  TIMELESS_exon_18 | TGTAAAACGACGGCCAGTTGATGGAGAAGGAAACTAGATACACA | CAGGAAACAGCTATGACCTTCAATCGTCTGCTTAGTGACC |
| 1010859 | TIMELESS_exon_22;  TIMELESS_exon_20;  TIMELESS_exon_21 | TGTAAAACGACGGCCAGTACAATATGGGTTCCTTTCCTGAT | CAGGAAACAGCTATGACCGTATCTCAGCCCCTCTCCTTTC |
| 1010858 | TIMELESS_exon_23;  TIMELESS_exon_22;  TIMELESS_exon_21 | TGTAAAACGACGGCCAGTAGATGGAGGATGCCAACTTTT | CAGGAAACAGCTATGACCGACCTTCTTTACTGCCTTAGTCCT |
| 1010857a | TIMELESS_exon_23 | TGTAAAACGACGGCCAGTCAGATGGTGAGTAAGCTGGAAA | CAGGAAACAGCTATGACCGAACAGCCTCAGGCAGGAAG |
| 1010857b | TIMELESS_exon_24 | TGTAAAACGACGGCCAGTGTGGTCAGGAACGAGGAGA | CAGGAAACAGCTATGACCGGTGAAGATATAAGGGAAGAACTGG |
| 1010856 | TIMELESS_exon_26;  TIMELESS_exon_24;  TIMELESS_exon_25 | TGTAAAACGACGGCCAGTCAAGACAATGAGAAGGACCAAGA | CAGGAAACAGCTATGACCTTAGCCAAATGGAGCGGAGT |
| 1015386 | TIMELESS_exon_27 | TGTAAAACGACGGCCAGTAGGAACTCGGTCCTCATATTCTC | CAGGAAACAGCTATGACCCACTGTGCTAGGCACTGGGTA |
| 1010855 | TIMELESS_exon_27;  TIMELESS_exon_28 | TGTAAAACGACGGCCAGTAAGAAGGTCCCATCAAATTCC | CAGGAAACAGCTATGACCAAAAACCTATAACTGGGAACCTC |
| 1010854 | TIMELESS_exon_27;  TIMELESS_exon_28;  TIMELESS_exon_29 | TGTAAAACGACGGCCAGTAAAAACTGGGAGAAACAAAGACTG | CAGGAAACAGCTATGACCGTAAAGAGCACCGAGCACAAG |
| / | TIPIN_exon 04 | Failed primer design | Failed primer design |
| 1010834 | TIPIN_exon_02 | TGTAAAACGACGGCCAGTTTTTACCACAGCCACCACAC | CAGGAAACAGCTATGACCTAAGCAGCTCTAATTGAAGTGG |
| 1010833 | TIPIN_exon_03 | TGTAAAACGACGGCCAGTGGTCCAAAGCAAGACCATAAGA | CAGGAAACAGCTATGACCTGTGGTAAAAGGTATTTGAGGAGA |
| 1010832 | TIPIN_exon_06;TIPIN_exon_5 | TGTAAAACGACGGCCAGTGATTCTGTCCCTAAACATGAAACA | CAGGAAACAGCTATGACCAATGACCCCAGTGATTTTGG |
| 1015393 | TIPIN_exon_07 | TGTAAAACGACGGCCAGTCTTAATATCACACTTTTCCACTCC | CAGGAAACAGCTATGACCGCAGATGAAGTTGCGGAGAAT |
| 1015394 | TIPIN_exon_07 | TGTAAAACGACGGCCAGTCATTTCCTAGGGTCTGACTATTACT | CAGGAAACAGCTATGACCTTTTTCAGGTCTGTCACCTTG |
| 1010830 | TIPIN_exon_08 | TGTAAAACGACGGCCAGTCATGGATTAGCCCTGCTTTG | CAGGAAACAGCTATGACCTTACTAAACTGAGCTTTTGAAGAGG |
| 1011039 | WDHD1_exon_02 | TGTAAAACGACGGCCAGTGGCCCGAATCAAACAATAATCT | CAGGAAACAGCTATGACCGCAACCGGCTAATTTCCAC |
| 1015347 | WDHD1_exon_03 | TGTAAAACGACGGCCAGTAAAAATGGTAGGGCAAGTTACG | CAGGAAACAGCTATGACCGCTGGTACTACAGGTACACATAACCA |
| 1011037 | WDHD1_exon_04 | TGTAAAACGACGGCCAGTAAGCCCAAGTTTGTCCTCAAAA | CAGGAAACAGCTATGACCGTGCCTTCTGATGCCTTCTC |
| 1011036 | WDHD1_exon_06;WDHD1_exon_5 | TGTAAAACGACGGCCAGTTTATCATTCCCCTTGTGATTAGG | CAGGAAACAGCTATGACCAATATTTGGATCATAGCTTTTAGGA |
| 1011035 | WDHD1_exon_07 | TGTAAAACGACGGCCAGTAAGAAAAGAAAAGAAAAATAAGAATCAC | CAGGAAACAGCTATGACCAAGGAGGTACGAAATAAGGTGTT |
| 1011034 | WDHD1_exon_08 | TGTAAAACGACGGCCAGTAAAGACCTATTTAGTATCCAGTTATTGA | CAGGAAACAGCTATGACCAAGTTTTAAGTAATGTTCTGAGTTTCAT |
| 1011033 | WDHD1_exon_09 | TGTAAAACGACGGCCAGTAGGTTCTAAAAATACTGTCAAGAAAAA | CAGGAAACAGCTATGACCGAGCTTCAGAGTTGAAAGTTGC |
| 1011032 | WDHD1_exon_10;WDHD1_exon_9 | TGTAAAACGACGGCCAGTCTTGGAAAGGCATTATATGGAGA | CAGGAAACAGCTATGACCTGGTCTAATCATAGTTTGGAATGTG |
| 1011031 | WDHD1_exon_11 | TGTAAAACGACGGCCAGTGCAGAAGCTTGGATTATAGATTTGA | CAGGAAACAGCTATGACCTTTTCTAGGGAATGACTTCTGAGG |
| 1015346 | WDHD1_exon_12 | TGTAAAACGACGGCCAGTAGGTTGAAACAAGATTAAAACTGTGA | CAGGAAACAGCTATGACCGAGGCAAATAGCATGTCAGTGT |
| 1011029 | WDHD1_exon_13 | TGTAAAACGACGGCCAGTTGTTCTGAGGTATTTTGGGTGT | CAGGAAACAGCTATGACCATGGTTTGGTTGTTCAAAGGAC |
| 1015344 | WDHD1_exon_14 | TGTAAAACGACGGCCAGTTTTTCATTTTCATTGCTGCTAGT | CAGGAAACAGCTATGACCAAGTTTGATTGTTTCTGTCTTGTTTT |
| 1015343 | WDHD1_exon_15 | TGTAAAACGACGGCCAGTAGCTCCCAGTTCTGCCAAC | CAGGAAACAGCTATGACCAACATCTTACTGTGTTTCTAATACCTGA |
| 1011026 | WDHD1_exon_16 | TGTAAAACGACGGCCAGTCATGTTTCAAGTAAAGCAAGTGA | CAGGAAACAGCTATGACCCAGCAGGTGGCATAGTTTTC |
| 1011025 | WDHD1_exon_17 | TGTAAAACGACGGCCAGTGACAGTCACACCCATTTACTTTG | CAGGAAACAGCTATGACCGATCACACCACCGTGCTTTAG |
| 1011024 | WDHD1_exon_18 | TGTAAAACGACGGCCAGTGAATCTTAGGTTGTGGGACTTAG | CAGGAAACAGCTATGACCTGGGGAAAGACTTACTCTCAGG |
| 1011022 | WDHD1_exon_19;WDHD1_exon_20;WDHD1_exon_21 | TGTAAAACGACGGCCAGTCCATAGCTTCACAAACAACATCT | CAGGAAACAGCTATGACCCAGCCGAATTGACAGCAAC |
| 1011023 | WDHD1_exon_19;WDHD1_exon_20;WDHD1_exon_21 | TGTAAAACGACGGCCAGTCACCTGACTTAGCTGAAACATCA | CAGGAAACAGCTATGACCTTTTTGGATTAGCTTTCTTGTAAA |
| 1011021 | WDHD1_exon_22 | TGTAAAACGACGGCCAGTAAAAGACAACTCTCAGCCCAAG | CAGGAAACAGCTATGACCCGAGAGAAAATGGTGACTGG |
| 1011020 | WDHD1_exon_23;WDHD1_exon_22 | TGTAAAACGACGGCCAGTTGTAAACAATCAGAACCTCCTATCC | CAGGAAACAGCTATGACCTAGCAGCCAAGGACGAGTAAAT |
| 1015342 | WDHD1_exon_24 | TGTAAAACGACGGCCAGTGACATTAATATTTGTGGCTTCCTT | CAGGAAACAGCTATGACCTTTTTGGTTTTGCATGTGTT |
| 1015341 | WDHD1_exon_25 | TGTAAAACGACGGCCAGTTCAAGTGATCCGCCTCGAC | CAGGAAACAGCTATGACCTATAATAGATGTCAGAGACTTATGGTTG |
| 1011017 | WDHD1_exon_26 | TGTAAAACGACGGCCAGTCTGCATTTGGAGGCAGAGTAA | CAGGAAACAGCTATGACCGTTTCATTTTTAAATAGTACTGCAATTA |
| * Primers have unique tails | | |  |
