# Supplementary material for: Sequencing of Candidate Chromosome Instability Genes in Endometrial Cancers Reveals Somatic Mutations in ESCO1, CHTF18, and MRE11A
Source: PLoS One. 2013 Jun 3;8(6):e63313. doi: 10.1371/journal.pone.0063313 (PMC3670891; doi:10.1371/journal.pone.0063313)
Supplement: Table S3 — PCR primers used to amplify and sequence CHTF18, ESCO1, and MRE11A within the validation screen. (DOC) [file pone.0063313.s010.doc]

| **Table S3. PCR primers used to amplify and sequence *CHTF18,* *ESCO1,* and *MRE11A* within the validation screen** | | |
| --- | --- | --- |
| **Region of Interest (Gene_Exon)** | **Sense primer (5'-3')** | **Antisense primer (5'-3')** |
| ***PCR primers*** | | |
| CHTF18_EXON_1 | NOT TARGETED | NOT TARGETED |
| CHTF18_EXON_2 | NOT TARGETED | NOT TARGETED |
| CHTF18_EXON_3 | TGTAAAACGACGGCCAGTCAGGAAGAGGCAGGTGGA | CAGGAAACAGCTATGACCAGCACGCAGCACCAGATAAG |
| CHTF18_EXON_4 | TGTAAAACGACGGCCAGTCCGTGCCCTGGATGAG | CAGGAAACAGCTATGACCTGCACAGGACCCGCTTT |
| CHTF18_EXON_5 | TGTAAAACGACGGCCAGTATCTGGATGGCTTCATTCCTTT | CAGGAAACAGCTATGACCACGCTAGGAGCTAGGAGTTGGT |
| CHTF18_EXON_6 | TGTAAAACGACGGCCAGTACCTGCTGGGTGTGTCCTT | CAGGAAACAGCTATGACCCCCGACCTGAGACTGGAGA |
| CHTF18_EXON_7 | TGTAAAACGACGGCCAGTTTGGCTCTTGCACCAACTC | CAGGAAACAGCTATGACCCTCAGTTCACCACCTACAACTCAA |
| CHTF18_EXON_8 | TGTAAAACGACGGCCAGTCATGGTGGCAGGTGGACT | CAGGAAACAGCTATGACCCCTTCAGGGACAACGACTTC |
| CHTF18_EXON_9 | TGTAAAACGACGGCCAGTTTGGTGTGTGAAAGTGCTCAA | CAGGAAACAGCTATGACCCCGAGGAAGGACACGTTTT |
| CHTF18_EXON_10 | TGTAAAACGACGGCCAGTACGGTCTCCACGCAAATG | CAGGAAACAGCTATGACCAGCCTCTCCTGACTGTGG |
| CHTF18_EXON_11 | TGTAAAACGACGGCCAGTGTCTTCCGCACACGCATC | CAGGAAACAGCTATGACCGGGAAGTGGAGCAGGAAGG |
| CHTF18_EXON_12 | TGTAAAACGACGGCCAGTCGCCACAGTCAGGAGAGG | CAGGAAACAGCTATGACCAGGGAGACCTGGTGGAGAAG |
| CHTF18_EXON_13 | TGTAAAACGACGGCCAGTGAGCCGTGTGGCTGATG | CAGGAAACAGCTATGACCCTCTGCGCTGGTCCTTG |
| CHTF18_EXON_14 | TGTAAAACGACGGCCAGTAGCAGCCCTTCTCCACCA | CAGGAAACAGCTATGACCGCGAGAACAAGCCAGGAA |
| CHTF18_EXON_15 | TGTAAAACGACGGCCAGTGTCTTTGGCTGTCTCCACCT | CAGGAAACAGCTATGACCGAACCTGCCTCTCTCAGCAC |
| CHTF18_EXON_16 | TGTAAAACGACGGCCAGTAGTGACCCCTTGCTGGTGT | CAGGAAACAGCTATGACCGGAAGGTGATCCTGGGTGT |
| CHTF18_EXON_18;CHTF18_EXON_17 | TGTAAAACGACGGCCAGTGACCAGGCCTTGGCTCAC | CAGGAAACAGCTATGACCGCTCTGCAGCAAAACTCAGGT |
| CHTF18_EXON_19 | TGTAAAACGACGGCCAGTCTCTGGCCTTGTGGCTTT | CAGGAAACAGCTATGACCCCATTCCACACCTGTTCCTC |
| CHTF18_EXON_20 | TGTAAAACGACGGCCAGTGAGATCGAGGTGGAGAAGATG | CAGGAAACAGCTATGACCCCTAGCCACAGCCCTGAA |
| CHTF18_EXON_21 | NOT TARGETED | NOT TARGETED |
| CHTF18_EXON_22 | AGCTGAGGAGCAACCCTGTG | ACATCTCCAGCTTCCTGTCTCT |
| ESCO1_EXON_04 | TGTAAAACGACGGCCAGTCTCTCGCTAACAACTTAACAGG | CAGGAAACAGCTATGACCGAATCTTCACAAATGGAAAGTG |
| ESCO1_EXON_04 | TGTAAAACGACGGCCAGTGTGAAAAACTGGTTCGTAATAAGC | CAGGAAACAGCTATGACCCGCAAGGTAGAACATCAGACAG |
| ESCO1_EXON_04 | TGTAAAACGACGGCCAGTCAGCCAGAATTGGAAACACGC | CAGGAAACAGCTATGACCTTTCGGGACCACTGAAGTAGC |
| ESCO1_EXON_04 | TGTAAAACGACGGCCAGTAACGACCTTCTTGAAACCTCTG | CAGGAAACAGCTATGACCCCTTGTCCAAAGTAAAGCAAGTG |
| ESCO1_EXON_05 | TGTAAAACGACGGCCAGTTCTGAAAGCATGGGTGATAGTC | CAGGAAACAGCTATGACCAGACAGACGAAACTTGGAAAAA |
| ESCO1_EXON_06 | TGTAAAACGACGGCCAGTTGTCCATAGTAAACAGTATGTATAGGC | CAGGAAACAGCTATGACCTGTTTTCATTTGTTTCTACTTAGAGATT |
| ESCO1_EXON_07 | TGTAAAACGACGGCCAGTAGTCTGGCTTCCTTGGTTTC | CAGGAAACAGCTATGACCTTTTTCCCCTTCAAATGATGT |
| ESCO1_EXON_08 | TGTAAAACGACGGCCAGTACATACTACACGTGCTTTGTACATATT | CAGGAAACAGCTATGACCGGGGCTTTAGGACTAAGTTTGTAA |
| ESCO1_EXON_09 | FAILED PRIMER DESIGN | FAILED PRIMER DESIGN |
| ESCO1_EXON_10 | TGTAAAACGACGGCCAGTTTCAGTAGAGAACAGGTTTTCACAAT | CAGGAAACAGCTATGACCAACTCTTTAGAGGATGCCCTGCT |
| ESCO1_EXON_11 | TGTAAAACGACGGCCAGTACTTACAGATCCAATACACTGATAACC | CAGGAAACAGCTATGACCGTGCGGGAGGTAGAGAGAACTA |
| ESCO1_EXON_ 12 | TGTAAAACGACGGCCAGTGAGCACATACAGCAGTATCTAA | CAGGAAACAGCTATGACCCATTAGTTTTCAGTCCACTATG |
| MRE11A_EXON_02 | TGTAAAACGACGGCCAGTTCAAATTACTGCAAGACTCCAATC | CAGGAAACAGCTATGACCGAACCAGAACCGTATGTGACC |
| MRE11A_EXON_03 | TGTAAAACGACGGCCAGTTGAATTTAAGACACAAAGCATACAAA | CAGGAAACAGCTATGACCCCTGGAAGAGTACGAAGTCAGAT |
| MRE11A_EXON_04 | TGTAAAACGACGGCCAGTTTGGAGGAGAATCTTAGGGAAA | CAGGAAACAGCTATGACCAAACACTGACAAACTGTAAACCAC |
| MRE11A_EXON_05 | FAILED PRIMER DESIGN | FAILED PRIMER DESIGN |
| MRE11A_EXON_06 | TGTAAAACGACGGCCAGTTCAGGATTCTACACCTGAGTCTAAA | CAGGAAACAGCTATGACCCAGGCTTTAAGACGGTTGCTTA |
| MRE11A_EXON_07 | TGTAAAACGACGGCCAGTAAATCTATGTTTTGTCTGATCTTGC | CAGGAAACAGCTATGACCAAGTGAAATACTTTGAGAAGGACA |
| MRE11A_EXON_08 | TGTAAAACGACGGCCAGTTGAGCAGCAAAATAACACCAAT | CAGGAAACAGCTATGACCGGGGAAATCCTTCCTATGTAAAA |
| MRE11A_EXON_09 | TGTAAAACGACGGCCAGTCAGTGTCCTTACAGGCTTCA | CAGGAAACAGCTATGACCGATCAGACAGGGATAATAATGGA |
| MRE11A_EXON_10 | TGTAAAACGACGGCCAGTCCGATGGTGATTGCTCTTCTTA | CAGGAAACAGCTATGACCGATCCCTAAATCTTCTGGTGAG |
| MRE11A_EXON_11 | TGTAAAACGACGGCCAGTACAATCATATTAAAACATCTTCCATT | CAGGAAACAGCTATGACCTGTTCCTAGCATTCATCTTTCTCTT |
| MRE11A_EXON_12 | TGTAAAACGACGGCCAGTAAACACTAATTTTCCCTGCTGTG | CAGGAAACAGCTATGACCGCAGTACCCATGATTGCTTTT |
| MRE11A_EXON_13 | TGTAAAACGACGGCCAGTCTATCCATGGGGAACAAAACAC | CAGGAAACAGCTATGACCTCATAGCTGCCATTTTTGGACT |
| MRE11A_EXON_14 | TGTAAAACGACGGCCAGTCAGGTTTTAGACAAGAACAAAATGG | CAGGAAACAGCTATGACCAATTGGAAAAATATGATTTACTTTTGTG |
| MRE11A_EXON_15 | TGTAAAACGACGGCCAGTAGTGAAGCTTTTATAAGGTATGTGC | CAGGAAACAGCTATGACCAGCCACTGTGTCAGCCTCCT |
| MRE11A_EXON_16 | TGTAAAACGACGGCCAGTCCTGTGATCCTAATTGCCCTTAT | CAGGAAACAGCTATGACCTCATGCCAGTTAATTTTTGTATTTTT |
| MRE11A_EXON_17 | TGTAAAACGACGGCCAGTAAAATCAAATCCTAGAAGCCCTA | CAGGAAACAGCTATGACCACAGTATCTCAATAAGCTGGGAAA |
| MRE11A_EXON_18 | TGTAAAACGACGGCCAGTACTGAAAATCCTTGTACTAATGCTG | CAGGAAACAGCTATGACCGTATTTCCCACATGGTCTGATA |
| MRE11A_EXON_19 | TGTAAAACGACGGCCAGTCATGTGAAATGACTCTCACTGGTAT | CAGGAAACAGCTATGACCGAAACAAAGCTCTTACTACAACAACC |
| MRE11A_EXON_20 | TGTAAAACGACGGCCAGTACTTATGGAGTTATGCTCAGGAA | CAGGAAACAGCTATGACCGCTTGCTTTGCTAGGTTGTTTT |
| ***Sequencing primers*** | | |
| CHTF18_EXON_1 | / | / |
| CHTF18_EXON_2 | / | / |
| CHTF18_EXON_3 | NONE | CTCATCCAGGGCACGGGA |
| CHTF18_EXON_4 | TGTAAAACGACGGCCAGT | CAGGAAACAGCTATGACC |
| CHTF18_EXON_5 | TGTAAAACGACGGCCAGT | CAGGAAACAGCTATGACC |
| CHTF18_EXON_6 | TGTAAAACGACGGCCAGT | CAGGAAACAGCTATGACC |
| CHTF18_EXON_7 | TGTAAAACGACGGCCAGT | CAGGAAACAGCTATGACC |
| CHTF18_EXON_8 | TGTAAAACGACGGCCAGT | CAGGAAACAGCTATGACC |
| CHTF18_EXON_9 | TGTAAAACGACGGCCAGT | CAGGAAACAGCTATGACC |
| CHTF18_EXON_10 | TGTAAAACGACGGCCAGT | CAGGAAACAGCTATGACC |
| CHTF18_EXON_11 | CTTGATGCCTGGGTAGGTG | GTGCGAACCTGAGGAGAGG |
| CHTF18_EXON_12 | TGTGTCCTGGGCTGTGGT | GGGTTACCAGGGTTCCAT AND ATCAGCCACACGGCTCCT |
| CHTF18_EXON_13 | TGTAAAACGACGGCCAGT | CAGGAAACAGCTATGACC |
| CHTF18_EXON_14 | AGGAGTTGGCCGCTTCTC | CCCGCCTCGAAGGTAGTG |
| CHTF18_EXON_15 | TGTAAAACGACGGCCAGT | CACAGCCCAGGTCCTAGC |
| CHTF18_EXON_16 | GACCCTTGTGGAGGGTCTG | NONE |
| CHTF18_EXON_18;CHTF18_EXON_17 | TGTAAAACGACGGCCAGT | CAGGAAACAGCTATGACC |
| CHTF18_EXON_19 | TGTAAAACGACGGCCAGT | CAGGAAACAGCTATGACC |
| CHTF18_EXON_20 | TGTAAAACGACGGCCAGT | CAGGAAACAGCTATGACC |
| CHTF18_EXON_21 | / | / |
| CHTF18_EXON_22 | AGCTGAGGAGCAACCCTGTG | ACATCTCCAGCTTCCTGTCTCT |
| ESCO1_EXON_04 | TGTAAAACGACGGCCAGT | CAGGAAACAGCTATGACC |
| ESCO1_EXON_04 | TGTAAAACGACGGCCAGT | CAGGAAACAGCTATGACC |
| ESCO1_EXON_04 | TGTAAAACGACGGCCAGT | CAGGAAACAGCTATGACC |
| ESCO1_EXON_04 | TGTAAAACGACGGCCAGT | CAGGAAACAGCTATGACC |
| ESCO1_EXON_05 | TGTAAAACGACGGCCAGT | CAGGAAACAGCTATGACC |
| ESCO1_EXON_06 | TGTAAAACGACGGCCAGT | CAGGAAACAGCTATGACC |
| ESCO1_EXON_07 | TGTAAAACGACGGCCAGT | CAGGAAACAGCTATGACC |
| ESCO1_EXON_08 | TGTAAAACGACGGCCAGT | CAGGAAACAGCTATGACC |
| ESCO1_EXON_09 | / | / |
| ESCO1_EXON_10 | TGTAAAACGACGGCCAGT | CAGGAAACAGCTATGACC |
| ESCO1_EXON_11 | TGTAAAACGACGGCCAGT | CAGGAAACAGCTATGACC |
| ESCO1_EXON_ 12 | TGTAAAACGACGGCCAGT | CAGGAAACAGCTATGACC |
| MRE11A_EXON_02 | TGTAAAACGACGGCCAGT | CAGGAAACAGCTATGACC |
| MRE11A_EXON_03 | TGTAAAACGACGGCCAGT | CAGGAAACAGCTATGACC |
| MRE11A_EXON_04 | NONE | CAGGAAACAGCTATGACC |
| MRE11A_EXON_05 | / | / |
| MRE11A_EXON_06 | TGTAAAACGACGGCCAGT | CAGGAAACAGCTATGACC |
| MRE11A_EXON_07 | TGTAAAACGACGGCCAGT | CAGGAAACAGCTATGACC |
| MRE11A_EXON_08 | TGTAAAACGACGGCCAGT | CAGGAAACAGCTATGACC |
| MRE11A_EXON_09 | TGTAAAACGACGGCCAGT | CAGGAAACAGCTATGACC |
| MRE11A_EXON_10 | TGTAAAACGACGGCCAGT | CAGGAAACAGCTATGACC |
| MRE11A_EXON_11 | TGTAAAACGACGGCCAGT | CAGGAAACAGCTATGACC |
| MRE11A_EXON_12 | TGTAAAACGACGGCCAGT | CAGGAAACAGCTATGACC |
| MRE11A_EXON_13 | TGTAAAACGACGGCCAGT | CAGGAAACAGCTATGACC |
| MRE11A_EXON_14 | TGTAAAACGACGGCCAGT | CAGGAAACAGCTATGACC |
| MRE11A_EXON_15 | TGTAAAACGACGGCCAGT | CAGGAAACAGCTATGACC |
| MRE11A_EXON_16 | TGTAAAACGACGGCCAGT | CAGGAAACAGCTATGACC |
| MRE11A_EXON_17 | TGTAAAACGACGGCCAGT | CAGGAAACAGCTATGACC |
| MRE11A_EXON_18 | TGTAAAACGACGGCCAGT | CAGGAAACAGCTATGACC |
| MRE11A_EXON_19 | TGTAAAACGACGGCCAGT | CAGGAAACAGCTATGACC |
| MRE11A_EXON_20 | TGTAAAACGACGGCCAGT | CAGGAAACAGCTATGACC |
